# Supplementary material for: Comparison of high-intensity interval training versus moderate-intensity continuous training in pulmonary rehabilitation for interstitial lung disease: a randomised controlled pilot feasibility trial
Source: BMJ Open. 2023 Aug 22;13(8):e066609. doi: 10.1136/bmjopen-2022-066609 (PMC10445364; doi:10.1136/bmjopen-2022-066609)
Supplement: Supplementary data [file bmjopen-2022-066609supp001.pdf]

Supplementary file 1- Development of the ILD Booklet

All pulmonary rehabilitation programmes in the UK include an hour of education, usually at the end of the exercise session. This is a series of presentations and discussions on topics related to the disease(s) included in the programme. In our PR programme, we had a series of topics related to ILD, which included:

Topics about breathing and breathlessness:

- Mechanics of breathing,
- Helpful/unhelpful breathing patterns,
- The Active Cycle of Breathing technique,
- Useful positions for recovering,
- Pacing/ conserving energy in everyday activities

Topics about ILD:

- What happens with ILD,
- Cough- techniques that help
- Associated conditions-rheumatoid Arthritis, Gastro-Oesophageal Reflux disease, Sjögren's syndrome, Bird Fancier's Lung, Idiopathic Pulmonary Fibrosis

Topics about managing the condition:

- Why healthier lifestyle is important,
- Keeping a good posture,
- Barriers to activity/exercise,
- Dietary advice,
- Smoking and smoking cessation,
- Incontinence and advice on pelvic floor exercises
- Oxygen and traveling advice
- Relaxation information,
- Mood, anxiety and breathlessness,
- Self-management and goal setting.

Our 'ILD booklet' was a summary of the key points in the topics discussed. The booklet was put together by the two physiotherapists (included as authors in this manuscript) and the accuracy of the topics was checked by the health professionals who presented each session. For e.g. the topic on 'dietary advice for ILD' was checked by the dietitian in our hospital.

The booklet also included the exercises that were part of the PR programme, with pictures as reminders (the physiotherapists took pictures of themselves to show ILD patient the correct positioning during exercises) and how to adapt exercises in or around the home environment.

Supplementary file 1- Development of the ILD Booklet

Information on how to record progress during PR and in the 6-month period was presented with examples and copies of the breathlessness and exertion BORG scales:

Borg Breathlessness Scale (BRE)

0	Nothing at all
0.5	Very, very slight (just noticeable)
1	Very Slight
2	Slight
3	Moderate
4	Somewhat Severe
5	Severe
6	
7	Very Severe
8	
9	Very, very Severe (Almost Maximal)
10	Maximal

Borg Rate of Perceived Exertion (RPE)

6	No exertion at all
7	Extremely light
8	
9	Very light
10	
11	Light
12	
13	Somewhat Hard
14	
15	Hard (heavy)
16	
17	Very Hard
18	
19	Extremely Hard
20	Maximal Exertion

Example on how to record the information in your worksheet:

Date		BRE	RPE	SpO2	PR
	Start				
Treadmill	4:00 at 3kph	5	14	96	104

During PR sessions, participants used the workout sheet below to record their progress. The booklet included many empty sheets so that participants could record their workouts over the 6-month programme too.

Supplementary file 1- Development of the ILD Booklet

Name:	NHS No:	Transport Y/N	Interpreter: Y/N
O2:	Aid	Supervision	ILD Type:
	THR:	ID:	Sheet:

Date		BRE	RPE	SpO2	PR		BRE	RPE	SpO2	PR		BRE	RPE	SpO2	PR		BRE	RPE	SpO2	PR
	Start					Start					Start					Start				
Treadmill																				
Brisk walk																				
Bike																				
Trampet																				
Punch ups																				
Step ups																				
Knee raise																				
Hip ext																				
STS/Knee ext																				
Bicep curls																				
Arms to side																				
Wall push ups																				
	Middle					Middle					Middle					Middle				
	End					End					End					End				

The booklet also included information about community gyms and leisure centres around South London, sources of support for patients and carers (e.g. advice for carers, disability benefits helpline, citizen's advice, charities) and useful links for organised walks in parks.

Finally, the booklet included the contact numbers of the physiotherapists so that participants could call to ask questions if needed.
