# Supplementary material for: Comparison of high-intensity interval training versus moderate-intensity continuous training in pulmonary rehabilitation for interstitial lung disease: a randomised controlled pilot feasibility trial
Source: BMJ Open. 2023 Aug 22;13(8):e066609. doi: 10.1136/bmjopen-2022-066609 (PMC10445364; doi:10.1136/bmjopen-2022-066609)
Supplement: Supplementary data [file bmjopen-2022-066609supp002.pdf]

Supplementary file 2- Analysis of missing data patterns

Frequencies of the patterns of missing data overall – most common pattern highlighted.

PATTERN	6MWD	SGRQ-I	SNIP	QUADS_DOM
+++	26	33	27	32
++.	6	2	3	2
+.+	1	1	1	2
.++	4	0	3	0
**+..**	**19**	**21**	**18**	**20**
..+	0	0	1	0
.+.	0	1	1	1
...	2	0	4	1
TOTAL MISSING	32	25	31	26

Frequencies of the patterns of missing data stratified by intervention group – most common pattern highlighted.

	CONTROL (MICT)				INTERVENTION (HIIT)			
PATTERN	6MWD	SGRQ-I	SNIP	Q_DOM	6MWD	SGRQ-I	SNIP	Q_DOM
+++	15	16	14	15	11	17	13	17
++.	1	1	1	1	5	1	2	1
+.+	1	1	1	2	0	0	0	0
.++	1	0	1	0	3	0	2	0
**+..**	**6**	**7**	**6**	**7**	**13**	**14**	**12**	**13**
..+	0	0	1	0	0	0	0	0
.+.	0	0	0	0	0	1	1	1
...	1	0	1	0	1	0	3	1
TOT MISS	10	9	11	10	22	16	20	16

Frequencies of the patterns of missing data stratified by intervention group and clinical group – most common pattern highlighted.

	CONTROL (MICT)								INTERVENTION (HIIT)							
	FIBROSIS				AUTOIMMUNE				FIBROSIS				AUTOIMMUNE			
PATTERN	6MWD	SGRQ-I	SNIP	Q_DOM	6MWD	SGRQ-I	SNIP	Q_DOM	6MWD	SGRQ-I	SNIP	Q_DOM	6MWD	SGRQ-I	SNIP	Q_DOM
+++	3	4	3	4	12	12	11	11	8	11	8	11	3	6	5	6
++.	1	1	1	1	0	0	0	0	1	0	1	0	4	1	1	1
+.+	1	1	0	1	0	0	1	1	0	0	0	0	0	0	0	0
.++	1	0	1	0	0	0	0	0	2	0	1	0	1	0	1	0
**+..**	**5**	**5**	**5**	**5**	**1**	**2**	**1**	**2**	**9**	**9**	**8**	**8**	**4**	**5**	**4**	**5**
..+	0	0	1	0	0	0	0	0	0	0	0	0	0	0	0	0
.+.	0	0	0	0	0	0	0	0	0	0	0	0	0	1	1	1
...	0	0	0	0	1	0	1	0	0	0	1	1	1	0	1	0
TOTAL MIS	8	7	8	7	2	2	3	3	12	9	11	9	10	7	8	7

Nikoletou D, *et al. BMJ Open* 2023; 13:e066609. doi: 10.1136/bmjopen-2022-066609

Supplementary file 2- Analysis of missing data patterns

Analysis of the missing data for each outcome.

	**6MWD**				**SGRQ-I**				**SNIP**				**QUADS DOM**			
	OR	p-value	95%CI		OR	p-value	95%CI		OR	p-value	95%CI		OR	p-value	95%CI	
**MODELS ON TIME ONLY**																
**Time**	4.575	<.001	1.948	10.742	137.057	<.001	11.737	1600.393	4.132	0.001	1.814	9.412	76.820	<.001	8.511	693.382
**Time^2**	0.867	0.002	0.791	0.950	0.617	<.001	0.481	0.791	0.872	0.003	0.796	0.954	0.650	<.001	0.519	0.814
**MODELS ON INTERVENTION GROUP ADJUSTED FOR TIME AND TIME^2**																
**Intervention**	3.147	**0.108**	0.776	12.759	6.168	**0.212**	0.353	107.623	3.012	**0.172**	0.620	14.634	5.016	**0.259**	0.305	82.425
**MODELS ON INTERVENTION GROUP ADJUSTED FOR TIME AND TIME^2**																
**Intervention**	2.896	**0.135**	0.719	11.659	4.942	**0.276**	0.279	87.421	2.622	**0.224**	0.554	12.420	4.069	**0.324**	0.251	66.002
**Clinical**	0.459	**0.258**	0.119	1.769	0.140	**0.182**	0.008	2.506	0.304	**0.133**	0.064	1.439	0.167	**0.210**	0.010	2.745

OR= odds ratios

Missing observations were denoted by 1 if missing and 0 otherwise, which generated a longitudinal binary outcome which has been analysed using binary mixed models, i. e. accounting for the longitudinal aspect of the data.

The results are consistent with no pattern in the missing data across intervention and/or clinical groups and missingness is most likely attributable to the follow-up time.

Nikoletou D, *et al. BMJ Open* 2023; 13:e066609. doi: 10.1136/bmjopen-2022-066609
