# Supplementary material for: Comparison of high-intensity interval training versus moderate-intensity continuous training in pulmonary rehabilitation for interstitial lung disease: a randomised controlled pilot feasibility trial
Source: BMJ Open. 2023 Aug 22;13(8):e066609. doi: 10.1136/bmjopen-2022-066609 (PMC10445364; doi:10.1136/bmjopen-2022-066609)
Supplement: Supplementary data [file bmjopen-2022-066609supp008.pdf]

Supplementary Table 1- Descriptive values at each assessment point

Table 1: Descriptive values at each assessment point

	Control group (MICT) Mean (SD)			Intervention group (HIIT) Mean (SD)		
	**Baseline N= 25**	**2M (Post-PR) N=17**	**8M (6M post-PR) N= 17**	**Baseline N=33**	**2M (Post-PR) N=19**	**8M (6M post-PR) N=14**
**BMI (kg/m²)**	27.5 (3.8)	28.3 (4.1)	27.8 (3.9)	27.2 (5.1)	26.5 (4.9)	26.0 (4.7)
**Waist/Hip ratio**	0.9(0.1)	0.9 (0.1)	0.9 (0.0)	0.9 (0.1)	0.9 (0.1)	0.9 (0.1)
**%fat**	29.9 (9.3)	33.2 (9.8)	33.7 (8.7)	30.1 (8.1)	27.9 (10.7)	28.5 (9.6)
**FVC%,pred.**	85.8 (23.9)	88.0 (17.5)	88.7 (24.4)	73.8 (18.8)	68.4 (22.4)	71.3 (24.1)
**DLCO%, pred.**	53.9 (13.1)	53.6 (13.5)	56.5 (21.1)	41.7 (13.9)	44.9 (20.2)	44.8 (20.3)
**6MWD (m)**	380 (139.8)	453.9 (108.1)	437.4 (107.9)	364 (108.3)	434.3 (102.3)	421.9 (123.0)
**SNIP (cmH_2O)**	96.8 (25.9)	103.0 (21.04)	97.8 (21.4)	93.1 (24.5)	102.2 (34.1)	95.3 (29.5)
**PImax(cmH_2O)**	91.1(34.2)	104.3 (28.5)	104.9 (24.0)	91.8 (27.4)	92.8 (28.6)	95 (31.7)
**PEmax(cmH_2O)**	114.4 (40.3)	111.1 (33.3)	106.8 (34.5)	100.7 (23.5)	105.1 (24.2)	106.9 (24.7)
**Handgrip (kg)**	27.2 (11.2)	28.4 (8.4)	27.4 (9.3)	24.8 (9.4)	26.2 (11.4)	27.4 (11.5)
**Quads extension (dom) (kg)**	18.7 (6.2)	21.8 (5.0)	22.8 (6.1)	18.0 (5.0)	20.0 (5.6)	21.5 (5.4)
**Hip flexion (dom) (kg)**	15.3 (5.1)	17.9 (4.1)	18.5 (5.1)	15.9 (4.3)	16.5 (4.7)	18.1 (6.3)
**HAD-A**	6.2 (4.6)	4.8 (3.3)	5.1 (3.7)	6.4 (3.9)	6.4 (4.6)	7.2 (4.2)
**HAD-D**	5.8 (3.2)	5.0 (3.3)	5.2 (2.3)	5.2 (4.0)	5.1 (3.4)	6.6 (4.2)
**SGRQ-I Total score**	43.3 (20.3)	35.1 (15.3)	41.5 (19.3)	49.3 (21.8)	40.2 (16.7)	48.9 (21.6)
