# Supplementary material for: Comparison of high-intensity interval training versus moderate-intensity continuous training in pulmonary rehabilitation for interstitial lung disease: a randomised controlled pilot feasibility trial
Source: BMJ Open. 2023 Aug 22;13(8):e066609. doi: 10.1136/bmjopen-2022-066609 (PMC10445364; doi:10.1136/bmjopen-2022-066609)
Supplement: Supplementary data [file bmjopen-2022-066609supp009.pdf]

Supplementary Table 2- Average predictions for main outcomes at main time points

Table 2: Average predictions for main outcome values at the main time points during the trial, i.e. baseline, 2 months, 8 months and *changes from the baseline* at 2 months, 8 months and by the time they reach their max/min values. This set of predictions are made across intervention and ILD clinical groups, adjusting for age (hence predictions are made for patients of average age of 71 years). Estimated peak/saddle times are also displayed.

Supplementary Table 2- Average predictions for main outcomes at main time points

Outcome	Time	Control (MICT)				Intervention (HIIT)			
		Fibrosis		CTD-ILD		Fibrosis		CTD-ILD	
		Mean (95% CI) predicted value	Change from baseline	Mean (95% CI) predicted value	Change from baseline	Mean (95% CI) predicted value	Change from baseline	Mean (95% CI) predicted value	Change from baseline
6MWD	Baseline	415.84 (360.8 to 470.9))		393.89 (337.8 to 450.0)		367.94 (327.4 to 408.5)		358.88 (304.8 to 413.0)	
	2 months	464.63 (409.4 to 519.84)	45.5 (17.5 to 73.5)	442.92 (387.3 to 498.6)	55.4 (35.3 to 75.6)	401.22 (360.1 to 442.3)	28.9 (5.7 to 52.0)	410.63 (356.3 to 465.0)	38.3 (14.2 to 63.5)
	8 months	459.03 (398.5 to 519.6)	39.2 (11.2 to 67.2)	438.02 (381.6 to 494.5)	49.2 (28.8 to 69.6)	349.07 (304.9 to 393.3)	- 6.84 (-30.9 to 17.3)	413.93 (351.5 to 476.4)	3.1 (-23.5 to 29.7)
	Peak value	490.40 (432.7 to 548.1)	42.5 (15.2 to 69.8)	469.02 (412.6 to 525.5)	52.4 (33.1 to 71.8)	409.60 (366.8 to 452.4)	19.2 (-3.3 to 41.7)	440.80 (382.9 to 498.7)	20.4 (-4.2 to 45.1)
	Peak time-months	4.85 (4.1 to 5.6)		4.87 (4.4 to 5.4)		3.63 (3.1 to 4.2)		5.09 (4.3 to 5.9)	
SNIP	Baseline	99.94 (86.3 to 113.5)		90.69 (77.4 to 104.0)		96.92 (86.5 to 107.3)		77.55 (64.5 to 90.6)	
	2 months	106.89 (93.4 to 120.4)	14.3 (4.0 to 24.5)	100.33 (87.2 to 113.4)	10.9 (3.7 to 18.0)	102.38 (91.6 to 113.1)	8.6 (0.2 to 16.9)	85.46 (72.4 to 98.6)	5.2 (-3.9 to 14.2)
	8 months	97.52 (79.9 to 115.2)	5.8 (-5.3 to 16.9)	99.04 (84.9 to 113.4)	2.4 (-5.6 to 10.5)	88.54 (75.3 to 101.8)	0.1 (-9.4 to 9.7)	78.98 (62.5 to 95.5)	-3.3 (-13.2 to 6.6)
	Peak value	108.84 (94.3 to 123.4)	11.8 (1.8 to 21.8)	105.37 (91.6 to 119.2)	9.2 (2.4 to 16.0)	103.23 (91.7 to 114.8)	4.6 (-3.6 to 12.6)	88.35 (73.9 to 102.7)	2.2 (-6.6 to 10.9)
	Peak time-months	3.76 (2.1 to 5.4)		4.83 (3.8 to 5.9)		3.17 (1.9 to 4.5)		4.14 (2.8 to 5.5)	
QUADS DOM	Baseline	18.13 (15.4 to 20.9)		19.68 (16.9 to 22.4)		17.66 (15.6 to 19.7)		17.71 (15.1 to 20.3)	
	2 months	21.20 (18.4 to 24.0)	4.6 (2.6 to 6.7)	22.10 (19.4 to 24.8)	2.7 (1.2 to 4.2)	20.09 (17.9 to 22.3)	2.8 (1.2 to 4.3)	20.25 (17.6 to 22.9)	2.4 (0.4 to 4.4)
	8 months	23.64 (19.7 to 27.6)	5.4 (3.2 to 7.7)	22.59 (19.6 to 25.6)	3.5 (1.8 to 5.1)	20.62 (17.9 to 23.4)	3.5 (1.9 to 5.2)	21.10 (17.5 to 24.7)	3.2 (1.0 to 5.3)
	Peak value	23.99 (20.4 to 27.5)	5.2 (3.1 to 7.3)	23.63 (20.7 to 26.5)	3.1 (1.7 to 4.5)	21.64 (19.1 to 24.2)	3.2 (1.8 to 4.6)	21.98 (18.8 to 25.2)	2.9 (0.9 to 4.8)

Supplementary Table 2- Average predictions for main outcomes at main time points

	**Peak time-months**	6.44 (4.2 to 8.7)		5.29 (4.0 to 6.6)		5.31 (4.0 to 6.6)		5.50 (3.8 to 7.2)	
**SGRQ-I**	**Baseline**	44.01 (33.2 to 54.9)		38.42 (27.6 to 49.2)		55.00 (47.0 to 63.0)		37.75 (27.6 to 47.9)	
	**2 months**	37.36 (26.4 to 48.3)	- 8.4 (-16.1 to -0.7)	32.11 (21.5 to 42.7)	-6.6 (-12.0 to -1.2)	49.72 (41.5 to 58.0)	-3.5 (-9.6 to 2.5)	31.38 (21.2 to 41.6)	- 6.5 (-13.9 to 0.9)
	**8 months**	40.39 (27.4 to 53.4)	3.1 (- 10.9 to 4.7)	36.18 (25.2 to 47.2)	-1.3 (-6.7 to 4.2)	56.87 (47.6 to 66.1)	1.8 (-4.3 to 7.8)	35.28 (23.2 to 47.4)	-1.2 (-8.7 to 6.35)
	**Min value**	34.43 (22.6 to 46.3)	-6.2 (-13.7 to 1.3)	29.59 (18.6 to 40.6)	- 4.6 (-9.7 to 0.5)	48.25 (39.3 to 57.2)	- 1.9 (-7.8 to 3.8)	28.80 (17.8 to 39.8)	-4.4 (-11.7 to 2.8)
	**Min time-months**	4.47 (3.2 to 5.8)		4.29 (3.5 to 5.1)		3.76 (2.9 to 4.7)		4.32 (3.2 to 5.5)	

Data are mean (95% CI)
