# Supplementary material for: Comparison of high-intensity interval training versus moderate-intensity continuous training in pulmonary rehabilitation for interstitial lung disease: a randomised controlled pilot feasibility trial
Source: BMJ Open. 2023 Aug 22;13(8):e066609. doi: 10.1136/bmjopen-2022-066609 (PMC10445364; doi:10.1136/bmjopen-2022-066609)
Supplement: Supplementary data [file bmjopen-2022-066609supp005.pdf]

Supplementary Figure 2- Random effects models for 6MWD

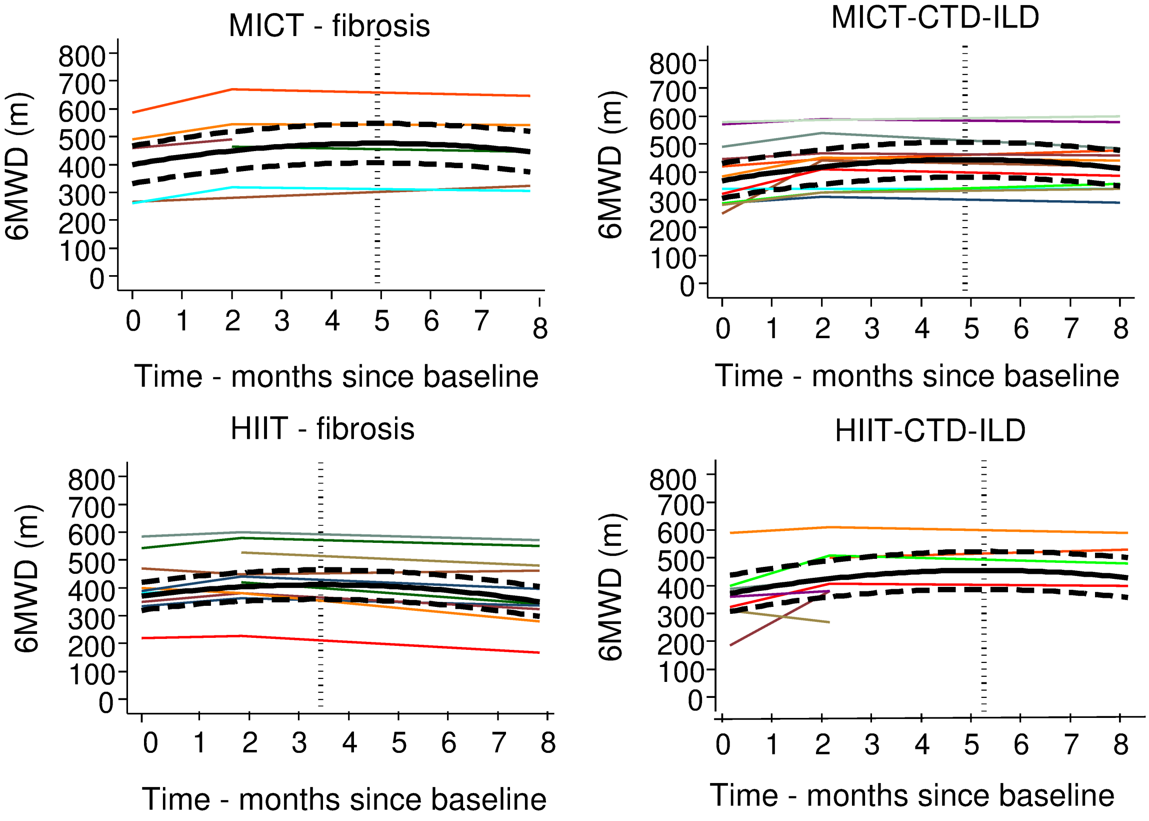

Figure 2: Random effects models for the 6MWD by intervention (HIIT vs MICT) and ILD clinical subgroup (Fibrosis vs CTD-ILD)
