# Supplementary material for: Comparison of high-intensity interval training versus moderate-intensity continuous training in pulmonary rehabilitation for interstitial lung disease: a randomised controlled pilot feasibility trial
Source: BMJ Open. 2023 Aug 22;13(8):e066609. doi: 10.1136/bmjopen-2022-066609 (PMC10445364; doi:10.1136/bmjopen-2022-066609)
Supplement: Supplementary data [file bmjopen-2022-066609supp003.pdf]

Supplementary File 3-Additional analysis on the effect of DLCO on key outcomes

Post-hoc statistical analysis on the effect of baseline DLCO on key outcomes

We have investigated DLCO in a number of different ways.

1. Baseline assessment, i.e. DLCO by intervention and clinical groups simultaneously.

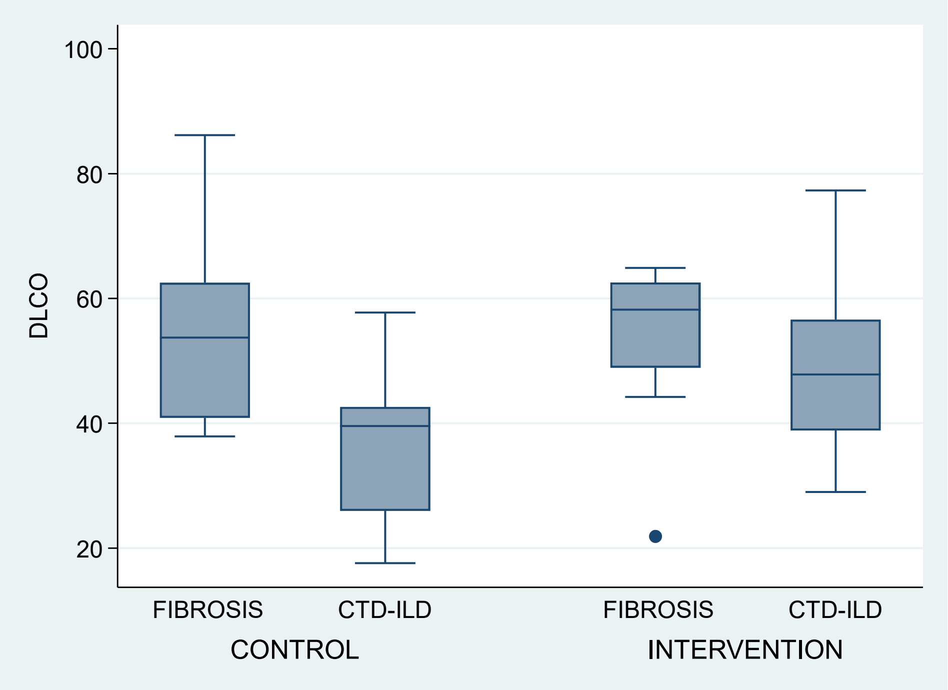

Based on these data, the evidence suggests that DLCO differs by intervention group (adjusted p value=0.003)– finding initially deems as random randomization imbalance. Nevertheless, there is not enough evidence to suggest that DCLO levels differ by clinical group, i.e. the DCLO difference between the clinical groups seems to remain similar with the intervention group (p=0.069).

2. Relationship between the 4 outcomes and DLCO at the baseline. The results are presented in Table 1 below. There was no evidence for an interaction of DLCO with either intervention or clinical group regarding these outcomes. Only coefficients corresponding to DLCO are shown for a succinct message, but they are adjusted for intervention groups and clinical groups.

Table 1: Relationship between the 4 key outcomes and baseline DLCO, adjusted for intervention and clinical groups.

	Estimate	SE	T value	p-value	95%CI low	95%CI high	No Obs	Association
MWT	.9787011	1.178773	0.83	0.410	-1.39138	3.348783	50	UNADJUSTED
SNIP	.1441871	.2431136	0.59	0.556	-.3454688	.633843	47	
QUADS DOM	-.0956757	.0499201	-1.92	**0.061**	-.1958477	.0044964	54	
SGRQI	-.0614169	.1979514	-0.31	0.758	-.4586354	.3358016	54	
MWT	8799743	1.31952	0.67	0.508	-1.774558	3.534506	50	ADJUSTED FOR
SNIP	.0873187	.2717802	0.32	0.750	-.4604183	.6350556	47	INTERVENTION
QUADS DOM	-.123836	.0548133	-2.26	**0.028**	-.2338782	-.0137938	54	GROUP

Supplementary File 3-Additional analysis on the effect of DLCO on key outcomes

SGRQI	.0017764	.2204537	0.01	0.994	-.4408028	.4443556	54	
MWT	1.047737	1.366581	0.77	0.447	-1.703048	3.798522	50	ADJUSTED FOR
SNIP	.1784858	.2751485	0.65	0.520	-.3764041	.7333758	47	INTERVENTION
QUADS DOM	-.1468699	.0553319	-2.65	**0.011**	-.2580074	-.0357325	54	AND CLINICAL
SGRQI	.0816139	.2234533	0.37	0.716	-.3672054	.5304331	54	GROUP
MWT	1.078732	1.302553	0.83	0.412	-1.544745	3.702209	50	ADJUSTED FOR
SNIP	.181307	.2570293	0.71	0.484	-.337399	.7000131	47	INTERVENTION,
QUADS DOM	-.1478509	.0529411	-2.79	**0.007**	-.25424	-.0414617	54	CLINICAL
SGRQI	.0764789	.2163264	0.35	0.725	-.3582453	.5112032	54	GROUP AND AGE

MWT=distance in the 6MWT, SNIP=Sniff nasal inspiratory pressure, QUADS DOM=Quadriceps strength in the dominant leg and SGRQ-I=total score in the St George's Respiratory Questionnaire for ILD

There is some evidence to suggest that, at baseline, the DLCO is negatively associated with QUADS DOM but, based on these data, none of the other important measurements seemed to have been affected.

3. However, when analysing the data longitudinally, after adjusting for time and its quadratic term, interactions between time and intervention and clinical groups and age, the effect on the QUADS DOM seem to weaken. Below, we additionally adjust the models presented in Table 1 by DLCO – after investigating its interactions with intervention/clinical groups too. Only coefficients corresponding to DLCO are shown for a succinct message, but they are adjusted for time, intervention groups and clinical groups, age and their interactions.

Table 2: Relationship between DLCO and key outcomes, adjusted for time, intervention groups and clinical groups, age and their interactions.

	Estimate	SE	T value	p-value	95%CI low	95%CI high	No Obs	Association
MWT	1.336415	.9914012	1.35	0.178	-.6066961	3.279525	113/53	ADJUSTED FOR
SNIP	.3109333	.2361878	1.32	0.188	-.1519863	.7738529	113/53	INTERVENTION,
QUADS DOM	-.0910743	.0473067	-1.93	**0.054**	-.1837937	.0016452	119/55	CLINICAL
SGRQI	.1404341	.197889	0.71	0.478	-.2474212	.5282893	120/55	GROUP AND AGE

MWT=distance in the 6MWT, SNIP=Sniff nasal inspiratory pressure, QUADS DOM=Quadriceps strength in the dominant leg and SGRQ-I=total score in the St George's Respiratory Questionnaire for ILD

This is not to say that the effect on these outcomes may not be important, we just did not capture it based on these data.
