# Supplementary material for: Comparison of high-intensity interval training versus moderate-intensity continuous training in pulmonary rehabilitation for interstitial lung disease: a randomised controlled pilot feasibility trial
Source: BMJ Open. 2023 Aug 22;13(8):e066609. doi: 10.1136/bmjopen-2022-066609 (PMC10445364; doi:10.1136/bmjopen-2022-066609)
Supplement: Author's manuscript [file bmjopen-2022-066609.draft_revisions.pdf]

BMJ Open

BMJ Open is committed to open peer review. As part of this commitment we make the peer review history of every article we publish publicly available.

When an article is published we post the peer reviewers' comments and the authors' responses online. We also post the versions of the paper that were used during peer review. These are the versions that the peer review comments apply to.

The versions of the paper that follow are the versions that were submitted during the peer review process. They are not the versions of record or the final published versions. They should not be cited or distributed as the published version of this manuscript.

BMJ Open is an open access journal and the full, final, typeset and author-corrected version of record of the manuscript is available on our site with no access controls, subscription charges or pay-per-view fees (http://bmjopen.bmj.com).

If you have any questions on BMJ Open's open peer review process please email info.bmjopen@bmj.com

BMJ Open

Comparison of high intensity interval training versus moderate intensity continuous training in pulmonary rehabilitation for interstitial lung disease; a randomised controlled pilot feasibility trial.

Journal:	*BMJ Open*
Manuscript ID	bmjopen-2022-066609
Article Type:	Original research
Date Submitted by the Author:	15-Jul-2022

Complete List of Authors:	Nikoletou, Dimitra; St George's University of London, Centre for Allied Health; Kingston University, Rehabilitation sciences<br>Chis Ster, Irina; St George's University of London, Infection and Immunity Research Institute<br>Lech, Carmen; St George's University of London, Infection and Immunity Research Institute<br>MacNaughton, Iain; St George's University of London, Infection and Immunity Research institute<br>Chua, Felix; Royal Brompton and Harefield NHS Foundation Trust, Interstitial Lung Disease Unit; Imperial College London, National Heart and Lung Institute<br>Aul, Raminder; St George's University Hospitals NHS Foundation Trust, Respiratory Medicine<br>Jones, Paul; St George's University of London, Infection and Immunity Research institute

Keywords:	Interstitial lung disease < THORACIC MEDICINE, REHABILITATION MEDICINE, PREVENTIVE MEDICINE

SCHOLARONE™
Manuscripts

I, the Submitting Author has the right to grant and does grant on behalf of all authors of the Work (as defined in the below author licence), an exclusive licence and/or a non-exclusive licence for contributions from authors who are: i) UK Crown employees; ii) where BMJ has agreed a CC-BY licence shall apply, and/or iii) in accordance with the terms applicable for US Federal Government officers or employees acting as part of their official duties; on a worldwide, perpetual, irrevocable, royalty-free basis to BMJ Publishing Group Ltd ("BMJ") its licensees and where the relevant Journal is co-owned by BMJ to the co-owners of the Journal, to publish the Work in this journal and any other BMJ products and to exploit all rights, as set out in our licence.

The Submitting Author accepts and understands that any supply made under these terms is made by BMJ to the Submitting Author unless you are acting as an employee on behalf of your employer or a postgraduate student of an affiliated institution which is paying any applicable article publishing charge ("APC") for Open Access articles. Where the Submitting Author wishes to make the Work available on an Open Access basis (and intends to pay the relevant APC), the terms of reuse of such Open Access shall be governed by a Creative Commons licence – details of these licences and which Creative Commons licence will apply to this Work are set out in our licence referred to above.

Other than as permitted in any relevant BMJ Author's Self Archiving Policies, I confirm this Work has not been accepted for publication elsewhere, is not being considered for publication elsewhere and does not duplicate material already published. I confirm all authors consent to publication of this Work and authorise the granting of this licence.

For peer review only

Title: Comparison of high intensity interval training versus moderate intensity continuous training in pulmonary rehabilitation for interstitial lung disease; a randomised controlled pilot feasibility trial.

Authors:

Dr Dimitra Nikoletou[1, 2,] PhD dinikole@sgul.ac.uk

Dr Irina Chis-Ster[3], PhD ichisste@sgul.ac.uk

Ms Carmen Y. Lech[3], BSc carmenyanina.lech@gmail.com

Mr Iain S. MacNaughton[3], BSc iainmacn62@gmail.com

Dr Felix Chua MD[4], PhD F.Chua@rbht.nhs.uk

Dr Raminder Aul MD[5] raminder.aul@stgeorges.nhs.uk

Professor Paul Jones[3] PhD pjones@sgul.ac.uk

Authors' affiliations:

1. Centre for Allied Health, St George's University of London, Cranmer Terrace, London, SW17 ORE, UK.
2. Faculty of Health, Science, Social care and Education, Kingston University, London, UK.
3. Infection and Immunity Research Institute, St George's University of London, Cranmer Terrace, London, SW17 ORE, UK
4. Department of Respiratory Medicine, Royal Brompton & Harefield NHS Foundation Trust, London, UK.
5. Department of Respiratory Medicine, St George's Hospital NHS Foundation Trust, London SW17 ORE, UK

Corresponding author:
Dr Dimitra Nikoletou, Centre for Allied Health, St George's University of London, Cranmer Terrace, London, SW17 ORE and (honorary) Faculty of Health, Science, Social care and Education, Kingston University, London, UK.
Tel: 0208 725 2277 Email: dinikole@sgul.ac.uk

Key words: Interstitial lung disease, ILD, Pulmonary rehabilitation, Interstitial pulmonary fibrosis, exercise, high intensity interval training, HIIT.

Running head: Feasibility of HIIT versus MICT in ILD PR programmes

Trial registration: This trial was registered in ISRCTN, with the ID number ISRCTN55846300.

Word count: 3,759

For peer review only

Abstract

Objectives: This study aimed to investigate the feasibility and efficacy of high intensity interval training (HIIT) compared to moderate intensity continuous training (MICT) in pulmonary rehabilitation (PR) for people with interstitial lung disease (ILD).

Design: Single-centre, randomised controlled feasibility, pilot trial.

Setting: Patients were recruited from the chest clinic of a tertiary centre for ILD and attended circuit-based PR sessions in the hospital's gym. On completion, patients commenced a personalised 6-month community programme.

Participants: 58 patients, stratified per ILD type, were randomised into two groups. 33 patients (M:F) (age SD) were randomised to the HIIT exercise mode and 25 to the MICT mode (M:F), (age SD).

Interventions: 8-week, twice weekly, circuit-based PR programme of exercise and education, followed by a personalised 6-month home exercise programme.

Outcome measures: Feasibility outcomes included staff-to-patient ratio and dropout rates per group. Primary outcome was the 6-minute walk distance (6MWD). Secondary outcomes included the sniff nasal pressure, mouth inspiratory and expiratory pressures, handgrip and quadriceps strength and health status. Random-effects models were used to evaluate average variation in outcomes through time across the two groups.

Results: The 6MWD peaked earlier with HIIT compared to MICT (at 4 months versus 5 months) but values were lower at peak (mean (95%CI) 26.3m (3.5, 49.1) versus 51.6m (29.2, 73.9) and declined faster at 6-months post PR. Secondary outcomes showed similar faster but smaller improvements with HIIT over MICT and more consistent maintenance 6-months post-PR with MICT than HIIT.

Conclusions: HIIT is feasible in circuit-based ILD PR programmes and provides quick improvements but requires closer supervision of training and resources than MICT and benefits may be less well sustained. This would make it a less attractive option for clinical PR programmes. A definitive, multi-centre randomised controlled trial is required to definitively address the role of HIIT in ILD.

For peer review only

Strengths and Limitations of this study

- This randomised controlled feasibility, pilot study, showed that HIIT is feasible in circuit-based PR programmes.
- This study adds valuable information for the design of future, definitive studies, on the peak and subsequent deterioration of outcomes over time depending on exercise mode.
- Our results identify the point at which exercise reinforcement may be needed after completion of a HIIT programme.
- Although it was possible to recruit enough ILD patients from a single site, not enough IPF patients could be recruited to evaluate the effect of HIIT on this particular group.
- Despite stratification during the randomisation process, the DLCO was lower in the HIIT group compared to the MICT group.
- We did not use supplementary oxygen during PR for patients other than those already on long-term oxygen therapy.

For peer review only

Background

Interstitial lung disease (ILD) is a broad term used to describe a diverse group of pulmonary conditions that cause fibrotic changes in the lung interstitium with subsequent reduction in lung compliance and oxygen diffusion capacity [1, 2]. ILD is categorised into its different types according to clinical, radiological and pathophysiological changes and multidimensional indexes have been created to predict mortality risk [3, 4]. The clinical presentation includes breathlessness on exertion, dry and persistent cough, fatigue, progressive exercise limitation and respiratory failure at the very severe stages. [5]

Pulmonary rehabilitation (PR) is a well-established rehabilitation option for various respiratory conditions [5, 6] but in ILD patients the optimal training programme, especially for longer term benefits, remains unclear. [5, 7] Recent evidence suggests that patients with some types of ILD, such as asbestosis and idiopathic pulmonary fibrosis (IPF), may receive greater benefit from PR than others and effects may be more lasting in patients with milder disease [8]. However, while the traditional approach to prescribing rehabilitation in PR is moderate intensity continuous training (MICT), other training modalities may offer greater benefits.

High intensity interval training (HIIT) is an exercise modality characterised by intervals of high intensity exercise followed by intervals of low intensity or rest [9]. There is growing evidence that HIIT improves a broad range of cardiovascular risk factors, including insulin sensitivity and VO_2peak [10], and its safety has been evaluated in patients with cardiovascular and metabolic disease [10-13]. In patients with chronic obstructive pulmonary disease (COPD) HIIT has been used in severe patients who cannot maintain exercise due to breathlessness [14-16] although its overall superiority over MICT has been questioned in this patient group [17]. HIIT has been used safely in ILD patients preparing for lung transplantation [18] but its effects have yet to be evaluated in the context of an ILD-tailored PR programme.

For peer review only - http://bmjopen.bmj.com/site/about/guidelines.xhtml

For peer review only

The aim of this pilot study was therefore to evaluate: a) the feasibility of using HIIT in an ILD-PR programme, b) determine the short and medium-term effects of HIIT on exercise capacity, respiratory and peripheral muscle strength, breathlessness and health status and c) explore responses in different types of ILD. Some of the results of this study have been reported previously abstract form [19].

Methods

Study design

Single-blind, randomised-controlled feasibility, pilot trial.

Study population

Symptomatic patients over 18 years old with a respiratory-physician diagnosis of ILD, of all types and severity apart from sarcoidosis, were invited to participate. This included patients with idiopathic pulmonary fibrosis (diagnostic criteria consistent with the International Consensus statement [1]), connective tissue disease related ILD, non-specific interstitial pneumonia, usual interstitial pneumonia, and chronic hypersensitivity pneumonitis. Patients were eligible to participate if they were ambulant and had symptoms of dyspnoea on exertion. Exclusion criteria were a history of syncope on exertion, patients with sarcoidosis and severe co-morbidities (such as musculoskeletal, neurological or cardiovascular problems) that would interfere with their ability to exercise.

All participants provided written informed consent. The trial protocol was pre-registered with the ISRCTN registry (ISRCTN55846300) and approved by the South East Coast-Surrey Ethics committee (14/LO/0149).

Patient and Public involvement

People with ILD were involved in the original design of this research and determined acceptability of the proposed interventions and likelihood of adherence to this programme. During the study, all participants

For peer review only - http://bmjopen.bmj.com/site/about/guidelines.xhtml

For peer review only

were involved in the conduct of the research by giving regular feedback in the education-discussion part of the PR programme and via interviews. On completion of this study, some participants formed a local ILD-Support Group and viewed the final results for dissemination.

Randomisation and blinding

Following baseline assessments, participants were randomised into 2 groups: exercise using moderate intensity continuous training (MICT group) or exercise using high-intensity interval training (HIIT group). Participants were stratified by ILD type to ensure a balanced distribution of types between groups. They were placed into 3 sub-groups: a) Idiopathic group (e.g. IPF); b) Autoimmune group (CTD-ILD) and c) Chronic Hypersensitivity pneumonitis group (CHP/EAA). The investigator performing all assessments and the statistician were blinded throughout the study.

For peer review only

Intervention

The ILD-PR programme

This was an 8-week, twice weekly outpatient programme, including only people with ILD, which consisted of circuit-based training, supervised by two experienced physiotherapists. The programme consisted of an hour of exercise and an hour of education. Exercise was set, monitored and progressed according to a standardised protocol for each group allocation. Aerobic training intensity at the MICT group was set at 60% maximum heart rate (HRmax) and progressed each week. In the HIIT group, intensity was set at 80% HRmax and patients were instructed to exercise in dynamic intervals followed by low intensity exercise or rest. The aim in this group was to increase the amount of time on the 'high intensity' phase each week. The average time on HIIT was 2.5 min in the first week and recovery time varied depending on type and severity of ILD. Progression of training was determined by reducing time in the low intensity phase of HIIT and/or increasing

For peer review only - http://bmjopen.bmj.com/site/about/guidelines.xhtml

time in the target HR. Each participant's individualised programme was recorded on exercise logs during the sessions.

Endurance exercise in each session included treadmill walking, brisk walking between 2 cones, cycle ergometer, mini-trampoline and step ups. A minimum of 30 minutes were dedicated to aerobic training.

Strength training exercises, upper and lower limb, were conducted using 'Thera-bands' and functional movements such as sit-to-stand and ball raises.

Heart rate and oxygen saturation were monitored using pulse oximeters and perceived exertion and breathlessness were recorded at set intervals using the modified 10-point Borg scale and the Rate of Perceived Exertion scale (RPE) respectively [20, 21].

The education session, on the 2nd hour of the programme was common for all participants and included presentations and discussion on topics important to ILD patients.

On the first day of the programme all participants were given an 'ILD-Pulmonary rehabilitation booklet' designed by our team, which contained information and pictures of all exercises, an exercise diary and summary of the education topics. All participants were encouraged to continue exercising at home on the days they did not attend the programme.

The 6-month programme

Following completion of the PR programme, participants commenced a personalised community exercise programme, starting at the level they achieved at the end of PR. The 6-month programme included outdoor walking in HIIT or MICT mode, as well as home-based stretching and strength training exercises. Progress was monitored by phone calls (once per month) and at least one home visit by the research physiotherapists on the 3rd month. The physiotherapists guided participants on how to fill in the exercise diaries and progress training depending on mode of exercise. The ILD-PR booklet was used as a resource for the home exercises and as a reminder of the educational topics.

For peer review only - http://bmjopen.bmj.com/site/about/guidelines.xhtml

Outcomes

All assessments were performed by the same investigator (DN), using identical instructions and methods, at three time points; at baseline, at 8-weeks (post-PR) and at 6 months (post-community programme). The investigator was blinded throughout the study.

Feasibility outcomes included the proportion of eligible patients who consented to the study, attrition in each group, staff-to-patient ratio and supervision requirements, reasons for discontinuing the programme and adverse events.

The primary efficacy outcome was functional exercise capacity and was measured using the 6-minute walk test distance (6MWD) with standardised instructions as per ATS guidelines [22].

Secondary outcomes were: a) Respiratory muscle function- Sniff nasal pressure (SNIP) and the maximal inspiratory and expiratory pressures (PImax and PEmax) were recorded using a handheld portable respiratory muscle testing device (MicroRPM, CareFusion, Basingstoke, UK). A minimum of 10 attempts were made for each test, on at least one session. PImax was recorded from functional residual capacity (FRC) and PEmax from total lung capacity (TLC).

b) Peripheral muscle function- Handgrip strength, assessed in the dominant hand, with the elbow at 90 degrees angle, using a hydraulic hand dynamometer (JAMAR Hydraulic Hand Dynamometer, Homecraft Rolyan Ltd, Nottinghamshire, UK). Quadriceps strength (QUADS-DOM) and hip flexion (HIP-DOM) were assessed in sitting position, with the knee at 90 degrees angle and back upright and supported using a hand-held dynamometer (Lafayette Manual Muscle Tester Model 01163, Lafayette Instruments, Lafayette, IN, USA).

c) Health status, using the St George's Respiratory Questionnaire for IPF (SGRQ-I) [23] and the Hospital Anxiety and Depression scale (HAD) [24].

For peer review only - http://bmjopen.bmj.com/site/about/guidelines.xhtml

For peer review only

Spirometry data and anthropometric measurements (height, weight, %fat, waist, hip and neck circumference) were also collected during assessment sessions.

Data were collected and managed using REDCap electronic data capture tools hosted at the NHS Trust (1). REDCap (Research Electronic Data Capture) is a secure, web-based application designed to support data capture for research studies providing an intuitive interface for validated data entry, audit trails for tracking data manipulation, automated export procedures for seamless data downloads to common statistical packages and procedures for importing data from external sources.

Statistical analysis

This was a pilot study to test feasibility and inform the design and power of a larger, definitive, randomised controlled trial. An intention to treat analysis was used. An indicative sample size calculation was based on previous studies aiming to detect between-group changes of 38± 43m with an 80% power at $p < .05$ significance level in the 6MWD following rehabilitation (25-27).

Random effects models were used to estimate average values in outcomes and predictions were derived following a nonlinear, quadratic model for changes over time and a 3-way interaction between time, intervention (HIIT vs MICT) and clinical group (ILD type) as well as between age, intervention and clinical group. For each of the 4 key outcomes of interest (6MWD, SNIP, SGRQ-I and Quads strength), a series of nested models of increasing complexity was developed and tested for their statistical significance against the next less complex model. The average variation of outcomes through time, within and between-groups, are presented as means (95% CI) and descriptive statistics as mean (SD). For the plots of change over time, patients were grouped into tertiles. All analyses were conducted on STATA software (version 11).

For peer review only - http://bmjopen.bmj.com/site/about/guidelines.xhtml

Results

Patient flow

60 participants consented to the study but two participants withdrew prior to commencement of the PR programme. A total of 58 started the PR programme. Figure 1 is the CONSORT diagram showing participant flow.

Figure 1 around here

Baseline characteristics are shown in Table 1 . There were no differences between groups at baseline, except for DLco which was significantly lower in the HIIT group.

Table 1: Baseline characteristics

Variable	Total Group (n= 58)	Control group (MICT) (n=25)	Intervention group (HIIT) (n=33)	Between group P value
**Age (years)**	70.0 (11.05)	69.8 (10.8)	70.5 (10.9)	0.89
**Gender (male), (number (%))**	32 (55.2)	14 (56)	18 (55)	0.91
**ILD Type, (no. (%))**				0.10
• IPF	18 (31.0)	8 (32)	10 (30.3)	
• CTD-ILD	28 (48.3)	15 (60)	13 (39.4)	
• CHP	12 (20.7)	2 (8)	10 (30.3)	
**ILD-GAP index,** (number (%))				0.72§
• 0-1	24 (41.4)	12 (48)	12 (48)	
• 2-3	20 (34.5)	9 (36)	11 (33.3)	
• 4-5	10 (17.2)	4 (16)	6 (18.2)	
• >5	2 (3.4)	0 (0)	2 (6.1)	
**BMI (kg/m²)**	27.3 (4.6)	27.5 (3.8)	27.2 (5.1)	0.84
**Waist/Hip ratio**	0.9 (0.1)	0.9 (0.1)	0.9 (0.1)	0.60

For peer review only - http://bmjopen.bmj.com/site/about/guidelines.xhtml

**Fat (%)**	29.9 (8.6)	29.9 (9.3)	30.1 (8.1)	0.95
**FVC (%,predicted)**	79.1 (21.8)	85.8 (24.0)	73.8 (18.8)	0.11
**DLCO (%, predicted)**	47.0 (14.7)	53.9 (13.1)	41.7 (13.9)	0.002
**Comorbidities (no. (%))**				
• Cardiac	21 (36.2)	8 (32)	13 (39.4)	0.56
• Hypertension	23 (39.7)	9 (36)	14 (42.4)	0.62
• Diabetes	15 (25.9)	7 (28)	8 (24.2)	0.75
• High Cholesterol	12 (20.7)	7 (28)	5 (15.2)	0.23
**Prednisolone medication (no. (%))**				0.34§
• < 5mg	19 (32.8)	9 (36)	10 (30.3)	
• 5-10 mg	19 (32.8)	5 (20)	14 (42.4)	
• >10 mg	3 (5.17)	2 (8)	1 (3)	
**Smoking status (no. (%))**				0.31§
• Current smoker	13 (22.4)	5 (20)	8 (24.2)	
• Former smoker	9 (15.5)	6 (24)	3 (9.1)	
• Never smoked	33 (56.9)	13 (52)	20 (60.6)	
• Missing records	3	1	2	
**Pack year history**	8.9 (15.9)	10.7 (16.5)	7.4 (15.6)	0.50
Median/IQR interval	0(0, 10)	0(0, 20)	0(0, 4.5)	
Missing	7	3	5	
**6MWD (m)**	371.7 (122.2)	380.8 (139.8)	364.6 (108.3)	0.65
6MWD Missing	6	2	4	
**SpO_2 in room air (%)**	96 (1.5)	96.5 (1.8)	96 (1.5)	0.3
Missing	6	2	4	
**No. on LTOT**	3	1	2	n/a
**SNIP (cmH_2O)**	94.8 (24.3)	96.8 (25.9)	93.1 (24.5)	0.61
Missing	9	3	6	
**PImax (cmH_2O)**	91.5 (30.4)	91.1 (34.2)	91.8 (27.4)	

For peer review only - http://bmjopen.bmj.com/site/about/guidelines.xhtml

Missing	8	2	6	
**PEmax (cmH_2O)**	106.6 (32.0)	114.4 (40.3)	100.7 (23.5)	0.22
Missing	23	10	13	
**Handgrip (dominant hand) (kg)**	25.8 (10.2)	27.2 (11.2)	24.8 (9.4)	0.42
Handgrip Missing	4	2	2	
**Quads extension (dominant side) (kg)**	18.3 (5.5)	18.7 (6.2)	18 (5.0)	0.65
Missing	2	0	2	
**Hip flexion (dominant side) (kg)**	15.6 (4.6)	15.3 (5.1)	15.9 (4.3)	0.41
Missing	1	0	1	
**HAD-A**	6.3 (4.2)	6.2 (4.6)	6.3 (3.9)	0.88
Missing	1	0	1	
**HAD-D**	5.5 (3.7)	5.8 (3.2)	5.2 (4.0)	0.28
Missing	1	0	1	
**SGRQ-I Total score**	46.6 (21.2)	43.3 (20.3)	49.3 (21.8)	0.29
Missing	1	0	1	

Data are means (SD) unless otherwise stated. The p-value tests the general null hypothesis of similarity between the randomized groups. The methods include t-tests (equal or unequal variances), Kruskal- Wallis and chi-squared (Fisher exact) as appropriate according to the nature of the variables. All tests have been carried out on the complete data only and missing data were not considered as a separate category. §= p value was calculated considering the whole (balanced) distribution.

Feasibility of HIIT compared to MICT

HIIT was well tolerated in a circuit-based clinical PR programme but required closer supervision to monitor participants' target heart rate and avoid desaturation below 85%. There were no adverse events from HIIT during or after the PR programme although attrition in the HIIT group was higher (42% compared to 28% in the MICT group) this did not appear to be due to a specific factor that could be directly attributed to the type of programme (Figure 1).

For peer review only - http://bmjopen.bmj.com/site/about/guidelines.xhtml

For peer review only

Outcome	Time	Group data Mean (95% CI)		Change from baseline Mean (95% CI)		Change from baseline adjusted for age Mean (95% CI)	
		**Control (MICT)**	**Intervention (HIIT)**	**Control (MICT)**	**Intervention (HIIT)**	**Control (MICT)**	**Intervention (HIIT)**
**6MWD (m)**	**Baseline**	382 (336, 429)	372 (331, 412)				
	**2 months**	433 (386 480)	411 (370, 453)	54.7 (32., 76.9)	38.6 (15.95, 61.17)	53.2 (33.66, 72.70)	36.3 (16.42, 56.06)
	**8 months**	428 (378, 476)	374 (331, 418)	48.2 (24.0, 72.4)	2.64 (-23.4, 28.6)	46.6 (26.5, 66.7)	.06 (-21.95, 22.08)
	**Peak value**	460 (411, 508)	425 (382, 468)	51.6 (29.2, 73.9)	26.29 (3.5, 49.1)	50.02 (31.2, 68.8)	23.88 (4.5, 43.3)

Change in exercise capacity, respiratory and peripheral muscle strength and health status

The change in outcomes from baseline at all assessment points are shown in Figure 2 and suggest a non-linear trend. This was then tested using linear and non-linear models and it was found that a quadratic model provided a significantly better fit than a linear model. The 6MWD increased over time from baseline in both groups, after which it tended to return towards starting levels ($p<0.001$ for a quadratic term of change over time), Figure 3. The p value for the group-time interaction term was $p=0.006$ suggesting that the trajectories differed between groups. The HIIT group peaked quicker, around 4 months compared to around 5 months with MICT (Figure 3). The models also suggested that the change in 6MWD values were smaller with HIIT (Table 2, Figure 2); mean change at peak was 51.6m (95% CI 29.2, 73.9) in the MICT group compared to 26.3m (3.5, 49.1) with HIIT. The picture was the same using the estimates at 2 and 8 months (Table 2, Figure 2). Age was inversely associated with the average 6MWD values in both groups, but the time course was similar across ages (Fig. 3).

Figure 2 and 3 around here

Table 2: Average predictions for main outcomes values at the main time points during the trial and for changes from baseline (actual and adjusted for age)

For peer review only - http://bmjopen.bmj.com/site/about/guidelines.xhtml

	**Peak time-months**	4.87<br>(4.43, 5.30)	4.05<br>(3.62, 4.48)				
**SNIP (cmH2O)**	**Baseline**	95<br>(85, 105)	92<br>(82, 101)				
	**2 months**	104<br>(94, 115)	98<br>(88, 108	9.53<br>(2.02, 17.04)	10.07<br>(2.28, 17.85)	9.21<br>(1.98, 16.4)	9.40<br>(1.88, 16.92)
	**8 months**	101<br>(90, 112)	867<br>(76, 98)	8.08<br>(-0.32, 16.48)	-5.73<br>(-15.03, 3.56)	7.38<br>-1.05774 15.81	-6.35<br>-15.64376 2.95
	**Peak value**	108<br>(97, 120)	99<br>(89, 110)	8.89<br>(2.33, 15.44)	6.01<br>(-1.10, 13.12)	8.39<br>(2.00, 14.78)	5.36<br>(-1.53, 12.25)
	**Peak time-months**	4.57<br>(3.72, 5.43)	3.54<br>(2.57, 4.51)				
**QUADS DOM (kg)**	**Baseline**	18.8<br>(16.7, 20.9)	17.6<br>(15.7, 19.5)				*
	**2 months**	21.6<br>(19.4, 23.8)	20.1<br>(18.1, 22.1)	3.37<br>(1.97, 4.78)	2.44<br>(1.09, 3.78)	3.33<br>(1.91, 4.74)	2.39<br>(1.03, 3.74)
	**8 months**	22.7<br>(20.2, 25.6)	20.6<br>(18.2, 23.0)	4.10<br>(2.48, 5.72)	3.16<br>(1.55, 4.78)	4.06<br>(2.46, 5.65)	3.12<br>(1.52, 4.71)
	**Peak value**	23.5<br>(21.0, 26.0)	21.7<br>(19.3, 24.1)	3.82<br>(2.46, 5.17)	2.83<br>(1.53, 4.13)	3.77<br>(2.43, 5.11)	2.78<br>(1.49, 4.07)
	**Peak time-months**	5.65<br>(4.39,6.91)	5.27<br>(4.19, 6.36)				
**SGRQ-I (Total score)**	**Baseline**	43.2<br>(35.4, 51.1)	36.6<br>(28.5, 44.7)				*
	**2 months**	48.8<br>(41.9, 55.7)	43.0<br>(35.8, 50.2)	-6.57<br>(-11.81, -1.33)	-3.75<br>(-8.92, 1.42)	-6.39<br>(-11.62, -1.17)	-3.54<br>(-8.71, 1.63)
	**8 months**	40.3<br>(31.8, 48.8)	49.2<br>(41.3, 57.1)	-2.57<br>(-8.06, 2.92)	2.15<br>(-3.39, 7.68)	-2.40<br>(-7.89, 3.09)	2.37<br>(-3.18, 7.93)
	**Min value**	33.9<br>(25.1, 42.6)	41.1<br>(33.1, 49.1)	-4.99<br>(-9.62, -0.36)	-1.84<br>(-6.51, 2.83)	-4.82<br>(-9.44, -0.19)	-1.63<br>(-6.31, 3.05)
	**Min time-months**	4.37	3.94				

For peer review

For peer review only - http://bmjopen.bmj.com/site/about/guidelines.xhtml

		(3.68, 5.07)	(3.24, 4.65)				

The quadratic term was significant for all secondary outcomes (p=0.0014 for SNIP, p= 0.004 for QUADS-DOM and p= 0.0014 for SGRQ-I), Figure 3.

SNIP values peaked earlier than 6MWD values in both groups by approximately 2 weeks (Figure 3). QUADS-DOM values were maintained above baseline levels in both groups (Figure 2) and showed a similar time course of change.

There was no evidence for a difference between treatment groups for the SGRQ-I values. The time to reaching the greatest improvement in SGRQ-I score was shorter in the HIIT group, but the changes in the HIIT group were of smaller magnitude at all time-points (Supplementary Tables 1 and 2).

Effects of HIIT on different aetiology ILD

Our models included 3-way interactions between time, intervention (HIIT or MICT) and clinical group, to predict changes from baseline in the combined fibrosis (IPF and CHP group) and CTD-ILD groups. The patterns of change are consistent with the primary analysis – i.e. that whilst the HIIT group peaked faster than the MICT group, the level of improvement was less (Supplementary Tables 1 and 2 and Supplementary Figures 1-4).

Discussion

To our knowledge, this is the first study to investigate the feasibility and potential efficacy of HIIT in the context of a circuit-based clinical PR programme for ILD and compare it with MICT. It demonstrated the feasibility of HIIT, in PR for ILD patients and provided preliminary data on the exercise mode more likely to

For peer review only - http://bmjopen.bmj.com/site/about/guidelines.xhtml

maintain benefits 6 months after programme completion. In addition, we identified the time of peak benefit and time course of subsequent deterioration.

HIIT proved to be feasible and well tolerated but required closer supervision and careful instructions for patients to continue this mode of training than MICT. The attrition rate was higher in the HIIT group compared to the MICT group and more contact was needed in the 6-month period to ensure the HIIT mode of training met the protocol requirements.

The preliminary efficacy of HIIT over MICT was tested using random effects models. Their aim was to understand the average variation of the four key outcomes through time across groups to answer five main questions: a) How do these measurements change over time?; b) Does the benefit differ between groups?; c) How sustainable is the benefit? d) Does the attrition rate differ between groups? e) What is the influence of age? The study was primarily designed to compare the two treatment groups, but an exploratory analysis was performed to look for differences between clinical groups, although this resulted in small numbers in the subgroups.

Contrary to our initial hypothesis, our data showed no evidence of greater benefit with HIIT compared to that achieved with MICT in exercise capacity, respiratory and peripheral muscle function or health status in the medium-term. The study was designed as a pilot to test the potential for a trial of HIIT in a larger, definitive study, but we are forced to conclude that a study of the same design may not be worthwhile.

At 2 months, both groups improved > 34 meters in the 6MWD, which is the reported minimal clinical important difference (MCID) in IPF patients [25, 28, 29], adding to existing evidence on the benefits of PR in ILD [8, 25-26, 30, 32-33, 43]. However, although the 6MWD improved earlier in the HIIT group (peaking at 4 months instead of 5 months) differences from baseline were consistently lower and not as well maintained at 8 months compared with the MICT group. A similar pattern emerged when the effect of age was added to the model. Age is an important factor in indices associated with mortality risk in ILD [3, 4] and our results showed lower 6MWD with greater age. Despite this age effect, PR had a positive effect in all age groups

For peer review only - http://bmjopen.bmj.com/site/about/guidelines.xhtml

regardless of baseline levels, contrary to previous studies reporting greater improvement in patients with lower baseline 6MWD [8, 29, 33, 34]. However, HIIT did not offer additional or preferential benefits to any specific age group and tended to decline below baseline levels at 8 months in the older group. In contrast, the benefit seen in the older MICT group was maintained above baseline levels.

In addition to functional exercise capacity, patients improved quadriceps muscle strength post-PR but similarly, there was no difference between treatment groups. Age-adjusted results for quadriceps force were less variable than for 6MWD and benefits were well maintained above baseline in both groups. The modelling suggested that quadriceps force peaked a little later than other outcomes at 5.5 months in the MICT group and at 5 months at the HIIT group. Our results support the inclusion of strength training in ILD-PR programmes and suggest that it may explain why some studies showed greater improvement and maintenance in functional capacity [26] while others that focused on endurance training did not [8]. The time course of peak improvement and subsequent decline in quadriceps strength did not follow the pattern seen with 6MWD, in either group. Quadriceps strength has been shown to deteriorate in line with ageing and severity in other chronic respiratory disorders [35] and has been identified as an important predictor of mortality in COPD [36]. Skeletal muscle atrophy has also been identified in advanced ILD [37]. Our ILD group, which included people in advanced stage (as defined by the ILD-GAP score > 5, Table 1) showed improvement in mean quadriceps force post-PR in both groups and across all age groups. We found no evidence that older individuals benefited more from PR.

Respiratory muscle strength was preserved at baseline (mean SNIP, MIP and MEP > 70 cmH_2O in both groups) as previous described [38, 39] and improved post-PR in both groups even though there was no inclusion of specific inspiratory muscle training in our programme. In addition, the SNIP followed a similar pattern over time to 6MWD and peaked approximately two weeks earlier than the 6MWD in both the HIIT and MICT group. Although there was no additional benefit with HIIT, our results suggest that SNIP may be more useful in monitoring changes following PR than quadriceps force in ILD patients. In support of this

For peer review only - http://bmjopen.bmj.com/site/about/guidelines.xhtml

conclusion, Mendoza and colleagues [38] showed that inspiratory muscle strength correlated better with 6MWD than quadriceps twitch force in patients with fibrotic idiopathic interstitial pneumonia. Although our ILD group was more mixed and included CTD-ILD patients, our results confirm that respiratory muscle strength appears preserved in ILD and that inspiratory muscle strength correlates well with the 6MWD. Our study is the first to show that inspiratory muscle strength changes in tandem with the 6MWD following PR, regardless of the chosen mode of training.

Health status, as defined by the SGRQ-I and HAD scale improved post-PR and was maintained at 8 months, but again, there was no evidence of superiority of HIIT over the MICT group (Table 1- online supplement).

Effects of HIIT on different aetiology ILD

Random effect models were also created based on ILD type (clinical subgroups). We combined the IPF and CHP subgroups into the combined 'Fibrosis' clinical group. This was due to smaller numbers of IPF participants at the end of the study which did not allow for independent evaluation of this subgroup.

The effects of HIIT on ILD of different aetiology were consistent with our primary analysis, i.e. that HIIT peaked faster and the level of improvement was less. This result seemed to be largely attributable to the fibrosis group, which peaked fastest but showed the lowest improvement from baseline compared to the CTD-ILD group. Similar findings were observed for the other outcomes, again showing lack of preferential benefit from HIIT in any subgroup. Whilst the CTD-ILD subgroup improved in most variables, the HIIT mode was not superior to MICT.

Limitations

The study has limitations. First, despite stratification during the randomisation process, the DLCO was lower in the HIIT group than the MICT group (Table 1). Although DLCO did not affect PR benefits in a previous study [40], it may reflect a greater degree of vascular comorbidity in the HIIT group. Future definitive studies will need to control this factor via randomisation processes such as minimisation.

For peer review only - http://bmjopen.bmj.com/site/about/guidelines.xhtml

We did not use supplementary oxygen during PR for participants other than for those already on long term oxygen therapy (LTOT). Although promising benefits have been reported with acute oxygen supplementation during exercise in IPF patients [41], there is still insufficient evidence about medium to long-term benefits in ILD patients [27] For our study, we felt it was important to train participants to recognise the time it took before desaturation occurred, so that they were able to continue that pattern of training and monitor themselves when unsupervised in community. Participants already on LTOT continued to exercise with oxygen. However, none of the three participants on LTOT finished PR, regardless of group allocation, supporting previous reports that PR may not be appropriate for them [42]. Future studies need to explore different models of training for these cases, such as home-based programmes. Finally, the number of IPF participants who remained in the study was small and did not allow us to investigate the effect of HIIT over time in this particular group.

Implications for future definitive studies

This pilot, enabled us to explore the effect of mode of training on an ILD-PR programme of 8-weeks duration followed by community-based training for 6 months. Our programme duration reflects current UK-based clinical programmes and showed improvements that were maintained at 8 months and were similar to a recent study of a much longer PR programme duration [26]. Although we conclude that there is no evidence to support the use of HIIT in a future definitive study of similar design to this pilot, our results suggest that HIIT may have a role when quicker improvements are required, such as in pre-operative rehabilitation for ILD and that HIIT is feasible to be investigated further in a larger, definitive study of different design. Our results also provide valuable information on the time point where 6MWD and other variables start to deteriorate post-PR and therefore, an estimate of time when additional PR support may be needed, thus optimising PR provision for ILD patients.

Conclusions

For peer review only - http://bmjopen.bmj.com/site/about/guidelines.xhtml

HIIT is feasible and improves exercise capacity but this exercise modality showed no evidence of superiority over MICT in a study design that reflected clinical ILD-PR programmes. Indeed, our findings suggest that it may be less effective and may require closer monitoring to avoid exercise-induced desaturation so, until further evidence becomes available, MICT appears to be more appropriate in the general clinical setting.

Author Contributions: Concept and Design of Study: DN, PJ, FC, RA, IC-S; Acquisition of Data: DN, CL, IMcN ; Analysis of Data: DN, IC-S; Drafting of Manuscript: DN, IC-S, PJ ; Revision of manuscript critically for important intellectual content: All authors; Approval of final manuscript: All authors

Competing interests: In the last 36 months:

DN, IMN and CYL declare no financial interests.

ICS- has had grants from NIHR, Astra-Zeneca and BHF and is a statistical expert for the Health Research Authority

FC- has received speaker fees and financial sponsorship for conducting meetings by Boehringer-Ingelheim and Roche

RA- has received lecture fees from Pfizer and Novartis

PJ- owns GlaxoSmithKline shares

Ethics statement: Ethical approval was secured through the South East Coast-Surrey NRES committee (REC reference: 14/LO/0149) on the 14th February 2014.

Funding statement: The study was funded by the National Institute for Health Research (NIHR) under its Research for Patient Benefit (RfPB) Programme (Grant Reference Number PB-PG-1112-29067). The views expressed are those of the authors and not necessarily those of the NHS, the NIHR or the Department of Health. The primary trial sponsor was St George's Hospital NHS Foundation Trust (Cranmer Terrace, London, SW17 ORE).

Data sharing statement: Data are available upon reasonable request. All relevant data are presented in this paper; and more information can be provided upon reasonable request from the corresponding author

References

For peer review only

1. Travis WD, Costabel U, Hansell DM, et al. ATS/ERS Committee on Idiopathic Interstitial Pneumonias. An official American Thoracic Society/European Respiratory Society statement: Update of the international multidisciplinary classification of the idiopathic interstitial pneumonias. Am J Respir Crit Care Med 2013 Sep 15; 188 (6): 733-48.
2. Wells AU and Hirani N. BTS Interstitial lung disease guideline: the British Thoracic Society in collaboration with the Thoracic Society of Australia and New Zealand and the Irish Thoracic Society. Thorax 2008; 63 (Suppl V): v1- v58.
3. Ley B, Ryerson CJ, Vittinghoff E, et al. A multidimensional index and staging system for idiopathic pulmonary fibrosis. Ann Intern Med. 2012; 156 (10): 684-91.
4. Ryerson CJ, Vittinghoff E, Ley B, et al. Predicting survival across chronic Interstitial Lung Disease- The ILD-GAP model. Chest 2014; 145 (4): 723-28.
5. Dowman L, Hill CJ, Holland AE. Pulmonary rehabilitation for interstitial lung disease. Cochrane Database Syst Rev. 2014 Jan 1;10.
6. Spruit M. A., Singh S., Garvey C, et al. An Official American Thoracic Society/European Respiratory Society statement: Key concepts and advances in Pulmonary Rehabilitation. Am J Resp Crit Care Med 2013; Vol 188 (no 8): e13-e64.
7. Holland AE, Hill CJ, Conron M, *et al*. Short term improvement in exercise capacity and symptoms following exercise training in interstitial lung disease. Thorax 2008; 63: 549-554.
8. Dowman L. M, McDonald C.F., Hill C.J, *et al.* The evidence of benefits of exercise training in interstitial lung disease: a randomised controlled trial. Thorax 2017; 0: 1-10.
9. Helgerud J, Høydal K, Wang E, Karlsen T, Berg P, Bjerkaas M, et al. Aerobic high-intensity intervals improve VO_2max more than moderate training. Med Sci Sports Exerc 2007; 39 (4):665–71.
10. Levinger et al. What Doesn't Kill You Makes You Fitter: A Systematic Review of High-Intensity Interval Exercise for Patients with Cardiovascular and Metabolic Diseases. *Clinical Medicine Insights: Cardiology* 2015:9 53–63.
11. Arena R, Myers J, Forman DE, Lavie CJ, Guazzi M. Should high-intensity-aerobic interval training become the clinical standard in heart failure? *Heart Fail Rev*. 2013; 18 (1): 95–105.
12. Shiraev T and Barclav G. Evidence based exercise- Clinical benefits of high intensity interval training. Aust Fam Physician 2012; 41 (12): 960-962.
13. Wisloff U, Stovlen A, Loennechen JP, et al. Superior cardiovascular effect of aerobic interval training versus moderate continuous training in heart failure patients. Circulation 2007; 115: 3086-3094.
14. Vogiatzis I, Gerasimos Terzis, Serafeim Nanas, *et al*. Skeletal muscle adaptations to interval training in patients with advanced COPD. Chest 2005; 128: 3838-3845.
15. Vogiatzis I, Nanas S, Kastanakis E, et al. Dynamic hyperinflation and tolerance to interval exercise in patients with advanced COPD. Eur Respir J 2004; 24: 385–390.
16. Kortianou EA, Nasis IG, Spetsioti ST, et al. Effectiveness of interval exercise training in patients with COPD. Cardiopulm Phys Ther J 2010; 21: 12–19.
17. Beauchamp M. A, Nonoyama M., Goldstein R. S., et al. Interval versus continuous training in individuals with chronic obstructive pulmonary disease- a systematic review. Thorax 2010; 65: 157-164.
18. Gloeckl R, Halle M, Kenn K. Interval versus continuous training in lung transplant candidates: a randomized trial. J Heart Lung Transplant 2012; 31: 934–941.

For peer review only - http://bmjopen.bmj.com/site/about/guidelines.xhtml

19. Nikoletou D, Lech C. Y., MacNaughton I.S, Chis-Ster I., Chua F., Aul R., Jones P.. High intensity interval versus moderate intensity continuous training in pulmonary rehabilitation for patients with interstitial lung disease. - American Thoracic Society Conference- B109. HIGHLIGHTS AND ADVANCES IN PULMONARY REHABILITATION. May 2016, A4520-A4520 San Francisco, USA.
20. Borg G: Perceived exertion as an indicator of somatic stress. Scand J Rehabil Med. 1970, 2: 92-98.
21. Borg GA: Psychophysical bases of perceived exertion. Med Sci Sports Exerc. 1982, 14: 377-381
22. ATS statement: guidelines for the six-minute walk test. Am J Respir Crit Care Med 2002; 166: 111–17.
23. Yorke J, Jones P, Swigris J J. Development and validity testing of an IPF-specific version of the St George's Respiratory questionnaire. Thorax 2010; 65: 921-926.
24. Zigmond AS and Snaith RP: The Hospital Anxiety And Depression Scale Acta Psychiatr Scand 1983, 67:361-70
25. Holland AE, Hill CJ, Connon M, *et al*. Short term improvement in exercise capacity and symptoms following exercise training in interstitial lung disease. Thorax 2008; 63: 549-554.
26. Perez-Bogerd S., Wuyts W, Barbier V, Demeyer H, Van Muylem A, Janssens W, Troosters T. Short and long-term effects of pulmonary rehabilitation in interstitial lung diseases: a randomised controlled trial. Respiratory Research 2018; 19 (1): 182.
27. Bell EC, Cox NS, Goh N, et al. Oxygen therapy for interstitial lung disease: a systematic review. Eur Respir Rev 2017: 26: 160080 [https://doi.org/10.1183/16000617.0080-2016]
28. Holland AE, Hill CJ, Connon M, *et al*. Small changes in six-minute walk distance are important in diffuse parenchymal lung disease. Respir Med 2009; 103:1430-5.
29. du Bois RM, Weycker D, Albera C, et al. Six-minute walk test in idiopathic pulmonary fibrosis; test validation and minimal clinically important difference. Am J Respir Crit Care Med, 2011; 183: 1231-1237.
30. Tonelli R, Cocconcelli E, Lanini B, Romagnoli I et al. Effectiveness of pulmonary rehabilitation in patients with interstitial lung disease of different etiology; a multicentre prospective study. BMC Pulmonary Medicine 2017; 17: 1-9.
31. Nishiyama O, Kondoh Y, Kimura T, Kato K, Kataoka K, Ogawa T, Watanabe F, Arizono S, Nishimura K, Taniguchi H. Effects of pulmonary rehabilitation in patients with idiopathic pulmonary fibrosis. Respirology. 2008;13:394–9.
32. Vainshelboim B, Oliveira J, Yehoshua L, Weiss I, Fox BD, Fruchter O, Kramer MR. Exercise training-based pulmonary rehabilitation program is clinically beneficial for idiopathic pulmonary fibrosis. Respiration. 2014;88:378–88.
33. Ferreira A, Garvey C, Connors GL, Hilling L, Rigler J, Farrell S, Cayou C, Shariat C, Collard HR. Pulmonary rehabilitation in interstitial lung disease: benefits and predictors of response. Chest. 2009;135:442–7.
34. Ryerson CJ, Cayou C, Topp F, et al. Pulmonary rehabilitation improves long-term outcomes in interstitial lung disease: a prospective cohort study. Respir Med 2014;108:203–10.
35. Seymour JM, Spruit MA, Hopkinson NS, et al. The prevalence of quadriceps weakness in COPD and the relationship with disease severity. Eur Respir J 2010; 36(1): 81–88.
36. Swallow EB, Reyes D, Hopkinson NS, et al. Quadriceps strength predicts mortality in patients with moderate to severe chronic obstructive pulmonary disease. Thorax 2007; 62(2): 115–120.

For peer review only

For peer review only - http://bmjopen.bmj.com/site/about/guidelines.xhtml

37. Mendes P, Wicherson L, Helm D, et al. Skeletal muscle atrophy in advanced interstitial lung disease. Respirology 2015; 20: 953-959.
38. Mendoza L, Gogali A, Shrikrishna D, Cavada G, et al. Quadriceps strength and endurance in fibrotic idiopathic interstitial pneumonia. Respirology 2014; 19: 138-143.
39. de Troyer A, Yernault JC, Inspiratory muscle force in normal subjects and patients with interstitial lung disease. Thorax 1980; 35: 92-100.
40. Tonelli R, Cocconcelli E, Lanini B, et al. Effectiveness of pulmonary rehabilitation in patients with interstitial lung disease of different etiology; a multicentre prospective study. BMC Pulmonary Medicine 2017; 17: 130-139.
41. Dowman LM, McDonald CF, Bozinovski S, Vlahos R, Gillies R, Pouniotis D, Hill CJ, Goh NSL, Holland AE. Greater endurance capacity and improved dyspnoea with acute oxygen supplementation in idiopathic pulmonary fibrosis patients without resting hypoxaemia. Respirology 2017; 22: 957-964.
42. Johnson-Warrington V, Williams J, Bankart J, Steiner M, Morgan M, Singh S. Pulmonary rehabilitation and Interstitial lung disease: Aiding the referral decision. J Cardiopulm Rehab and Prevent 2013; 33 (3): 189-195.
43. Jackson RM, Gomez-Marin OW, Ramos CF, Sol CM, Cohen MI, Gaunaurd IA, Cahalin LP, Cardenas DD. Exercise limitation in IPF patients: a randomized trial of pulmonary rehabilitation. Lung. 2014;192:367–76.

For peer review only

For peer review only - http://bmjopen.bmj.com/site/about/guidelines.xhtml

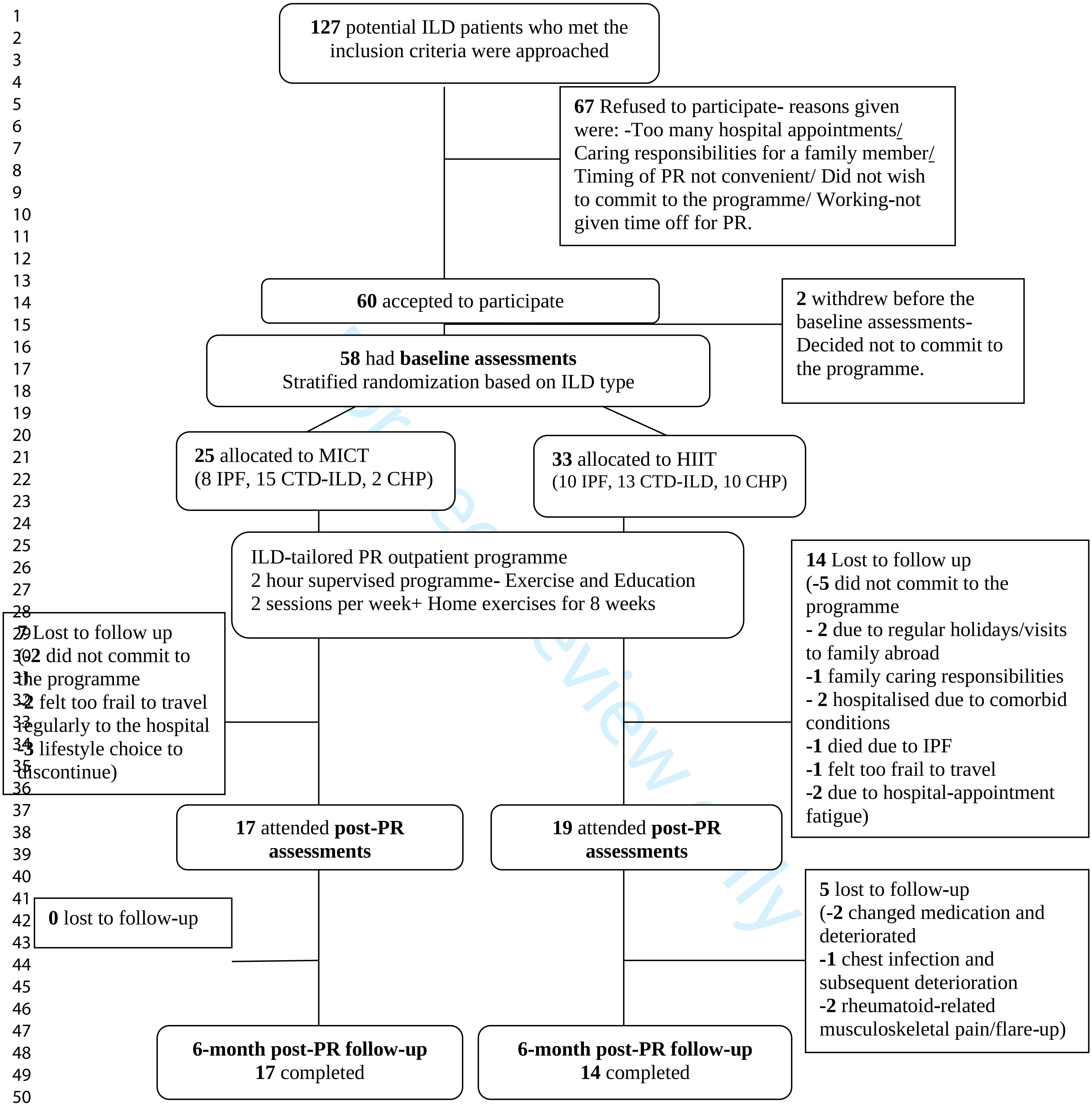

Figure 1: CONSORT diagram of participants' flow in the study. ILD-PR= pulmonary rehabilitation programme tailored for interstitial lung disease patients, MICT= moderate intensity continuous training, HIIT= high intensity interval training, IPF= idiopathic pulmonary fibrosis, CTD-ILD= connective tissue disease related interstitial lung disease, CHP= chronic hypersensitivity pneumonitis.

For peer review only - http://bmjopen.bmj.com/site/about/guidelines.xhtml

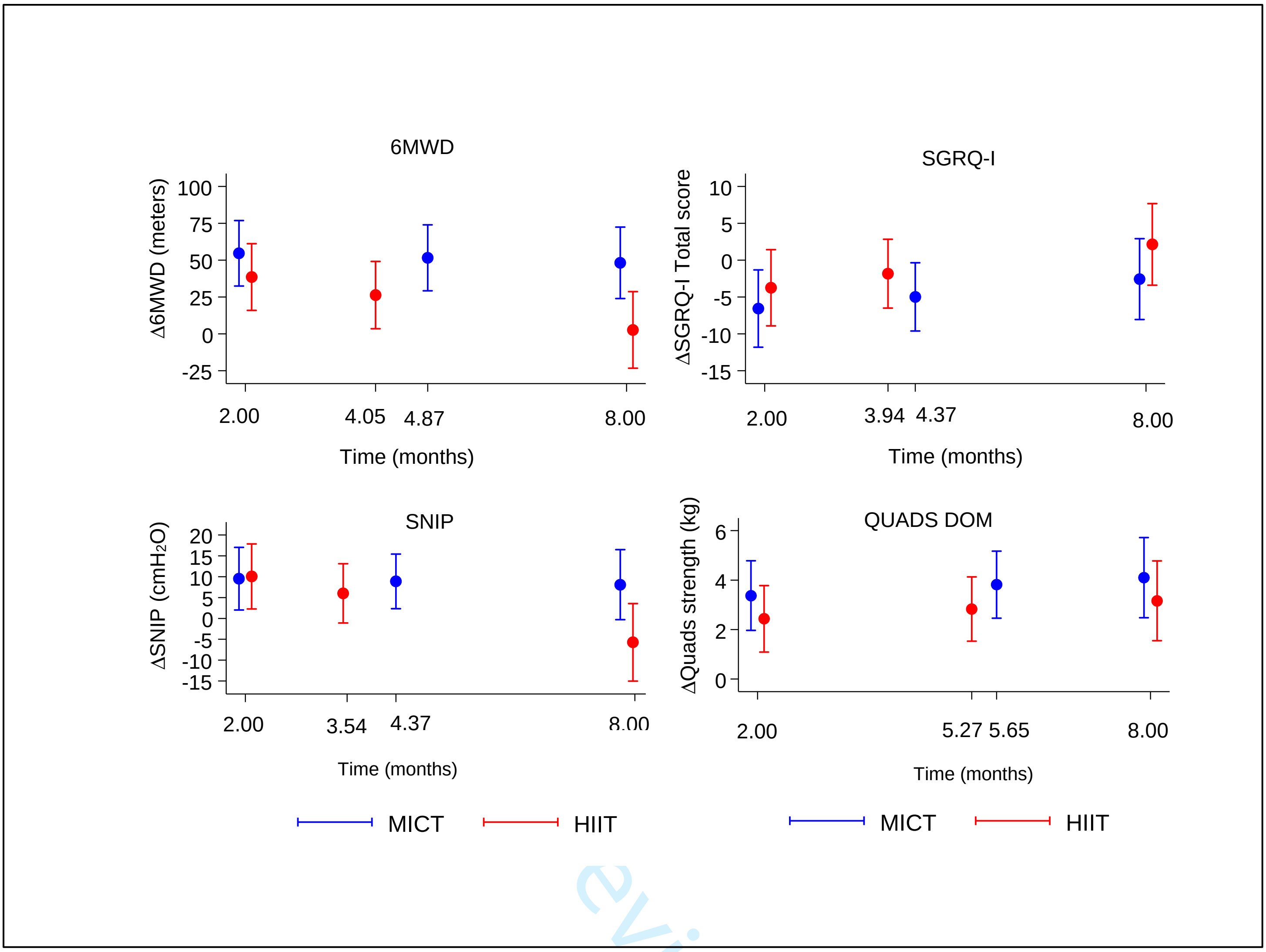

Figure 2: Change from baseline in 4 key outcomes by intervention group over time.

eview only

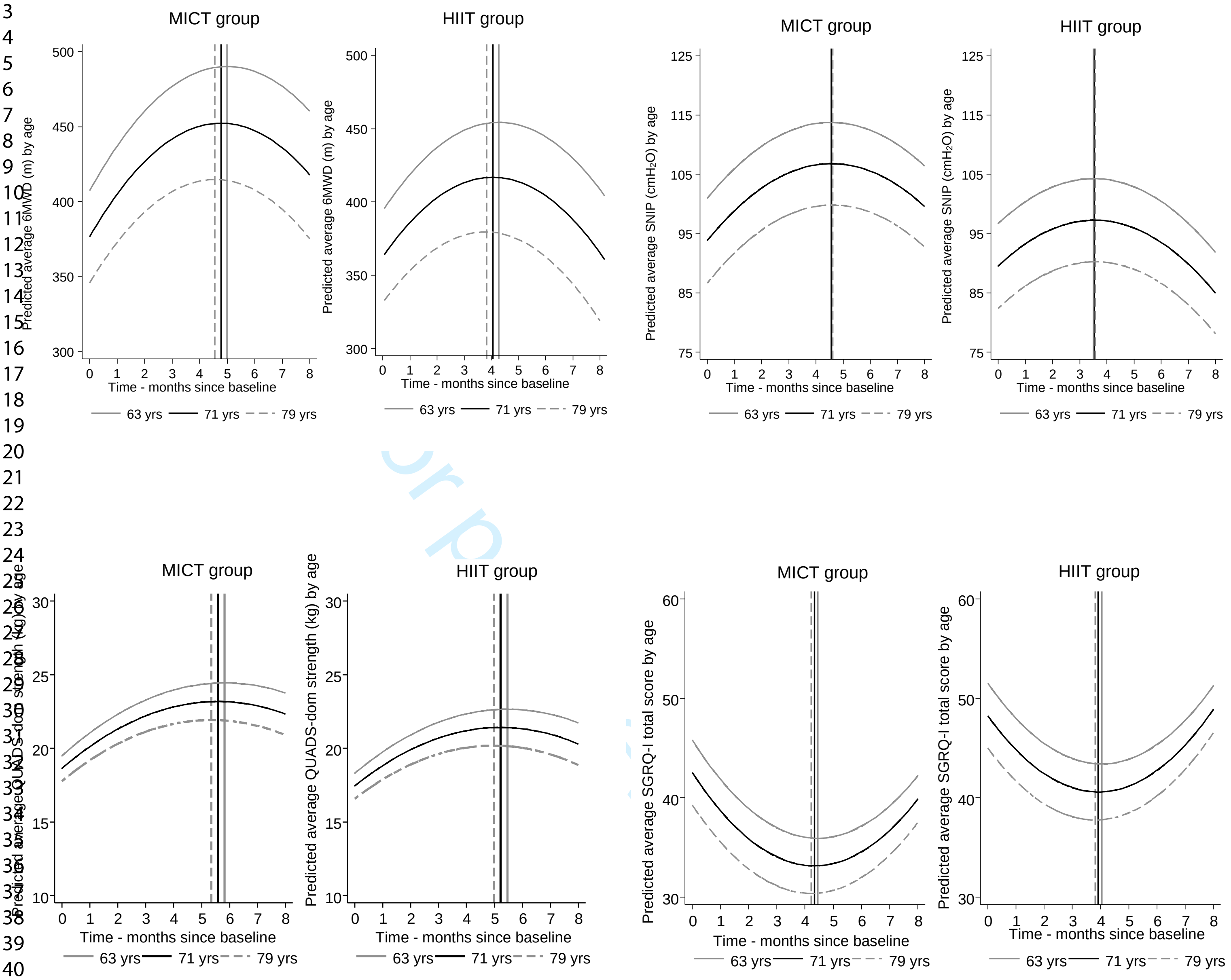

Figure 3: Age-related variations in the 4 key outcomes (6MWD, SNIP, QUADS-DOM (Quadriceps strength) and SGRQ-I total score) per training group using random effects models. The vertical line indicates the inflection point (i.e. highest or lowest values) in each age group. The ages are the mean values in each tertile

For peer review only - http://bmjopen.bmj.com/site/about/guidelines.xhtml

Table 1: Descriptive values at each assessment point

	Control group (MICT) Mean (SD)			Intervention group (HIIT) Mean (SD)		
	**Baseline N= 25**	**2M (Post-PR) N=17**	**8M (6M post-PR) N= 17**	**Baseline N=33**	**2M (Post-PR) N=19**	**8M (6M post-PR) N=14**
**BMI (kg/m²)**	27.5 (3.8)	28.3 (4.1)	27.8 (3.9)	27.2 (5.1)	26.5 (4.9)	26.0 (4.7)
**Waist/Hip ratio**	0.9(0.1)	0.9 (0.1)	0.9 (0.0)	0.9 (0.1)	0.9 (0.1)	0.9 (0.1)
**%fat**	29.9 (9.3)	33.2 (9.8)	33.7 (8.7)	30.1 (8.1)	27.9 (10.7)	28.5 (9.6)
**FVC%,pred.**	85.8 (23.9)	88.0 (17.5)	88.7 (24.4)	73.8 (18.8)	68.4 (22.4)	71.3 (24.1)
**DLCO%, pred.**	53.9 (13.1)	53.6 (13.5)	56.5 (21.1)	41.7 (13.9)	44.9 (20.2)	44.8 (20.3)
**6MWD (m)**	380 (139.8)	453.9 (108.1)	437.4 (107.9)	364 (108.3)	434.3 (102.3)	421.9 (123.0)
**SNIP (cmH_2O)**	96.8 (25.9)	103.0 (21.04)	97.8 (21.4)	93.1 (24.5)	102.2 (34.1)	95.3 (29.5)
**PImax(cmH_2O)**	91.1(34.2)	104.3 (28.5)	104.9 (24.0)	91.8 (27.4)	92.8 (28.6)	95 (31.7)
**PEmax(cmH_2O)**	114.4 (40.3)	111.1 (33.3)	106.8 (34.5)	100.7 (23.5)	105.1 (24.2)	106.9 (24.7)
**Handgrip (kg)**	27.2 (11.2)	28.4 (8.4)	27.4 (9.3)	24.8 (9.4)	26.2 (11.4)	27.4 (11.5)
**Quads extension (dom) (kg)**	18.7 (6.2)	21.8 (5.0)	22.8 (6.1)	18.0 (5.0)	20.0 (5.6)	21.5 (5.4)
**Hip flexion (dom) (kg)**	15.3 (5.1)	17.9 (4.1)	18.5 (5.1)	15.9 (4.3)	16.5 (4.7)	18.1 (6.3)
**HAD-A**	6.2 (4.6)	4.8 (3.3)	5.1 (3.7)	6.4 (3.9)	6.4 (4.6)	7.2 (4.2)
**HAD-D**	5.8 (3.2)	5.0 (3.3)	5.2 (2.3)	5.2 (4.0)	5.1 (3.4)	6.6 (4.2)
**SGRQ-I Total score**	43.3 (20.3)	35.1 (15.3)	41.5 (19.3)	49.3 (21.8)	40.2 (16.7)	48.9 (21.6)

For peer review only

Table 2: Average predictions for main outcome values at the main time points during the trial, i.e. baseline, 2 months, 8 months and *changes from the baseline* at 2 months, 8 months and by the time they reach their max/min values. This set of predictions are made across intervention and ILD clinical groups, adjusting for age (hence predictions are made for patients of average age of 71 years). Estimated peak/saddle times are also displayed.

For peer review only

Outcome	Time	Control (MICT)				Intervention (HIIT)			
		Fibrosis		CTD-ILD		Fibrosis		CTD-ILD	
		Mean (95% CI) predicted value	Change from baseline	Mean (95% CI) predicted value	Change from baseline	Mean (95% CI) predicted value	Change from baseline	Mean (95% CI) predicted value	Change from baseline
6MWD	**Baseline**	415.84 (360.8 to 470.9))		393.89 (337.8 to 450.0)		367.94 (327.4 to 408.5)		358.88 (304.8 to 413.0)	
	**2 months**	464.63 (409.4 to 519.84)	45.5 (17.5 to 73.5)	442.92 (387.3 to 498.6)	55.4 (35.3 to 75.6)	401.22 (360.1 to 442.3)	28.9 (5.7 to 52.0)	410.63 (356.3 to 465.0)	38.3 (14.2 to 63.5)
	**8 months**	459.03 (398.5 to 519.6)	39.2 (11.2 to 67.2)	438.02 (381.6 to 494.5)	49.2 (28.8 to 69.6)	349.07 (304.9 to 393.3)	- 6.84 (-30.9 to 17.3)	413.93 (351.5 to 476.4)	3.1 (-23.5 to 29.7)
	**Peak value**	490.40 (432.7 to 548.1)	42.5 (15.2 to 69.8)	469.02 (412.6 to 525.5)	52.4 (33.1 to 71.8)	409.60 (366.8 to 452.4)	19.2 (-3.3 to 41.7)	440.80 (382.9 to 498.7)	20.4 (-4.2 to 45.1)
	**Peak time-months**	4.85 (4.1 to 5.6)		4.87 (4.4 to 5.4)		3.63 (3.1 to 4.2)		5.09 (4.3 to 5.9)	
SNIP	**Baseline**	99.94 (86.3 to 113.5)		90.69 (77.4 to 104.0)		96.92 (86.5 to 107.3)		77.55 (64.5 to 90.6)	
	**2 months**	106.89 (93.4 to 120.4)	14.3 (4.0 to 24.5)	100.33 (87.2 to 113.4)	10.9 (3.7 to 18.0)	102.38 (91.6 to 113.1)	8.6 (0.2 to 16.9)	85.46 (72.4 to 98.6)	5.2 (-3.9 to 14.2)
	**8 months**	97.52 (79.9 to 115.2)	5.8 (-5.3 to 16.9)	99.04 (84.9 to 113.4)	2.4 (-5.6 to 10.5)	88.54 (75.3 to 101.8)	0.1 (-9.4 to 9.7)	78.98 (62.5 to 95.5)	-3.3 (-13.2 to 6.6)
	**Peak value**	108.84 (94.3 to 123.4)	11.8 (1.8 to 21.8)	105.37 (91.6 to 119.2)	9.2 (2.4 to 16.0)	103.23 (91.7 to 114.8)	4.6 (-3.6 to 12.6)	88.35 (73.9 to 102.7)	2.2 (-6.6 to 10.9)
	**Peak time-months**	3.76 (2.1 to 5.4)		4.83 (3.8 to 5.9)		3.17 (1.9 to 4.5)		4.14 (2.8 to 5.5)	
QUADS DOM	**Baseline**	18.13 (15.4 to 20.9)		19.68 (16.9 to 22.4)		17.66 (15.6 to 19.7)		17.71 (15.1 to 20.3)	
	**2 months**	21.20 (18.4 to 24.0)	4.6 (2.6 to 6.7)	22.10 (19.4 to 24.8)	2.7 (1.2 to 4.2)	20.09 (17.9 to 22.3)	2.8 (1.2 to 4.3)	20.25 (17.6 to 22.9)	2.4 (0.4 to 4.4)
	**8 months**	23.64 (19.7 to 27.6)	5.4 (3.2 to 7.7)	22.59 (19.6 to 25.6)	3.5 (1.8 to 5.1)	20.62 (17.9 to 23.4)	3.5 (1.9 to 5.2)	21.10 (17.5 to 24.7)	3.2 (1.0 to 5.3)
	**Peak value**	23.99 (20.4 to 27.5)	5.2 (3.1 to 7.3)	23.63 (20.7 to 26.5)	3.1 (1.7 to 4.5)	21.64 (19.1 to 24.2)	3.2 (1.8 to 4.6)	21.98 (18.8 to 25.2)	2.9 (0.9 to 4.8)

For peer review only - http://bmjopen.bmj.com/site/about/guidelines.xhtml

	**Peak time-months**	6.44 (4.2 to 8.7)		5.29 (4.0 to 6.6)		5.31 (4.0 to 6.6)		5.50 (3.8 to 7.2)	
**SGRQ-I**	**Baseline**	44.01 (33.2 to 54.9)		38.42 (27.6 to 49.2)		55.00 (47.0 to 63.0)		37.75 (27.6 to 47.9)	
	**2 months**	37.36 (26.4 to 48.3)	- 8.4 (-16.1 to -0.7)	32.11 (21.5 to 42.7)	-6.6 (-12.0 to -1.2)	49.72 (41.5 to 58.0)	-3.5 (-9.6 to 2.5)	31.38 (21.2 to 41.6)	- 6.5 (-13.9 to 0.9)
	**8 months**	40.39 (27.4 to 53.4)	3.1 (- 10.9 to 4.7)	36.18 (25.2 to 47.2)	-1.3 (-6.7 to 4.2)	56.87 (47.6 to 66.1)	1.8 (-4.3 to 7.8)	35.28 (23.2 to 47.4)	-1.2 (-8.7 to 6.35)
	**Min value**	34.43 (22.6 to 46.3)	-6.2 (-13.7 to 1.3)	29.59 (18.6 to 40.6)	- 4.6 (-9.7 to 0.5)	48.25 (39.3 to 57.2)	- 1.9 (-7.8 to 3.8)	28.80 (17.8 to 39.8)	-4.4 (-11.7 to 2.8)
	**Min time-months**	4.47 (3.2 to 5.8)		4.29 (3.5 to 5.1)		3.76 (2.9 to 4.7)		4.32 (3.2 to 5.5)	

Data are mean (95% CI)

For peer review only

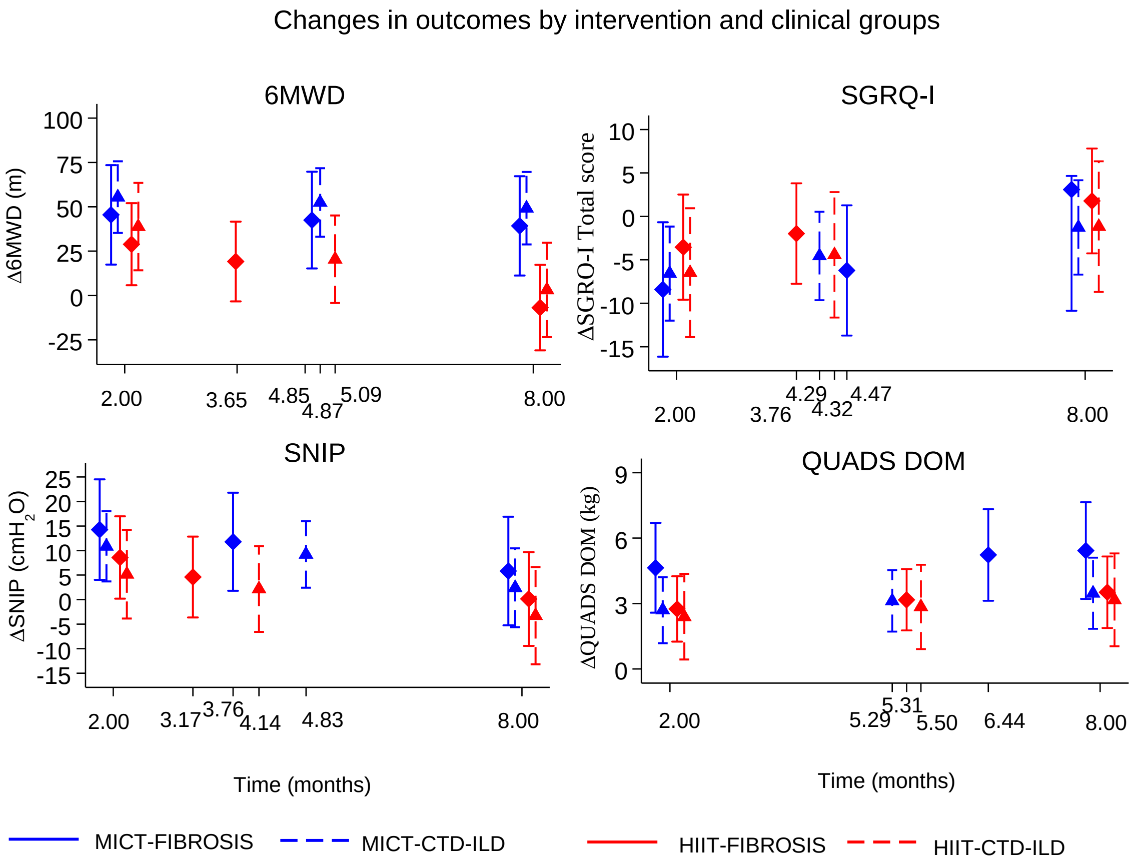

Figure 1: Changes in key outcomes by intervention and ILD clinical groups

For peer review only

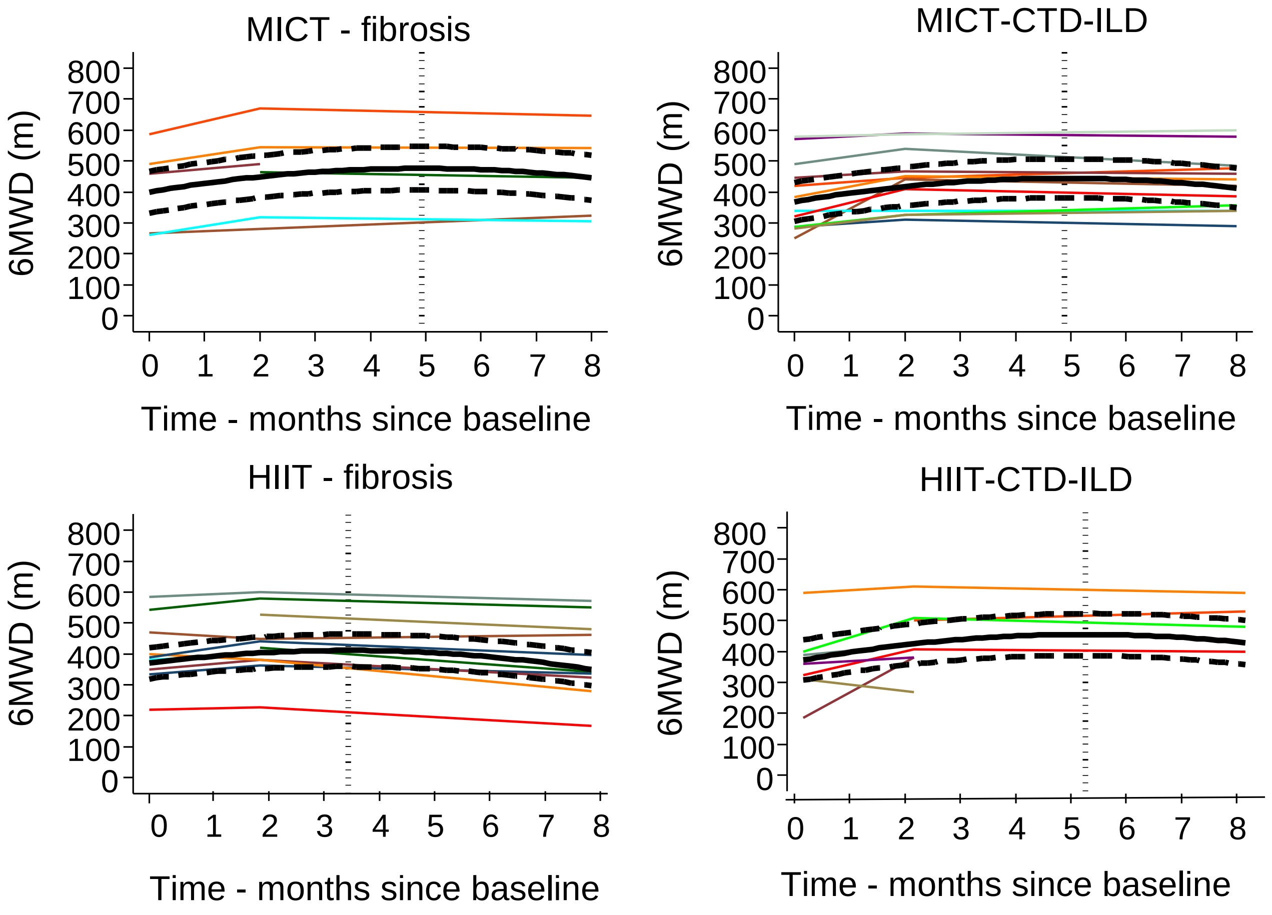

Figure 2: Random effects models by intervention (HIIT vs MICT) and ILD clinical subgroup (Fibrosis vs CTD-ILD)

For peer review only

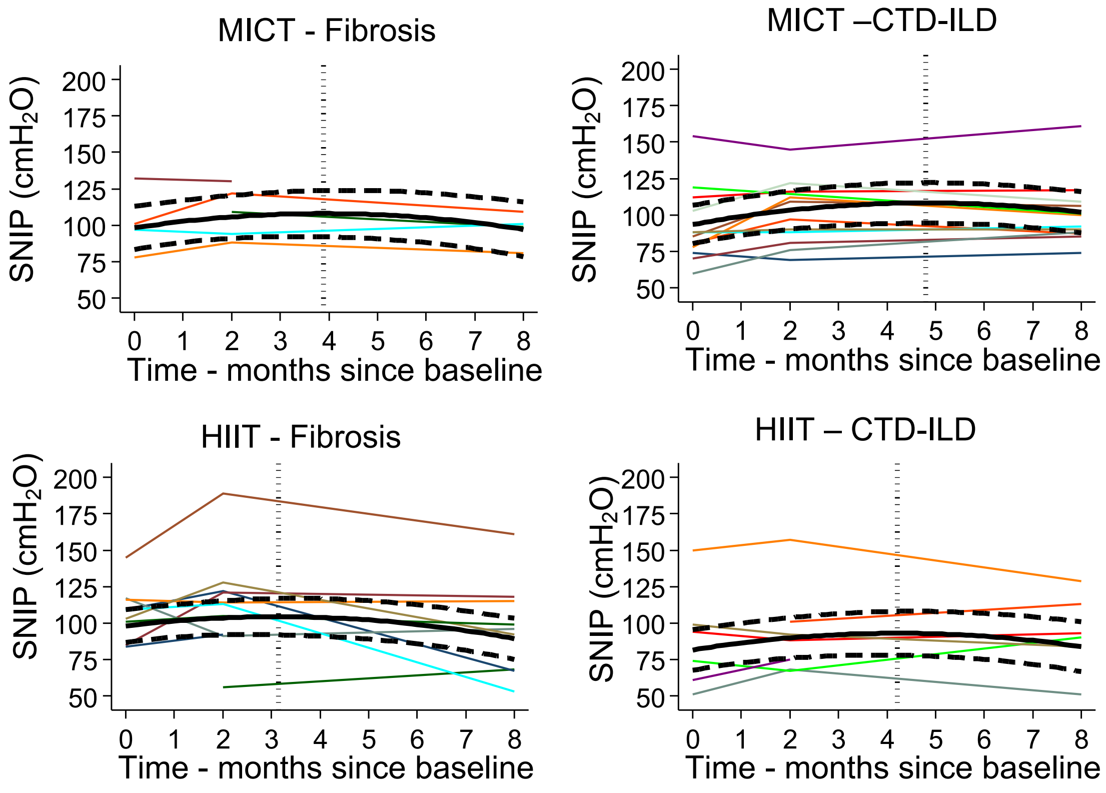

Figure 3: Random effects models by intervention (HIIT vs MICT) and ILD clinical subgroup (Fibrosis vs CTD-ILD)

For peer review only - http://bmjopen.bmj.com/site/about/guidelines.xhtml

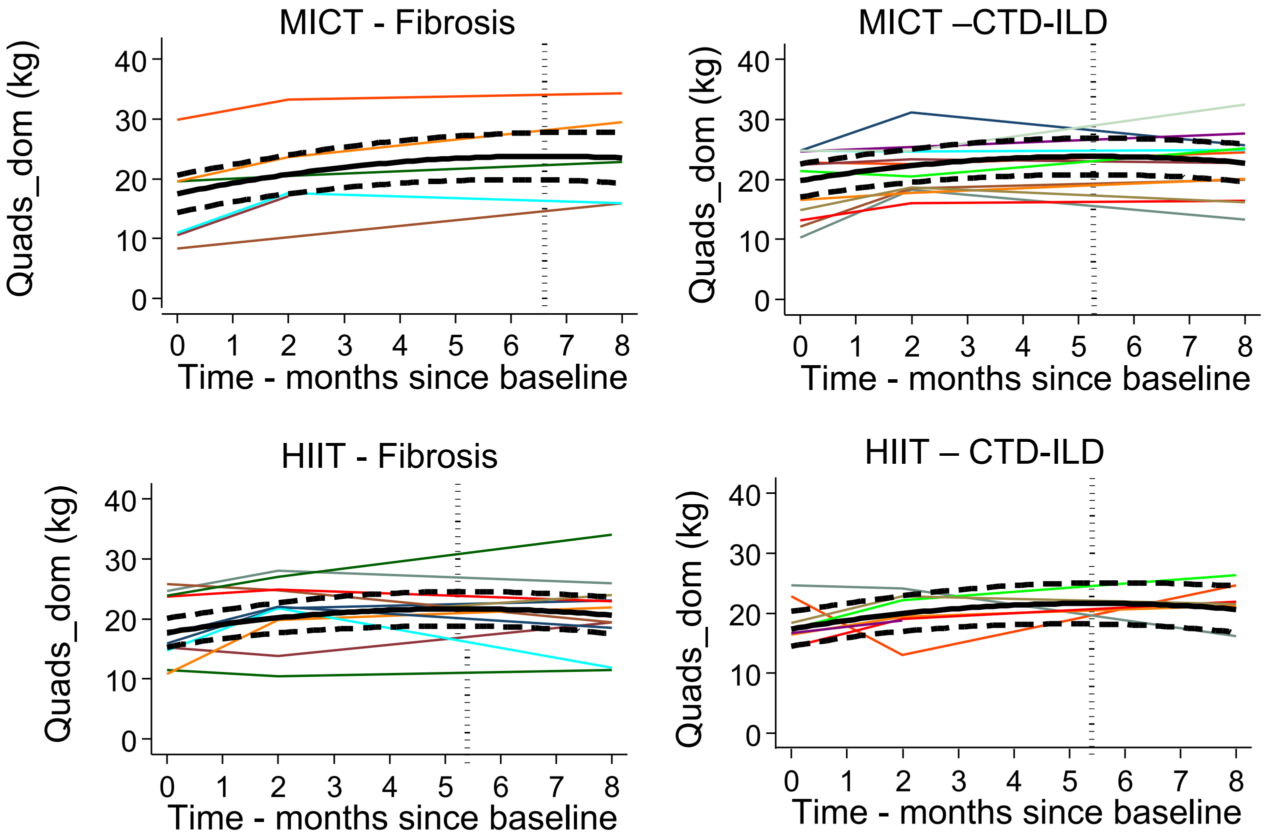

Figure 4: Random effects models of quadriceps strength (dominant side) by MICT vs HIIT and ILD clinical subgroups.

Reporting checklist for randomised trial.

Based on the CONSORT guidelines.

Instructions to authors

Complete this checklist by entering the page numbers from your manuscript where readers will find each of the items listed below.

Your article may not currently address all the items on the checklist. Please modify your text to include the missing information. If you are certain that an item does not apply, please write "n/a" and provide a short explanation.

Upload your completed checklist as an extra file when you submit to a journal.

In your methods section, say that you used the CONSORTreporting guidelines, and cite them as:

Schulz KF, Altman DG, Moher D, for the CONSORT Group. CONSORT 2010 Statement: updated guidelines for reporting parallel group randomised trials

For peer review only

		Reporting Item	Page Number
**Title and Abstract**			
Title	#1a	Identification as a randomized trial in the title.	1
Abstract	#1b	Structured summary of trial design, methods, results, and conclusions	2
**Introduction**			
Background and objectives	#2a	Scientific background and explanation of rationale	4

Background and objectives	#2b	Specific objectives or hypothesis	5
**Methods**			
Trial design	#3a	Description of trial design (such as parallel, factorial) including allocation ratio.	5 and Figure 1
Trial design	#3b	Important changes to methods after trial commencement (such as eligibility criteria), with reasons	n/a
Participants	#4a	Eligibility criteria for participants	5
Participants	#4b	Settings and locations where the data were collected	5
Interventions	#5	The experimental and control interventions for each group with sufficient details to allow replication, including how and when they were actually administered	6-7
Outcomes	#6a	Completely defined prespecified primary and secondary outcome measures, including how and when they were assessed	8-9
Sample size	#7a	How sample size was determined.	9
Sample size	#7b	When applicable, explanation of any interim analyses and stopping guidelines	n/a

For peer review only

Randomization - Sequence generation	#8a	Method used to generate the random allocation sequence.	6
Randomization - Sequence generation	#8b	Type of randomization; details of any restriction (such as blocking and block size)	6
Randomization - Allocation concealment mechanism	#9	Mechanism used to implement the random allocation sequence (such as sequentially numbered containers), describing any steps taken to conceal the sequence until interventions were assigned	6
Randomization - Implementation	#10	Who generated the allocation sequence, who enrolled participants, and who assigned participants to interventions	6
Blinding	#11a	If done, who was blinded after assignment to interventions (for example, participants, care providers, those assessing outcomes) and how.	6
Blinding	#11b	If relevant, description of the similarity of interventions	6 and table 1
Statistical methods	#12a	Statistical methods used to compare groups for primary and secondary outcomes	9
Statistical methods	#12b	Methods for additional analyses, such as subgroup analyses and adjusted analyses	9

For peer review only

Outcomes	#6b	Any changes to trial outcomes after the trial commenced, with reasons	n/a
**Results**			
Participant flow diagram (strongly recommended)	#13a	For each group, the numbers of participants who were randomly assigned, received intended treatment, and were analysed for the primary outcome	10-Figure 1
Participant flow	#13b	For each group, losses and exclusions after randomization, together with reason	Fig 1
Recruitment	#14a	Dates defining the periods of recruitment and follow-up	Fig 1
Recruitment	#14b	Why the trial ended or was stopped	n/a
Baseline data	#15	A table showing baseline demographic and clinical characteristics for each group	Table 1
Numbers analysed	#16	r	10 -Fig 1 and Table 1
Outcomes and estimation	#17a	For each primary and secondary outcome, results for each group, and the estimated effect size and its precision (such as 95% confidence interval)	10-11

For peer review only

Outcomes and estimation	#17b	For binary outcomes, presentation of both absolute and relative effect sizes is recommended	10-11
Ancillary analyses	#18	Results of any other analyses performed, including subgroup analyses and adjusted analyses, distinguishing pre-specified from exploratory	Supplementary files Tables 3-4 and Figures 4-5-6
Harms	#19	All important harms or unintended effects in each group (For specific guidance see CONSORT for harms)	Fig.1
**Discussion**			
Limitations	#20	Trial limitations, addressing sources of potential bias, imprecision, and, if relevant, multiplicity of analyses	15
Interpretation	#22	Interpretation consistent with results, balancing benefits and harms, and considering other relevant evidence	12-16
Registration	#23	Registration number and name of trial registry	1
Generalisability	#21	Generalisability (external validity, applicability) of the trial findings	2, 15-16
**Other information**			

For peer review only

Interpretation	#22	Interpretation consistent with results, balancing benefits and harms, and considering other relevant evidence	12-16
Registration	#23	Registration number and name of trial registry	1
Protocol	#24	Where the full trial protocol can be accessed, if available	5 and Protocol file
Funding	#25	Sources of funding and other support (such as supply of drugs), role of funders	21

Notes:

- 13a: 10-Figure 1
- 16: 10 -Fig 1 and Table 1
- 18: Supplement Tables 3-4, Fig 4-5-6 The CONSORT checklist is distributed under the terms of the Creative Commons Attribution License CC-BY. This checklist was completed on 29. March 2022 using https://www.goodreports.org/, a tool made by the EQUATOR Network in collaboration with Penelope.ai

For peer review only

19/11/13- Version 1.0

Protocol- HIIT in PR for ILD

Abstract

Interstitial lung disease (ILD) is a term used for a diverse group of pulmonary conditions that affect the lung tissue. ILD is classified in different types, including interstitial pulmonary fibrosis (IPF) that has the worse prognosis from all other types [1]. Patients with ILD experience progressive breathlessness, fatigue and poor exercise capacity and enter a vicious cycle of inactivity and physical deconditioning by avoiding activities that increase their symptoms. One of the most effective ways to break this cycle is pulmonary rehabilitation (PR) [2]. PR programmes are multidisciplinary programmes of exercise and education that improve muscle function, breathlessness and self-management in ILD. However, the optimal exercise prescription for long term benefits is currently unknown for this patient group [3]. In addition, recent data emerged that showed high intensity training interval training (HIIT) to be feasible and effective in severe ILD patients prior to lung transplantation [4]. HIIT involves repeatedly exercising at a high intensity for 30 seconds to several minutes, followed by 1-5 minutes of recovery (low intensity exercise or no exercise). It has been shown to increase patient compliance and to improve cardiovascular and metabolic outcomes in healthy people and patient groups [5] such as heart failure patients [6], and cancer survivors [7]. HIIT has yet to be studied in pulmonary rehabilitation programmes for ILD.

This study aims to investigate the feasibility of HIIT in a pulmonary rehabilitation programme and its long term efficacy in comparison to a moderate intensity continuous training programme (MICT). More specifically, we plan to study the effects of each programme on exercise capacity, breathlessness and health status (health-related quality of life). 60 patients with various types of ILD will be randomised into 2 groups: Group A: PR using MI-CT and Group B: PR using a HIIT programme. Patients will train twice weekly for 8 weeks. The HIIT will be prescribed at >80% VO2max, with the duration of each interval based on each patient's capacity to keep saturation level (SaO2) above 89%. Assessment of outcomes will take place at the beginning, immediately post-PR and 6 months later.

The key questions to be addressed are as follows:

1. What is the relative recruitment of different types of ILD to each programme?
2. What is the retention and adherence of different types of ILD to each programme?
3. Is HIIT equally well tolerated in the various types?
4. What is the efficacy of each programme on exercise capacity in the various types of ILD immediately post-PR programme and 6 months later?
5. Does breathlessness and quality of life improve and are maintained more with an HIIT versus a MICT programme?

The results of our study will inform the sample size and other practical issues before designing a definitive trial and will provide evidence about the long term efficacy of HIIT. The results on the efficacy of HIIT, whether positive or negative will allow us to design more focused pulmonary rehabilitation programmes for patients with various types of ILD. We will monitor closely the response of patients with interstitial pulmonary fibrosis (IPF) to our programme, as this type has the worst prognosis amongst all other types of ILD and so far, no other rehabilitation intervention has shown significant changes in physical function or mortality. We will publish the results of this feasibility study and will engage other interested research groups as early as possible. We expect that the definitive study will lead to important changes in the clinical management of ILD patients across the UK and internationally.

Background and rationale

Describe the background to the research, describing the limitations identified in the evidence base that the research is trying to address Interstitial lung disease (ILD), also known as diffuse parenchymal lung disease, is a group of heterogeneous lung disorders that affect the lung interstitium and lead to fibrosis and a restrictive pattern of breathing. ILD can be classified broadly into four categories: a) idiopathic interstitial pneumonias such as interstitial pulmonary fibrosis, b)

For peer review only - http://bmjopen.bmj.com/site/about/guidelines.xhtml

19/11/13- Version 1.0

interstitial lung disease of known cause such as drugs or occupational dust exposures, c) granulomatous
disorders and d) other forms of ILD [8]. Prognosis varies depending on the type of ILD, with interstitial pulmonary fibrosis (IPF) having the worse prognosis compared to the other types and a mean survival 2-4 years [9]. The disease is estimated to affect up to 9% of people in humid areas of the UK and deaths from IPF in particular are estimated to be between 61.9 per 1 million men and 55.1 per 1 million women in the UK [8]. The clinical symptoms in patients with all types of ILD are breathlessness during functional activities, reduced exercise capacity, fatigue and poor quality of life. Peripheral muscle dysfunction plays a significant role in limiting exercise capacity and contributes to physical deconditioning [10]. Treatment for ILD involves the use of oral corticosteroids that are known to lead to drug-induced myopathy, affecting both the respiratory and peripheral muscles and reducing exercise tolerance even further. Although rehabilitation interventions that improve skeletal muscle strength and endurance are beneficial for ILD patients, there is currently lack of knowledge on what is the optimal exercise regime for long-term benefits [3]. Recent evidence has shown that high intensity interval training (HIIT) is feasible and effective in severe ILD patients prior to lung transplantation [4]. However, this type of training has yet to be used in pulmonary rehabilitation programmes for ILD patients [11]. High-intensity interval training (HIIT) is a type of exercise prescription that induces significant muscle fiber adaptations [5, 6, 7, 12]. It consists of brief bursts of high intensity exercise, usually at > 80% of a patient's VO2max, followed by periods of rest. The duration of each interval varies per patient depending on level of breathlessness or exercise desaturation but usually lasts between 30 seconds to 5 minutes in severe patients.

These short intervals of intense exercise are repeated throughout the hour-long session of the exercise programme and the cumulative effect on muscle function is significant for patients who would otherwise be limited by desaturation or breathlessness. The effects of interval training need to be explored further in patients with ILD in the context of PR programmes. Since pulmonary rehabilitation is intended for all types of ILD, our study will investigate the feasibility of HIIT in patients with a variety of ILD types and severity and will comparethe long term efficacy of this exercise prescription against a standard programme.

The inclusion of HIIT in our PR programme is likely to help ILD patients increase muscle function, and improve exercise tolerance during everyday activities. It will allow patients to gain more control over their exercise regime. In other respiratory patients, the cumulative effect of HIIT has been shown to be similar to that of continuous programmes [12] or superior [5, 6]. We anticipate that the proposed research will improve physical function, perception of breathlessness and quality of life in ILD patients and that the benefits will be maintained for longer than other rehabilitation programmes. We also expect that self management is likely to improve more with this exercise prescription, as patients will learn to pace their training, and will be encouraged to apply similar approach during functional activities in their everyday life. There is a great need for optimal management of ILD patients, particularly patients with IPF that have greater mortality rate compared to the other types. Our study will improve our knowledge of the efficacy of HIIT in this type of ILD.

The programme will also give us information on the recruitment and retention rates to a HIIT programme per different type of ILD. We will examine the average duration that patients can tolerate HIIT in pulmonary rehabilitation and will obtain means and standard deviations for the change in exercise capacity and other outcomes. This will help us perform sample size calculations when we design a larger, phase III study that will inform guidelines for the rehabilitation of patients in ILD.

Aims and objectives

There are two aims in our study;

The first is to compare the feasibility of high intensity interval training (HIIT) versus moderate intensity continuous exercise in patients with ILD who undergo a pulmonary rehabilitation programme.

For peer review only - http://bmjopen.bmj.com/site/about/guidelines.xhtml

19/11/13- Version 1.0

The second is to study the efficacy of HIIT versus moderate intensity continuous training in improving exercise capacity, breathlessness and quality of life immediately post-pulmonary rehabilitation and long term (i.e. 6 months later).

Specific objectives of interest related to our first aim are:

1. To study the recruitment and retention rates of patients with different types of ILD to each programme
2. To study the adherence of ILD patients to each exercise programme
3. To investigate the average time that high intensity training is tolerated in patients with various types of ILD.

Specific objectives of interest related to our second aim are:

1. To investigate the efficacy of each exercise programme on exercise capacity immediately post-pulmonary rehabilitation and 6 months later
2. To investigate the efficacy of each exercise programme on breathlessness immediately post-pulmonary rehabilitation and 6 months later
3. To study the effects of each programme on quality of life immediately post-pulmonary rehabilitation and 6 months later.

Research Design

Feasibility, randomised, single-blind, controlled study.

Recruitment

Patients with a variety of ILD types will be recruited via the chest clinic at St George's Hospital. The recruitment process will start as soon as ethical approval has been secured for this study and will continue up until the last pulmonary rehabilitation group has been formed (see Gantt chart).

Participants

Participants will be ILD patients, of varying severity and type, who have been referred to pulmonary rehabilitation. Patients with significant musculoskeletal, cardiac or neurological co morbidities that interfere with their ability to exercise will be excluded from the study (standard criteria for all pulmonary rehabilitation programmes). Participants will be free to withdraw at any time. Transport will be provided for all participants who need it.

The research physiotherapists attached to this study will lead the recruitment process. Patients who have agreed to participate will be consented by the lead researcher who will also perform all assessments.

Participants have lung function tests (LFT) and cardiopulmonary exercise testing (CPET) as part of their clinical management. The clinical team records radiographic patterns via high-resolution computer tomography which helps with the stratification of patients into the different ILD types. Data from these clinical tests will be recorded for all participants within 3 months from the beginning of the rehabilitation programmes.

Methods

Sixty ILD patients will be randomised into 2 groups: Group A: Pulmonary rehabilitation (PR) with moderate intensity continuous training (MICT), Group B: Pulmonary rehabilitation with high-intensity interval training (HIIT). The randomization will be stratified by ILD type to ensure a balanced distribution of types between groups. The duration of each programme will be 8 weeks, to reflect current UK practice of pulmonary rehabilitation programmes. Description of the programme is provided further below. In the 2 years of this study, we will run 5 pulmonary rehabilitation programmes in total, each containing 12 ILD participants (6 per arm of the study). The follow up assessments of all key outcomes will be performed immediately post-end of each PR programme and 6 months later (see Gantt chart).

Inclusion/exclusion criteria

Patients included in this study will be:

- Patients with varying severity of ILD (all types apart from Sarcoidosis will be included). The type and severity of participants will be recorded.

For peer review only - http://bmjopen.bmj.com/site/about/guidelines.xhtml

19/11/13- Version 1.0

- Patients whose first language is not English (we will provide interpreters to explain educational presentations and other instructions as per usual clinical practice).

Exclusion criteria will include:

- Patients with sarcoidosis
- Patients with significant co-morbidities (musculoskeletal, cardiac or neurological) that interfere with their ability to exercise.

Data collection

Data will be collected by the lead researcher on 3 occasions: at baseline, post-pulmonary rehabilitation and 6 months after completion of the pulmonary rehabilitation programme.

The following measurements will be taken:

- *Exercise capacity*- We will use the 6min walking test (6MWT) and Cardiopulmonary exercise testing (CPET) on a treadmill (Modified Bruce protocol). The baseline CPET will determine each patient's VO2max which will be used as a guideline for the exercise prescription with HIIT.
- *Breathlessness*- 10-point BORG scale at the beginning and at the end of the exercise tests.
- *Respiratory and peripheral muscle function* –Respiratory: Maximal inspiratory and expiratory pressure (PImax and PEmax) and sniff nasal pressure (SnPnas). Peripheral: Quadriceps muscle strength (MRC scale) and Handgrip strength-assessed via a dynamometer
- *Health status measurements*- St George's Respiratory Questionnaire for ILD (SGRQ-I), CAT-ILD, the Hospital Anxiety and depression scale (HAD).

Structured interviews- All patients who completed will be interviewed. The questions will cover areas of their experience, what they could and could not tolerate in the programme etc.

The primary outcome for each group will be exercise capacity as determined by the 6MWT (meters walked). We chose the 6MWT as our primary outcome as it will allow us to compare our study with other international research exercise programmes in ILD that have used the 6MWT as their outcome measure.

Secondary outcomes will be the change in VO2max from the cardiopulmonary exercise testing, level of breathlessness, respiratory and peripheral muscle function and changes in quality of life.

Randomisation:

Following all baseline assessments, patients will be randomised into the 2 groups (HIIT or MICT) stratifying for ILD type to ensure equal representation of the different types of ILD in each group.

The training programme

The pulmonary rehabilitation (PR) programme is a group programme that consists of an hour of exercise and an hour of education on topics relevant to patients. It runs 2 times per week for 8 weeks (standard UK practice). Home exercises are given for patients to perform on the days that they do not attend the programme. In our PR programme, the one hour of education will be on various topics relevant to ILD such as airway clearance and energy conservation techniques to improve fatigue during daily activities. The hour of exercise will include either MICT or HIIT depending on which group patients were randomised into. The MICT will include aerobic exercises, such as walking and cycle ergometer, and arm and leg exercises at moderate intensity (this will be individualized per patient). The HIIT will include short bursts of high intensity exercises (>80% VO2max) followed by periods of

rest or low intensity exercise. The duration of each burst of exercise and each resting period varies depending on each patient. The exercises will be repeated at regular intervals during the hour. Exercise prescription will be designed for each patient individually depending on limits of desaturation and breathlessness. The intensity of each interval of exercise will be >80% of each patient's VO2max and the aim will be to increase the duration of each interval in the course of the

For peer review only - http://bmjopen.bmj.com/site/about/guidelines.xhtml

programme. There will be 2 PR sessions per week and each patient will be encouraged to continue with daily home exercises for the rest of the week. The PR programme will be delivered by the two research physiotherapists attached to this study. The physiotherapists will not be involved in the research assessments.

The research assessments will be performed by the lead research physiotherapist and measurements will be taken on 3 occasions: at baseline, immediately post-PR programme and 6 months after completion of the programme.

Sample size and Power calculations

This is a feasibility study, so no power calculation will be made. We anticipate that approximately 60 patients will be recruited in this study over the 2 year period which will provide sufficient information to calculate mean change and standard deviation for the key outcomes, to inform future sample size calculations for a Phase III study.

In the year 2011-2012, over 200 patients with ILD were seen in the chest clinic at St George's Hospital. We therefore anticipate that 60 ILD patients would be feasible to participate in this study over a period of 2 years.

Outcomes and analysis

All outcomes (primary and secondary) will be compared at baseline, post-training (8 weeks later) and after 6 months. We will use factorial analysis of variance (ANOVA) to compare the primary and secondary variables between groups and if necessary, we will adjust for confounding variables with a multivariable regression. More specifically, we will perform the following:

1. Comparison of exercise capacity (meters walked in the 6MWT) at baseline, post-PR and at 6 months between groups.
2. Comparison of respiratory muscle function (PImax, PEmax and SnPnas) and peripheral muscle function (handgrip strength and quadriceps) at baseline, post-PR and at 6 months between groups.
3. Comparison of breathlessness (10-point BORG score at the beginning and end of the 6MWT and at the end of the cardiopulmonary exercise test)
4. Comparison of VO2max at baseline, post-PR and at 6 months between groups.
5. Comparison of health-related quality of life scores at baseline, post-PR and at 6 months between groups.

This analysis will allow us to explore which group achieved the most significant improvements in exercise capacity and in the other variables. Therefore, we will be able to estimate the intervention effect for each rehabilitation option.

An intention to treat analysis will be performed. All patients will be included, including those who did not adhere to the programme or refused to participate after having given consent to join the study. We will record the reasons for refusal or non-adherence. This will allow us to gain valuable information about the feasibility of the programme.

Once we have established which exercise training is more likely to achieve long term benefit in patients with ILD, we will take these results forward in a larger, definitive study and will be able to use our estimated intervention effects and 95% CI to inform the new effect size.

References

1. Glaspole I and Goh NSL. Aspects of interstitial lung disease: differentiating between IPF and NSIP. Chronic Resp Disease 2010; 7 (3): 187- 195.

2. Wells AU and Hirani N. BTS Interstitial lung disease guideline: the British Thoracic Society in collaboration with the Thoracic Society of Australia and New Zeland and the Irish Thoracic Society. Thorax 2008; 63 (Suppl V): v1-v58.

3. Holland AE, Hill CJ, Conron M, *et al*. Short term improvement in exercise capacity and symptoms following exercise training in interstitial lung disease. Thorax 2008; 63: 549-554.

For peer review only - http://bmjopen.bmj.com/site/about/guidelines.xhtml

19/11/13- Version 1.0

4. Gloeckl R, Halle M, Kenn K. Differences in feasibility of interval training between patients with end-stage COPD and interstitial lung disease before lung transplantation- a pilot study. Am J Respir Crit Care Med 2012; 185: A2390.
5. Shiraev T and Barclav G. Evidence based exercise- Clinical benefits of high intensity interval training. Aust Fam Physician 2012; 41 (12): 960-962.
6. Wisloff U, Stovlen A, Loennechen JP, et al. Superior cardiovascular effect of aerobic interval training versus moderate continuous training in heart failure patients. Circulation 2007; 115: 3086-3094.
7. Thijs KM, de Boer AG, Vreugdenhil G, et al. Rehabilitation using high-intensity physical training and long-term return to work in cancer survivors. J Occup Rehabil 2012; 22(2): 220-229.
8. Margaritopoulos GA, Romagnoli M, Poletti V, Siafakas NM, Wells AU and Antoniou KM. Recent advances in the pathogenesis and clinical evaluation of pulmonary fibrosis. Eur Resp Rev 2012; 21: 123, 48-56.
9. Raghu G, Collard HR, Egan J, et al. An official ATS/ERS/JRS/ALAT statement: idiopathic pulmonary fibrosis: evidence-based guidelines for diagnosis and management. Am J Respir Crit Care Med 2011; 183: 788–824.
10.Schwarz Marvin I. And King Talmadge E. Interstitial Lung disease. PMPHUSA, 2010; Ed. 5 1-1161.
11.Holland AE and Hill C. Physical Training for interstitial lung disease (review). Cochrane review 2010; 1-28.
12.Vogiatzis I, Gerasimos Terzis, Serafeim Nanas, *et al*. Skeletal muscle adaptations to interval training in patients with advanced COPD. Chest 2005; 128: 3838-3845.
13.Young P, Dewse M, Fergusson W, Kolbe J. Respiratory rehabilitation in chronic obstructive pulmonary disease; Predictors of non-adherence. Eur Respi J 1999; 13 (4): 855-859.
14.Garrod R, Marshal J, Barley E, Jones PW. Predictors of success and failure in pulmonary rehabilitation. Eur Resp J 2006; 27 (4): 788-794.
15. Keating A, Lee A, Holland A. What prevents people with COPD from attending pulmonary rehabilitation? A systematic review. Chronic Resp D 2011; 8: 89-99.

For peer review only

BMJ Open

Comparison of high intensity interval training versus moderate intensity continuous training in pulmonary rehabilitation for interstitial lung disease; a randomised controlled pilot feasibility trial.

Journal:	*BMJ Open*
Manuscript ID	bmjopen-2022-066609.R1
Article Type:	Original research
Date Submitted by the Author:	13-Apr-2023

Complete List of Authors:	Nikoletou, Dimitra; St George's University of London, Centre for Allied Health; Kingston University, Rehabilitation sciences<br>Chis Ster, Irina; St George's University of London, Infection and Immunity Research Institute<br>Lech, Carmen; St George's University of London, Infection and Immunity Research Institute<br>MacNaughton, Iain; St George's University of London, Infection and Immunity Research institute<br>Chua, Felix; Royal Brompton and Harefield NHS Foundation Trust, Interstitial Lung Disease Unit; Imperial College London, National Heart and Lung Institute<br>Aul, Raminder; St George's University Hospitals NHS Foundation Trust, Respiratory Medicine<br>Jones, Paul; St George's University of London, Infection and Immunity Research institute

**Primary Subject Heading**:	Respiratory medicine
Secondary Subject Heading:	Rehabilitation medicine
Keywords:	Interstitial lung disease < THORACIC MEDICINE, REHABILITATION MEDICINE, PREVENTIVE MEDICINE

SCHOLARONE™ Manuscripts

I, the Submitting Author has the right to grant and does grant on behalf of all authors of the Work (as defined in the below author licence), an exclusive licence and/or a non-exclusive licence for contributions from authors who are: i) UK Crown employees; ii) where BMJ has agreed a CC-BY licence shall apply, and/or iii) in accordance with the terms applicable for US Federal Government officers or employees acting as part of their official duties; on a worldwide, perpetual, irrevocable, royalty-free basis to BMJ Publishing Group Ltd ("BMJ") its licensees and where the relevant Journal is co-owned by BMJ to the co-owners of the Journal, to publish the Work in this journal and any other BMJ products and to exploit all rights, as set out in our licence.

The Submitting Author accepts and understands that any supply made under these terms is made by BMJ to the Submitting Author unless you are acting as an employee on behalf of your employer or a postgraduate student of an affiliated institution which is paying any applicable article publishing charge ("APC") for Open Access articles. Where the Submitting Author wishes to make the Work available on an Open Access basis (and intends to pay the relevant APC), the terms of reuse of such Open Access shall be governed by a Creative Commons licence – details of these licences and which Creative Commons licence will apply to this Work are set out in our licence referred to above.

Other than as permitted in any relevant BMJ Author's Self Archiving Policies, I confirm this Work has not been accepted for publication elsewhere, is not being considered for publication elsewhere and does not duplicate material already published. I confirm all authors consent to publication of this Work and authorise the granting of this licence.

For peer review only

Title: Comparison of high intensity interval training versus moderate intensity continuous training in pulmonary rehabilitation for interstitial lung disease; a randomised controlled pilot feasibility trial.

Authors:

Dr Dimitra Nikoletou[1, 2,] PhD dinikole@sgul.ac.uk

Dr Irina Chis-Ster[3], PhD ichisste@sgul.ac.uk

Ms Carmen Y. Lech[3], BSc carmenyanina.lech@gmail.com

Mr Iain S. MacNaughton[3], BSc iainmacn62@gmail.com

Dr Felix Chua MD[4], PhD F.Chua@rbht.nhs.uk

Dr Raminder Aul MD[5] raminder.aul@stgeorges.nhs.uk

Professor Paul Jones[3] MD, PhD pjones@sgul.ac.uk

Authors' affiliations:

1. Centre for Allied Health, St George's University of London, Cranmer Terrace, London, SW17 ORE, UK.
2. Faculty of Health, Science, Social care and Education, Kingston University, London, UK.
3. Infection and Immunity Research Institute, St George's University of London, Cranmer Terrace, London, SW17 ORE, UK
4. Department of Respiratory Medicine, Royal Brompton & Harefield NHS Foundation Trust, London, UK.
5. Department of Respiratory Medicine, St George's Hospital NHS Foundation Trust, London SW17 ORE, UK

Corresponding author:
Dr Dimitra Nikoletou, Centre for Allied Health, St George's University of London, Cranmer Terrace, London, SW17 ORE and (honorary) Faculty of Health, Science, Social care and Education, Kingston University, London, UK.
Tel: 0208 725 2277 Email: dinikole@sgul.ac.uk

Key words: Interstitial lung disease, ILD, Pulmonary rehabilitation, Interstitial pulmonary fibrosis, exercise, high intensity interval training, HIIT.

Running head: Feasibility of HIIT versus MICT in ILD PR programmes

Trial registration: This trial was registered in ISRCTN, with the ID number ISRCTN55846300.

Word count: 3949

For peer review only

Abstract

Objectives: This study aimed to investigate the feasibility and efficacy of high intensity interval training (HIIT) compared to moderate intensity continuous training (MICT) in pulmonary rehabilitation (PR) for people with interstitial lung disease (ILD).

Design: Single-centre, randomised controlled feasibility, pilot trial.

Setting: Patients were recruited from the chest clinic of a tertiary ILD centre and attended circuit-based PR in the hospital's gym, followed by a personalised 6-month community programme.

Participants: 58 patients, stratified per ILD type, were randomised into two groups: 33 to HIIT (18M: 15F) [mean age (SD) 70.2 (11.4) years] and 25 to the MICT exercise mode (14M: 11F), [mean age (SD) 69.8 (10.8) years].

Interventions: 8-week, twice weekly, circuit-based PR programme of exercise and education, followed by a personalised 6-month community exercise programme.

Outcome measures: Feasibility outcomes included staff-to-patient ratio and dropout rates per group. Primary outcome was the 6-minute walk distance (6MWD). Secondary outcomes included the sniff nasal pressure, mouth inspiratory and expiratory pressures, handgrip and quadriceps strength and health status. Random-effects models were used to evaluate average variation in outcomes through time across the two groups.

Results: The 6MWD peaked earlier with HIIT compared to MICT (at 4 months versus 5 months) but values were lower at peak (mean (95%CI) 26.3m (3.5, 49.1) versus 51.6m (29.2, 73.9) and declined faster at 6-months post PR. Secondary outcomes showed similar faster but smaller improvements with HIIT over MICT and more consistent maintenance 6-months post-PR with MICT than HIIT.

Conclusions: HIIT is feasible in circuit-based ILD PR programmes and provides quick improvements but requires closer supervision of training and resources than MICT and benefits may be less well sustained. This would make it a less attractive option for clinical PR programmes. A definitive, multi-centre randomised controlled trial is required to address the role of HIIT in ILD.

For peer review only

Strengths and Limitations of this study

- This randomised controlled feasibility, pilot study, showed that HIIT is feasible in circuit-based PR programmes.
- This study adds valuable information for the design of future, definitive studies, on the peak and subsequent deterioration of outcomes over time depending on exercise mode.
- Our results identify the point at which exercise reinforcement may be needed after completion of a HIIT programme.
- Although it was possible to recruit enough ILD patients from a single site, not enough IPF patients could be recruited to evaluate the effect of HIIT on this particular group.
- We did not use supplementary oxygen during PR for patients other than those already on long-term oxygen therapy.
- Subsequent powered studies should be larger and block-randomise to mitigate against the potential effect of differences in baseline lung function on key outcomes.

For peer review only

Background

For peer review only - http://bmjopen.bmj.com/site/about/guidelines.xhtml

Interstitial lung disease (ILD) is a broad term used to describe a diverse group of pulmonary conditions that cause fibrotic changes in the lung interstitium with subsequent reduction in lung compliance and oxygen diffusion capacity [1, 2]. ILD is categorised into its different types according to clinical, radiological and pathophysiological changes and multidimensional indexes have been created to predict mortality risk [3, 4]. The clinical presentation includes breathlessness on exertion, dry and persistent cough, fatigue, progressive exercise limitation and respiratory failure at the very severe stages. [5] Pharmacological treatment for patients with progressive fibrotic ILD is limited to only a few medications aiming to slow down further development of fibrosis [6] and lung transplantation, the only life-extending option, carries risks and is not available to all patients [7, 8].

Pulmonary rehabilitation (PR) is a non-pharmacological strategy that improves physical function and quality of life [5, 9] but in ILD patients the optimal training programme, especially for longer term benefits, remains unclear. [5, 10] Recent evidence suggests that patients with some types of ILD, such as asbestosis and idiopathic pulmonary fibrosis (IPF), may receive greater benefit from PR than others and effects may be more lasting in patients with milder disease [11]. The traditional exercise mode in PR is moderate intensity continuous training (MICT) which involves continuous exercise for 30-60 minutes at intensities ranging from 60-80% maximum heart rate [12]. However, other training modalities may offer greater benefits.

High intensity interval training (HIIT) is a modality characterised by intervals of high intensity exercise followed by intervals of low intensity or rest [13]. There is growing evidence that HIIT improves a broad range of cardiovascular risk factors, including insulin sensitivity and VO_2peak [14], and its safety has been evaluated in patients with cardiovascular and metabolic disease [14-17]. Respiratory patients, who cannot maintain exercise due to breathlessness, have found HIIT acceptable [18- 20] and although its overall superiority over MICT has been questioned [21], it has been used safely in ILD patients preparing for lung transplantation [22]. However, HIIT has yet to be evaluated in the context of an ILD-tailored PR programme.

For peer review only - http://bmjopen.bmj.com/site/about/guidelines.xhtml

The aim of this pilot study was therefore to evaluate: a) the feasibility of using HIIT in an ILD-PR programme, b) determine the short and medium-term effects of HIIT on exercise capacity, respiratory and peripheral muscle strength, breathlessness and health status and c) explore responses in different types of ILD. Some of the results of this study have been reported previously abstract form [23].

Methods

Study design

Single-blind, randomised-controlled feasibility, pilot trial.

Study population

Symptomatic patients over 18 years old. of all types and severity apart from sarcoidosis and with a respiratory-physician diagnosis of ILD from a tertiary referral hospital, were invited to participate. This included patients with idiopathic pulmonary fibrosis (diagnostic criteria consistent with the International Consensus statement [1]), connective tissue disease related ILD, non-specific interstitial pneumonia, usual interstitial pneumonia, and chronic hypersensitivity pneumonitis. Patients were eligible to participate if they were ambulant and had symptoms of dyspnoea on exertion (grades 1 to 3 in the mMRC scale) [24]. Patients on long-term oxygen therapy (LTOT) were also included. Exclusion criteria were a history of syncope on exertion, patients with sarcoidosis and severe co-morbidities (such as musculoskeletal, neurological or cardiovascular problems) that would interfere with their ability to exercise.

All participants provided written informed consent. The trial protocol was pre-registered with the ISRCTN registry (ISRCTN55846300) and approved by the South East Coast-Surrey Ethics committee (14/LO/0149).

For peer review only

Patient and Public involvement

People with ILD were involved in the original design of this research and determined acceptability of the proposed interventions and likelihood of adherence to this programme. During the study, all participants were involved in the conduct of the research by giving regular feedback in the education-discussion part of the PR programme and via interviews. On completion of this study, some participants formed a local ILD-Support Group and viewed the final results for dissemination.

Randomisation and blinding

Following baseline assessments, participants were randomised into 2 groups: exercise using moderate intensity continuous training (MICT group) or exercise using high-intensity interval training (HIIT group). Participants were stratified by ILD type to ensure a balanced distribution of types between groups. They were placed into 3 sub-groups: a) Idiopathic group (e.g. IPF); b) Autoimmune group (CTD-ILD) and c) Chronic Hypersensitivity pneumonitis group (CHP/EAA). The investigator performing all assessments and the statistician were blinded throughout the study.

Intervention

The ILD-PR programme

This was a circuit-based 8-week, twice weekly, outpatient programme, including only people with ILD and consisting of an hour of exercise and an hour of education. Exercise was set, supervised and progressed by two experienced physiotherapists, according to a standardised protocol for each group allocation. Aerobic exercises included treadmill walking, brisk walking between 2 cones, cycle ergometer, trampette and step ups. Strength training exercises, upper and lower limb, were conducted using 'Thera-bands' and functional movements such as sit-to-stand and ball raises.

For peer review only - http://bmjopen.bmj.com/site/about/guidelines.xhtml

A minimum of 30 minutes were dedicated to aerobic training. Aerobic training intensity at the MICT group was set at 60% maximum heart rate (HRmax) and progressed each week. In the HIIT group, intensity was set at 80% HRmax and patients were instructed to exercise in dynamic intervals followed by low intensity exercise or rest. The aim in this group was to increase the amount of time on the 'high intensity' phase each week.

In the first week, the average time on HIIT (at 80% HRmax) for each aerobic exercise was 2.5 min, therefore HIIT was 12.5 minutes of the total 30-minute session, and this time increased each week. Progression of training was determined by reducing time in the low intensity phase of HIIT and/or increasing time in the target HR . Each participant's individualised programme was recorded on exercise logs during the sessions.

Heart rate and oxygen saturation were monitored using pulse oximeters and perceived exertion and breathlessness were recorded at set intervals using the modified 10-point Borg scale and the Rate of Perceived Exertion scale (RPE) respectively [25, 26].

The education session, on the 2nd hour of the programme was common for all participants and included presentations and discussion on topics important to ILD patients, such as: Topics about breathing and breathlessness (mechanics of breathing, helpful/unhelpful breathing patterns, the Active Cycle of Breathing technique, useful positions for recovering, pacing), topics about ILD (what happens with ILD, cough, associated conditions-rheumatoid Arthritis, Gastro-Oesophageal Reflux disease, Sjögren's syndrome, Bird Fancier's Lung, Idiopathic Pulmonary Fibrosis) and topics about managing the condition (Why healthier lifestyle is important, keeping good posture, barriers to activity/exercise, dietary advice, smoking, incontinence, relaxation information, mood, anxiety and breathlessness, self-management and goal setting).

For peer review only - http://bmjopen.bmj.com/site/about/guidelines.xhtml

On the first day of the programme all participants were given an 'ILD-Pulmonary rehabilitation booklet' designed by our team, which contained information and pictures of all exercises, an exercise diary and summary of the education topics. All participants were encouraged to continue exercising at home on the days they did not attend the programme.

The 6-month programme

Following completion of the PR programme, participants commenced a personalised community exercise programme, starting at the level they achieved at the end of PR. The 6-month programme included outdoor walking in HIIT or MICT mode, as well as home-based stretching and strength training exercises. Progress was monitored by phone calls (once per month) and at least one home visit by the research physiotherapists on the 3rd month. The physiotherapists guided participants on filling in the exercise diaries and progressing training depending on mode of exercise. The ILD-PR booklet was used as a resource for the home exercises and as a reminder of the educational topics.

Outcomes

All assessments were performed by the same investigator (DN), using identical instructions and methods, at three time points; at baseline, at 8-weeks (post-PR) and at 6 months (post-community programme). The investigator was blinded throughout the study.

Feasibility outcomes included the proportion of eligible patients who consented to the study, attrition in each group, staff-to-patient ratio and supervision requirements, reasons for discontinuing the programme and adverse events.

The primary efficacy outcome was functional exercise capacity and was measured using the 6-minute walk test distance (6MWD) as recommended by the ATS guidelines [27] using standardised instructions.

For peer review only - http://bmjopen.bmj.com/site/about/guidelines.xhtml

For peer review only

Secondary outcomes were: a) Respiratory muscle function- Sniff nasal pressure (SNIP) and the maximal inspiratory and expiratory pressures (PImax and PEmax) were recorded using a handheld portable respiratory muscle testing device (MicroRPM, CareFusion, Basingstoke, UK). A minimum of 10 attempts were made for each test, on at least one session. PImax was recorded from functional residual capacity (FRC) and PEmax from total lung capacity (TLC).

b) Peripheral muscle function- Handgrip strength, assessed in the dominant hand, with the elbow at 90 degrees angle, using a hydraulic hand dynamometer (JAMAR Hydraulic Hand Dynamometer, Homecraft Rolyan Ltd, Nottinghamshire, UK). Quadriceps strength (QUADS-DOM) and hip flexion (HIP-DOM) were assessed in sitting position, with the knee at 90 degrees angle and back upright and supported using a hand-held dynamometer (Lafayette Manual Muscle Tester Model 01163, Lafayette Instruments, Lafayette, IN, USA).

c) Health status, using the St George's Respiratory Questionnaire for IPF (SGRQ-I) [28] and the Hospital Anxiety and Depression scale (HAD) [29].

Spirometry data and anthropometric measurements (height, weight, %fat, waist, hip and neck circumference) were also collected.

Data were collected and managed using REDCap electronic data capture tools hosted at the NHS Trust [30].

Statistical analysis

This was a pilot study to test feasibility and inform the design and power of a larger, definitive, randomised controlled trial. An intention to treat analysis was used. An indicative sample size calculation was based on previous studies aiming to detect between-group changes of 38± 43m with an 80% power at p <.05 significance level in the 6MWD following rehabilitation [31-33].

Random effects models were used to estimate average values in outcomes and predictions were derived following a nonlinear, quadratic model for changes over time and a 3-way interaction between time, intervention (HIIT vs MICT) and clinical group (ILD type) as well as between age, intervention and clinical

For peer review only - http://bmjopen.bmj.com/site/about/guidelines.xhtml

group. For each of the 4 key outcomes of interest (6MWD, SNIP, SGRQ-I and Quads strength), a series of nested models of increasing complexity was developed and tested for their statistical significance against the next less complex model. The average variation of outcomes through time, within and between-groups, are presented as means (95% CI) and descriptive statistics as mean (SD). For the plots of change over time, patients were grouped into tertiles. Patterns in the missing data have been assessed and the mixed modelling operated under missing at random assumption. Sensitivity analyses to the missing data have been conducted and the results did not show important departures from inferences based on complete data (analyses not shown). All analyses were conducted on STATA software (version 11).

Results

Patient flow

60 participants consented to the study but two participants withdrew prior to commencement of the PR programme. A total of 58 started the PR programme. Figure 1 is the CONSORT diagram showing participant flow.

Figure 1 around here

Baseline characteristics are shown in Table 1 . There were no differences between groups at baseline, except for DLCO which was significantly lower in the HIIT group.

For peer review only - http://bmjopen.bmj.com/site/about/guidelines.xhtml

Table 1: Baseline characteristics

Variable	Total Group (n= 58)	Control group (MICT) (n=25)	Intervention group (HIIT) (n=33)	Between group P value
**Age (years)**	70.0 (11.05)	69.8 (10.8)	70.5 (10.9)	0.89
**Gender (male), (number (%))**	32 (55.2)	14 (56)	18 (55)	0.91
**ILD Type, (no. (%))**				
• IPF	18 (31.0)	8 (32)	10 (30.3)	0.10
• CTD-ILD	28 (48.3)	15 (60)	13 (39.4)	
• CHP	12 (20.7)	2 (8)	10 (30.3)	
**ILD-GAP index,** (number (%))				
• 0-1	24 (41.4)	12 (48)	12 (48)	0.72§
• 2-3	20 (34.5)	9 (36)	11 (33.3)	
• 4-5	10 (17.2)	4 (16)	6 (18.2)	
• >5	2 (3.4)	0 (0)	2 (6.1)	
**BMI (kg/m^2)**	27.3 (4.6)	27.5 (3.8)	27.2 (5.1)	0.84
**Waist/Hip ratio**	0.9 (0.1)	0.9 (0.1)	0.9 (0.1)	0.60
**Fat (%)**	29.9 (8.6)	29.9 (9.3)	30.1 (8.1)	0.95
**FVC (%,predicted)**	79.1 (21.8)	85.8 (24.0)	73.8 (18.8)	0.11
**DLCO (%, predicted)**	47.0 (14.7)	53.9 (13.1)	41.7 (13.9)	0.002
**Comorbidities (no. (%))**				
• Cardiac	21 (36.2)	8 (32)	13 (39.4)	0.56
• Hypertension	23 (39.7)	9 (36)	14 (42.4)	0.62
• Diabetes	15 (25.9)	7 (28)	8 (24.2)	0.75
• High Cholesterol	12 (20.7)	7 (28)	5 (15.2)	0.23

For peer review only - http://bmjopen.bmj.com/site/about/guidelines.xhtml

**Prednisolone medication**<br><br>**(no. (%))**<br>• < 5mg<br>• 5-10 mg<br>• >10 mg	19 (32.8)<br><br>19 (32.8)<br><br>3 (5.17)	9 (36)<br><br>5 (20)<br><br>2 (8)	10 (30.3)<br><br>14 (42.4)<br><br>1 (3)	0.34§
**Smoking status (no. (%))**<br>• Current smoker<br>• Former smoker<br>• Never smoked<br>• Missing records	13 (22.4)<br><br>9 (15.5)<br><br>33 (56.9)<br><br>3	5 (20)<br><br>6 (24)<br><br>13 (52)<br><br>1	8 (24.2)<br><br>3 (9.1)<br><br>20 (60.6)<br><br>2	0.31§
**Pack year history**	8.9 (15.9)	10.7 (16.5)	7.4 (15.6)	0.50
Median/IQR interval	0(0, 10)	0(0, 20)	0(0, 4.5)	
Missing	7	3	5	
**6MWD (m)**	371.7 (122.2)	380.8 (139.8)	364.6 (108.3)	0.65
6MWD Missing	6	2	4	
**SpO2 in room air (%)**	96 (1.5)	96.5 (1.8)	96 (1.5)	0.3
Missing	6	2	4	
**No. on LTOT**	3	1	2	n/a
**SNIP (cmH_2O)**	94.8 (24.3)	96.8 (25.9)	93.1 (24.5)	0.61
Missing	9	3	6	
**PImax (cmH_2O)**	91.5 (30.4)	91.1 (34.2)	91.8 (27.4)	
Missing	8	2	6	
**PEmax (cmH_2O)**	106.6 (32.0)	114.4 (40.3)	100.7 (23.5)	0.22
Missing	23	10	13	
**Handgrip (dominant hand) (kg)**	25.8 (10.2)	27.2 (11.2)	24.8 (9.4)	0.42
Handgrip Missing	4	2	2	
**Quads extension (dominant side) (kg)**	18.3 (5.5)	18.7 (6.2)	18 (5.0)	0.65
Missing	2	0	2	
**Hip flexion (dominant side) (kg)**	15.6 (4.6)	15.3 (5.1)	15.9 (4.3)	0.41

For peer review only - http://bmjopen.bmj.com/site/about/guidelines.xhtml

Missing	1	0	1	
**HAD-A**	6.3 (4.2)	6.2 (4.6)	6.3 (3.9)	0.88
Missing	1	0	1	
**HAD-D**	5.5 (3.7)	5.8 (3.2)	5.2 (4.0)	0.28
Missing	1	0	1	
**SGRQ-I Total score**	46.6 (21.2)	43.3 (20.3)	49.3 (21.8)	0.29
Missing	1	0	1	

Data are means (SD) unless otherwise stated. The p-value tests the general null hypothesis of similarity between the randomized groups. The methods include t-tests (equal or unequal variances), Kruskal- Wallis and chi-squared (Fisher exact) as appropriate according to the nature of the variables. All tests have been carried out on the complete data only and missing data were not considered as a separate category. §= p value was calculated considering the whole (balanced) distribution.

Feasibility of HIIT compared to MICT

HIIT was well tolerated in a circuit-based clinical PR programme but required closer supervision to monitor participants' target heart rate and avoid desaturation below 85%. There were no adverse events from HIIT during or after the PR programme although attrition in the HIIT group was higher (42% compared to 28% in the MICT group) this did not appear to be due to a specific factor that could be directly attributed to the type of programme (Figure 1).

Change in exercise capacity, respiratory and peripheral muscle strength and health status

The change in outcomes from baseline at all assessment points are shown in Figure 2 and suggest a non-linear trend. This was then tested using linear and non-linear models and it was found that a quadratic model provided a significantly better fit than a linear model. The 6MWD increased over time from baseline in both groups, after which it tended to return towards starting levels ($p<0.001$ for a quadratic term of change over time), Figure 3. The p value for the group-time interaction term was $p=0.006$ suggesting that the trajectories differed between groups. The HIIT group peaked quicker, around 4 months compared to

For peer review only - http://bmjopen.bmj.com/site/about/guidelines.xhtml

For peer review only

around 5 months with MICT (Figure 3). The models also suggested that the change in 6MWD values were smaller with HIIT (Table 2, Figure 2); mean change at peak was 51.6m (95% CI 29.2, 73.9) in the MICT group compared to 26.3m (3.5, 49.1) with HIIT. The picture was the same using the estimates at 2 and 8 months (Table 2, Figure 2). Age was inversely associated with the average 6MWD values in both groups, but the time course was similar across ages (Fig. 3).

Figure 2 and 3 around here

For peer review only

Table 2: Average predictions for main outcomes values at the main time points during the trial and for changes from baseline (actual and adjusted for age)

For peer review only - http://bmjopen.bmj.com/site/about/guidelines.xhtml

Outcome	Time	Group data Mean (95% CI)		Change from baseline Mean (95% CI)		Change from baseline adjusted for age Mean (95% CI)	
		**Control (MICT)**	**Intervention (HIIT)**	**Control (MICT)**	**Intervention (HIIT)**	**Control (MICT)**	**Intervention (HIIT)**
**6MWD (m)**	**Baseline**	382 (336, 429)	372 (331, 412)				
	**2 months**	433 (386 480)	411 (370, 453)	54.7 (32., 76.9)	38.6 (15.95, 61.17)	53.2 (33.66, 72.70)	36.3 (16.42, 56.06)
	**8 months**	428 (378, 476)	374 (331, 418)	48.2 (24.0, 72.4)	2.64 (-23.4, 28.6)	46.6 (26.5, 66.7)	.06 (-21.95, 22.08)
	**Peak value**	460 (411, 508)	425 (382, 468)	51.6 (29.2, 73.9)	26.29 (3.5, 49.1)	50.02 (31.2, 68.8)	23.88 (4.5, 43.3)
	**Peak time-months**	4.87 (4.43, 5.30)	4.05 (3.62, 4.48)				
**SNIP (cmH2O)**	**Baseline**	95 (85, 105)	92 (82, 101)				
	**2 months**	104 (94, 115)	98 (88, 108	9.53 (2.02, 17.04)	10.07 (2.28, 17.85)	9.21 (1.98, 16.4)	9.40 (1.88, 16.92)
	**8 months**	101 (90, 112)	86.9 (76, 98)	8.08 (-0.32, 16.48)	-5.73 (-15.03, 3.56)	7.38 -1.05774 15.81	-6.35 -15.64376 2.95
	**Peak value**	108 (97, 120)	99 (89, 110)	8.89 (2.33, 15.44)	6.01 (-1.10, 13.12)	8.39 (2.00, 14.78)	5.36 (-1.53, 12.25)
	**Peak time-months**	4.57 (3.72, 5.43)	3.54 (2.57, 4.51)				
**QU**	**Baseline**	18.8 (16.7, 20.9)	17.6 (15.7, 19.5)				
	**2 months**	21.6 (19.4, 23.8)	20.1 (18.1, 22.1)	3.37 (1.97, 4.78)	2.44 (1.09, 3.78)	3.33 (1.91, 4.74)	2.39 (1.03, 3.74)

For peer review only - http://bmjopen.bmj.com/site/about/guidelines.xhtml

	**8 months**	22.7<br>(20.2, 25.6)	20.6<br>(18.2, 23.0)	4.10<br>(2.48, 5.72)	3.16<br>(1.55, 4.78)	4.06<br>(2.46, 5.65)	3.12<br>(1.52, 4.71)
	**Peak value**	23.5<br>(21.0, 26.0)	21.7<br>(19.3, 24.1)	3.82<br>(2.46, 5.17)	2.83<br>(1.53, 4.13)	3.77<br>(2.43, 5.11)	2.78<br>(1.49, 4.07)
	**Peak time-months**	5.65<br>(4.39,6.91)	5.27<br>(4.19, 6.36)				
**SGRQ-I (Total score)**	**Baseline**	43.2<br>(35.4, 51.1)	48.8<br>(41.9, 55.7)				
	**2 months**	36.6<br>(28.5, 44.7)	43.0<br>(35.8, 50.2)	-6.57<br>(-11.81, -1.33)	-3.75<br>(-8.92, 1.42)	-6.39<br>(-11.62, -1.17)	-3.54<br>(-8.71, 1.63)
	**8 months**	40.3<br>(31.8, 48.8)	49.2<br>(41.3, 57.1)	-2.57<br>(-8.06, 2.92)	2.15<br>(-3.39, 7.68)	-2.40<br>(-7.89, 3.09)	2.37<br>(-3.18, 7.93)
	**Min value**	33.9<br>(25.1, 42.6)	41.1<br>(33.1, 49.1)	-4.99<br>(-9.62, -0.36)	-1.84<br>(-6.51, 2.83)	-4.82<br>(-9.44, -0.19)	-1.63<br>(-6.31, 3.05)
	**Min time-months**	4.37<br>(3.68, 5.07)	3.94<br>(3.24, 4.65)				

The quadratic term was significant for all secondary outcomes (p=0.0014 for SNIP, p= 0.004 for QUADS-DOM and p= 0.0014 for SGRQ-I), (Figure 3).

SNIP values peaked earlier than 6MWD values in both groups by approximately 2 weeks (Figure 3). QUADS-DOM values were maintained above baseline levels in both groups (Figure 2) and showed a similar time course of change.

There was no evidence for a difference between treatment groups for the SGRQ-I values. The time to reaching the greatest improvement in SGRQ-I score was shorter in the HIIT group, but the changes in the HIIT group were of smaller magnitude at all time-points (Supplementary Tables 1 and 2).

For peer review only

Effects of HIIT on different aetiology ILD

Our models included 3-way interactions between time, intervention (HIIT or MICT) and clinical group, to predict changes from baseline in the combined fibrosis (IPF and CHP group) and CTD-ILD groups. The patterns of change are consistent with the primary analysis – i.e. that whilst the HIIT group peaked faster than the MICT group, the level of improvement was less (Supplementary Tables 1 and 2 and Supplementary Figures 1-4).

The effect of baseline DLCO on HIIT

Despite stratified randomisation, DLCO seemed to differ between intervention groups. (Table 1). We therefore performed additional, post-hoc analyses to examine its possible effect on outcomes. Based on these data, there was no evidence to suggest that DLCO further explained differences in the outcomes after accounting for time, age at the recruitment, intervention and clinical groups and their interactions (see Supplementary file).

Discussion

To our knowledge, this is the first study to investigate the feasibility and potential efficacy of HIIT in the context of a circuit-based clinical PR programme for ILD and compare it with MICT. It demonstrated the feasibility of HIIT, in PR for ILD patients and provided preliminary data on the exercise mode more likely to maintain benefits 6 months after programme completion. In addition, we identified the time of peak benefit and time course of subsequent deterioration.

HIIT proved to be feasible and well tolerated but required closer supervision and careful instructions for patients to continue this mode of training than MICT. The attrition rate was higher in the HIIT group

For peer review only - http://bmjopen.bmj.com/site/about/guidelines.xhtml

compared to the MICT group and more contact was needed in the 6-month period to ensure the HIIT mode of training met the protocol requirements.

The preliminary efficacy of HIIT over MICT was tested using random effects models. Their aim was to understand the average variation of the four key outcomes through time across groups to answer five main questions: a) How do these measurements change over time?; b) Does the benefit differ between groups?; c) How sustainable is the benefit? d) Does the attrition rate differ between groups? e) What is the influence of age? The study was primarily designed to compare the two treatment groups, but an exploratory analysis was performed to look for differences between clinical groups, although this resulted in small numbers in the subgroups.

Contrary to our initial hypothesis, our data showed no evidence of greater benefit with HIIT compared to that achieved with MICT in exercise capacity, respiratory and peripheral muscle function or health status in the medium-term. The study was designed as a pilot to test the potential for a trial of HIIT in a larger, definitive study, but we are forced to conclude that a study of the same design may not be worthwhile.

At 2 months, both groups improved > 34 meters in the 6MWD, which is the reported minimal clinical important difference (MCID) in IPF patients [31, 34, 35], adding to existing evidence on the benefits of PR in ILD [11, 31-32, 36-40]. However, although the 6MWD improved earlier in the HIIT group (peaking at 4 months instead of 5 months) differences from baseline were consistently lower and not as well maintained at 8 months compared with the MICT group. A similar pattern emerged when the effect of age was added to the model. Age is an important factor in indices associated with mortality risk in ILD [3, 4] and our results showed lower 6MWD with greater age. Despite this age effect, PR had a positive effect in all age groups regardless of baseline levels, contrary to previous studies reporting greater improvement in patients with lower baseline 6MWD [11, 35, 39, 41]. However, HIIT did not offer additional or preferential benefits to any specific age group and tended to decline below baseline levels at 8 months in the older group. In contrast, the benefit seen in the older MICT group was maintained above baseline levels.

For peer review only - http://bmjopen.bmj.com/site/about/guidelines.xhtml

In addition to functional exercise capacity, patients improved quadriceps muscle strength post-PR but similarly, there was no difference between treatment groups. Age-adjusted results for quadriceps force were less variable than for 6MWD and benefits were well maintained above baseline in both groups. The modelling suggested that quadriceps force peaked a little later than other outcomes at 5.5 months in the MICT group and at 5 months at the HIIT group. Our results support the inclusion of strength training in ILD-PR programmes and suggest that it may explain why some studies showed greater improvement and maintenance in functional capacity [32] while others that focused on endurance training did not [11]. The time course of peak improvement and subsequent decline in quadriceps strength did not follow the pattern seen with 6MWD, in either group. Quadriceps strength has been shown to deteriorate in line with ageing and severity in other chronic respiratory disorders [42] and has been identified as an important predictor of mortality in COPD [43]. Skeletal muscle atrophy has also been identified in advanced ILD [44]. Our ILD group, which included people in advanced stage (as defined by the ILD-GAP score > 5, Table 1) showed improvement in mean quadriceps force post-PR in both groups and across all age groups. We found no evidence that older individuals benefited more from PR.

Respiratory muscle strength was preserved at baseline (mean SNIP, MIP and MEP > 70 cmH2O in both groups) as previous described [45, 46] and improved post-PR in both groups even though there was no inclusion of specific inspiratory muscle training in our programme. In addition, the SNIP followed a similar pattern over time to 6MWD and peaked approximately two weeks earlier than the 6MWD in both the HIIT and MICT group. Although there was no additional benefit with HIIT, our results suggest that SNIP may be more useful in monitoring changes following PR than quadriceps force in ILD patients. In support of this conclusion, Mendoza and colleagues [45] showed that inspiratory muscle strength correlated better with 6MWD than quadriceps twitch force in patients with fibrotic idiopathic interstitial pneumonia. Although our ILD group was more mixed and included CTD-ILD patients, our results confirm that respiratory muscle strength appears preserved in ILD and that inspiratory muscle strength correlates well with the 6MWD. Our

For peer review only - http://bmjopen.bmj.com/site/about/guidelines.xhtml

study is the first to show that inspiratory muscle strength changes in tandem with the 6MWD following PR, regardless of the chosen mode of training.

Health status, as defined by the SGRQ-I and HAD scale improved post-PR and was maintained at 8 months, but again, there was no evidence of superiority of HIIT over the MICT group (Table 1- online supplement).

Effects of HIIT on different aetiology ILD

Random effect models were also created based on ILD type (clinical subgroups). We combined the IPF and CHP subgroups into the combined 'Fibrosis' clinical group. This was due to smaller numbers of IPF participants at the end of the study which did not allow for independent evaluation of this subgroup.

The effects of HIIT on ILD of different aetiology were consistent with our primary analysis, i.e. that HIIT peaked faster and the level of improvement was less. This result seemed to be largely attributable to the fibrosis group, which peaked fastest but showed the lowest improvement from baseline compared to the CTD-ILD group. Similar findings were observed for the other outcomes, again showing lack of preferential benefit from HIIT in any subgroup. Whilst the CTD-ILD subgroup improved in most variables, the HIIT mode was not superior to MICT.

Limitations

The study has limitations. First, although our retrospective analysis showed no evidence that baseline DLCO had an effect on outcomes following our training programme (Supplementary file), the effect may have been concealed by the size of this pilot study. However, DLCO did not affect PR benefits in a previous study [47], but future definitive studies may need to consider controlling this factor at randomisation.

Second, we did not use supplementary oxygen during PR for participants other than for those already on long term oxygen therapy (LTOT). Although promising benefits have been reported with acute oxygen supplementation during exercise in IPF patients [48], there is still insufficient evidence about medium to long-term benefits in ILD patients [33] For our study, we felt it was important to train participants to

For peer review only - http://bmjopen.bmj.com/site/about/guidelines.xhtml

recognise the time it took before desaturation occurred, so that they were able to continue that pattern of training and monitor themselves when unsupervised in community. Participants already on LTOT continued to exercise with oxygen. However, none of the three participants on LTOT finished PR, regardless of group allocation, supporting previous reports that PR may not be appropriate for them [49]. Future studies need to explore different models of training for these cases, such as home-based programmes. Finally, the number of IPF participants who remained in the study was small and did not allow us to investigate the effect of HIIT over time in this particular group.

Implications for future definitive studies

This pilot, enabled us to explore the effect of mode of training on an ILD-PR programme of 8-weeks duration followed by community-based training for 6 months. Our programme duration reflects current UK-based clinical programmes and showed improvements that were maintained at 8 months and were similar to a recent study of a much longer PR programme duration [32]. Although we conclude that there is no evidence to support the use of HIIT in a future definitive study of similar design to this pilot, our results suggest that HIIT may have a role when quicker improvements are required, such as in pre-operative rehabilitation for ILD and that HIIT is feasible to be investigated further in a larger, definitive study of different design. Our results also provide valuable information on the time point where 6MWD and other variables start to deteriorate post-PR and therefore, an estimate of time when additional PR support may be needed, thus optimising PR provision for ILD patients.

Conclusions

HIIT is feasible and improves exercise capacity but this exercise modality showed no evidence of superiority over MICT in a study design that reflected clinical ILD-PR programmes. Indeed, our findings suggest that it

For peer review only - http://bmjopen.bmj.com/site/about/guidelines.xhtml

may be less effective and may require closer monitoring to avoid exercise-induced desaturation so, until further evidence becomes available, MICT appears to be more appropriate in the general clinical setting.

Author Contributions: Concept and Design of Study: DN, PJ, FC, RA, IC-S; Acquisition of Data: DN, CL, IMcN ; Analysis of Data: DN, IC-S; Drafting of Manuscript: DN, IC-S, PJ ; Revision of manuscript critically for important intellectual content: All authors; Approval of final manuscript: All authors

Competing interests: In the last 36 months:

DN, IMN and CYL declare no financial interests.

ICS- has had grants from NIHR, Astra-Zeneca and BHF and is a statistical expert for the Health Research Authority

FC- has received speaker fees and financial sponsorship for conducting meetings by Boehringer-Ingelheim and Roche

RA- has received lecture fees from Pfizer and Novartis

PJ- owns GlaxoSmithKline shares

Ethics statement: Ethical approval was secured through the South East Coast-Surrey NRES committee (REC reference: 14/LO/0149) on the 14th February 2014.

Funding statement: The study was funded by the National Institute for Health Research (NIHR) under its Research for Patient Benefit (RfPB) Programme (Grant Reference Number PB-PG-1112-29067). The views expressed are those of the authors and not necessarily those of the NHS, the NIHR or the Department of Health. The primary trial sponsor was St George's Hospital NHS Foundation Trust (Cranmer Terrace, London, SW17 ORE).

Study registration and timeline: The trial protocol was pre-registered with the ISRCTN registry (ISRCTN55846300). Study duration: 02/05/2014 to 30/09/2016 (last participant assessment), Analysis completed: 2017.

Data sharing statement: Data are available upon reasonable request. All relevant data are presented in this paper; and more information can be provided upon reasonable request from the corresponding author

Acknowledgements: The authors would like to thank all ILD patients and particularly the St George's Interstitial Lung Disease Support Group for their participation and input in this study.

References

For peer review only - http://bmjopen.bmj.com/site/about/guidelines.xhtml

1. Travis WD, Costabel U, Hansell DM, et al. ATS/ERS Committee on Idiopathic Interstitial Pneumonias. An official American Thoracic Society/European Respiratory Society statement: Update of the international multidisciplinary classification of the idiopathic interstitial pneumonias. Am J Respir Crit Care Med 2013 Sep 15; 188 (6): 733-48.
2. Wells AU and Hirani N. BTS Interstitial lung disease guideline: the British Thoracic Society in collaboration with the Thoracic Society of Australia and New Zealand and the Irish Thoracic Society. Thorax 2008; 63 (Suppl V): v1- v58.
3. Ley B, Ryerson CJ, Vittinghoff E, et al. A multidimensional index and staging system for idiopathic pulmonary fibrosis. Ann Intern Med. 2012; 156 (10): 684-91.
4. Ryerson CJ, Vittinghoff E, Ley B, et al. Predicting survival across chronic Interstitial Lung Disease- The ILD-GAP model. Chest 2014; 145 (4): 723-28.
5. Dowman L, Hill CJ, May A, Holland AE. Pulmonary rehabilitation for interstitial lung disease. Cochrane Database Syst Rev. 2021 J; 2 (2):CD006322.
6. Cottin V, Wollin L, Fisher A, et al. Fibrosing interstitial lung diseases: knowns and unknowns. Eur Respir Rev 2019; 28: 180100 [https://doi.org/10.1183/16000617.0100-2018].
7. Leong S W, Bos S, Lordan J L, et al. Lung transplantation for interstitial lung disease: evolution over three decades. BMJ Open Resp Res 2023; 10: e001387. doi:10.1136/bmjresp-2022-001387.
8. Kapnadak SG, Raghu G. Lung transplantation for interstitial lung disease. Eur Respir Rev 2021; 30: 210017 [DOI: 10.1183/16000617.0017-2021]
9. Spruit M. A., Singh S., Garvey C, et al. An Official American Thoracic Society/European Respiratory Society statement: Key concepts and advances in Pulmonary Rehabilitation. Am J Resp Crit Care Med 2013; Vol 188 (no 8): e13-e64.
10. Holland AE, Hill CJ, Conron M, *et al*. Short term improvement in exercise capacity and symptoms following exercise training in interstitial lung disease. Thorax 2008; 63: 549-554.
11. Dowman L. M, McDonald C.F., Hill C.J, *et al.* The evidence of benefits of exercise training in interstitial lung disease: a randomised controlled trial. Thorax 2017; 0: 1-10.
12. American College of Sports Medicine (ACSM)Guidelines for exercise testing and prescription. (2022) 11th edition. Wolters Kluwer Health
13. Helgerud J, Høydal K, Wang E, Karlsen T, Berg P, Bjerkaas M, et al. Aerobic high-intensity intervals improve VO_2max more than moderate training. Med Sci Sports Exerc 2007; 39 (4):665–71.
14. Levinger et al. What Doesn't Kill You Makes You Fitter: A Systematic Review of High-Intensity Interval Exercise for Patients with Cardiovascular and Metabolic Diseases. *Clinical Medicine Insights: Cardiology* 2015:9 53–63.
15. Arena R, Myers J, Forman DE, Lavie CJ, Guazzi M. Should high-intensity-aerobic interval training become the clinical standard in heart failure? *Heart Fail Rev*. 2013; 18 (1): 95–105.
16. Shiraev T and Barclav G. Evidence based exercise- Clinical benefits of high intensity interval training. Aust Fam Physician 2012; 41 (12): 960-962.
17. Wisloff U, Stovlen A, Loennechen JP, et al. Superior cardiovascular effect of aerobic interval training versus moderate continuous training in heart failure patients. Circulation 2007; 115: 3086-3094.
18. Vogiatzis I, Gerasimos Terzis, Serafeim Nanas, *et al*. Skeletal muscle adaptations to interval training in patients with advanced COPD. Chest 2005; 128: 3838-3845.
19. Vogiatzis I, Nanas S, Kastanakis E, et al. Dynamic hyperinflation and tolerance to interval exercise in patients with advanced COPD. Eur Respir J 2004; 24: 385–390.

For peer review only - http://bmjopen.bmj.com/site/about/guidelines.xhtml

20. Kortianou EA, Nasis IG, Spetsioti ST, et al. Effectiveness of interval exercise training in patients with COPD. Cardiopulm Phys Ther J 2010; 21: 12–19.
21. Beauchamp M. A, Nonoyama M., Goldstein R. S., et al. Interval versus continuous training in individuals with chronic obstructive pulmonary disease- a systematic review. Thorax 2010; 65: 157-164.
22. Gloeckl R, Halle M, Kenn K. Interval versus continuous training in lung transplant candidates: a randomized trial. J Heart Lung Transplant 2012; 31: 934–941.
23. Nikoletou D, Lech C. Y., MacNaughton I.S, Chis-Ster I., Chua F., Aul R., Jones P.. High intensity interval versus moderate intensity continuous training in pulmonary rehabilitation for patients with interstitial lung disease. - American Thoracic Society Conference- B109. HIGHLIGHTS AND ADVANCES IN PULMONARY REHABILITATION. May 2016, A4520-A4520 San Francisco, USA.
24. Manali, E.D., Lyberopoulos, P., Triantafillidou, C. *et al.* MRC chronic Dyspnea Scale: Relationships with cardiopulmonary exercise testing and 6-minute walk test in idiopathic pulmonary fibrosis patients: a prospective study. *BMC Pulm Med* **10**, 32 (2010). https://doi.org/10.1186/1471-2466-10-32
25. Borg G: Perceived exertion as an indicator of somatic stress. Scand J Rehabil Med. 1970, 2: 92-98.
26. Borg GA: Psychophysical bases of perceived exertion. Med Sci Sports Exerc. 1982, 14: 377-381
27. ATS statement: guidelines for the six-minute walk test. Am J Respir Crit Care Med 2002; 166: 111–17.
28. Yorke J, Jones P, Swigris J J. Development and validity testing of an IPF-specific version of the St George's Respiratory questionnaire. Thorax 2010; 65: 921-926.
29. Zigmond AS and Snaith RP: The Hospital Anxiety And Depression Scale Acta Psychiatr Scand 1983, 67:361-70
30. PA Harris, R Taylor, BL Minor, V Elliott, M Fernandez, L O'Neal, L McLeod, G Delacqua, F Delacqua, J Kirby, SN Duda, REDCap Consortium, The REDCap consortium: Building an international community of software partners, *J Biomed Inform. 2019 May 9 [doi: 10.1016/j.jbi.2019.103208]*
31. Holland AE, Hill CJ, Connon M, *et al*. Short term improvement in exercise capacity and symptoms following exercise training in interstitial lung disease. Thorax 2008; 63: 549-554.
32. Perez-Bogerd S., Wuyts W, Barbier V, Demeyer H, Van Muylem A, Janssens W, Troosters T. Short and long-term effects of pulmonary rehabilitation in interstitial lung diseases: a randomised controlled trial. Respiratory Research 2018; 19 (1): 182.
33. Bell EC, Cox NS, Goh N, et al. Oxygen therapy for interstitial lung disease: a systematic review. Eur Respir Rev 2017: 26: 160080 [https://doi.org/10.1183/16000617.0080-2016]
34. Holland AE, Hill CJ, Connon M, *et al*. Small changes in six-minute walk distance are important in diffuse parenchymal lung disease. Respir Med 2009; 103:1430-5.
35. du Bois RM, Weycker D, Albera C, et al. Six-minute walk test in idiopathic pulmonary fibrosis; test validation and minimal clinically important difference. Am J Respir Crit Care Med, 2011; 183: 1231-1237.
36. Tonelli R, Cocconcelli E, Lanini B, Romagnoli I et al. Effectiveness of pulmonary rehabilitation in patients with interstitial lung disease of different etiology; a multicentre prospective study. BMC Pulmonary Medicine 2017; 17: 1-9.
37. Nishiyama O, Kondoh Y, Kimura T, Kato K, Kataoka K, Ogawa T, Watanabe F, Arizono S, Nishimura K, Taniguchi H. Effects of pulmonary rehabilitation in patients with idiopathic pulmonary fibrosis. Respirology. 2008;13:394–9.
38. Vainshelboim B, Oliveira J, Yehoshua L, Weiss I, Fox BD, Fruchter O, Kramer MR. Exercise training-based pulmonary rehabilitation program is clinically beneficial for idiopathic pulmonary fibrosis. Respiration. 2014;88:378–88.

For peer review only - http://bmjopen.bmj.com/site/about/guidelines.xhtml

39. Ferreira A, Garvey C, Connors GL, Hilling L, Rigler J, Farrell S, Cayou C, Shariat C, Collard HR. Pulmonary rehabilitation in interstitial lung disease: benefits and predictors of response. Chest. 2009;135:442–7.
40. Jackson RM, Gomez-Marin OW, Ramos CF, Sol CM, Cohen MI, Gaunaurd IA, Cahalin LP, Cardenas DD. Exercise limitation in IPF patients: a randomized trial of pulmonary rehabilitation. Lung. 2014;192:367–76.
41. Ryerson CJ, Cayou C, Topp F, et al. Pulmonary rehabilitation improves long-term outcomes in interstitial lung disease: a prospective cohort study. Respir Med 2014;108:203–10.
42. Seymour JM, Spruit MA, Hopkinson NS, et al. The prevalence of quadriceps weakness in COPD and the relationship with disease severity. Eur Respir J 2010; 36(1): 81–88.
43. Swallow EB, Reyes D, Hopkinson NS, et al. Quadriceps strength predicts mortality in patients with moderate to severe chronic obstructive pulmonary disease. Thorax 2007; 62(2): 115–120.
44. Mendes P, Wicherson L, Helm D, et al. Skeletal muscle atrophy in advanced interstitial lung disease. Respirology 2015; 20: 953-959.
45. Mendoza L, Gogali A, Shrikrishna D, Cavada G, et al. Quadriceps strength and endurance in fibrotic idiopathic interstitial pneumonia. Respirology 2014; 19: 138-143.
46. de Troyer A, Yernault JC, Inspiratory muscle force in normal subjects and patients with interstitial lung disease. Thorax 1980; 35: 92-100.
47. Tonelli R, Cocconcelli E, Lanini B, et al. Effectiveness of pulmonary rehabilitation in patients with interstitial lung disease of different etiology; a multicentre prospective study. BMC Pulmonary Medicine 2017; 17: 130-139.
48. Dowman LM, McDonald CF, Bozinovski S, Vlahos R, Gillies R, Pouniotis D, Hill CJ, Goh NSL, Holland AE. Greater endurance capacity and improved dyspnoea with acute oxygen supplementation in idiopathic pulmonary fibrosis patients without resting hypoxaemia. Respirology 2017; 22: 957-964.
49. Johnson-Warrington V, Williams J, Bankart J, Steiner M, Morgan M, Singh S. Pulmonary rehabilitation and Interstitial lung disease: Aiding the referral decision. J Cardiopulm Rehab and Prevent 2013; 33 (3): 189-195.

For peer review only - http://bmjopen.bmj.com/site/about/guidelines.xhtml

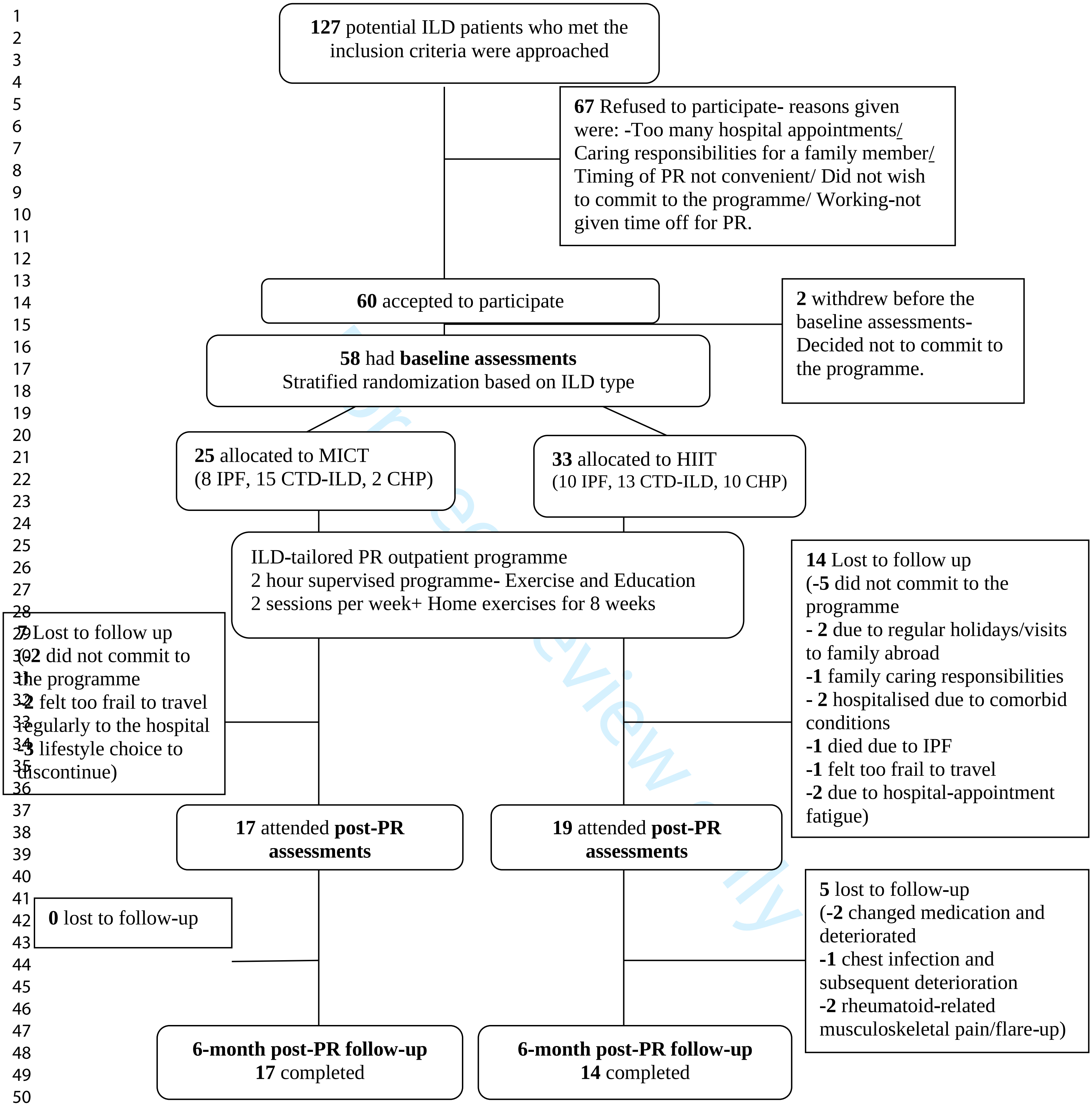

Figure 1: CONSORT diagram of participants' flow in the study. ILD-PR= pulmonary rehabilitation programme tailored for interstitial lung disease patients, MICT= moderate intensity continuous training, HIIT= high intensity interval training, IPF= idiopathic pulmonary fibrosis, CTD-ILD= connective tissue disease related interstitial lung disease, CHP= chronic hypersensitivity pneumonitis.

For peer review only - http://bmjopen.bmj.com/site/about/guidelines.xhtml

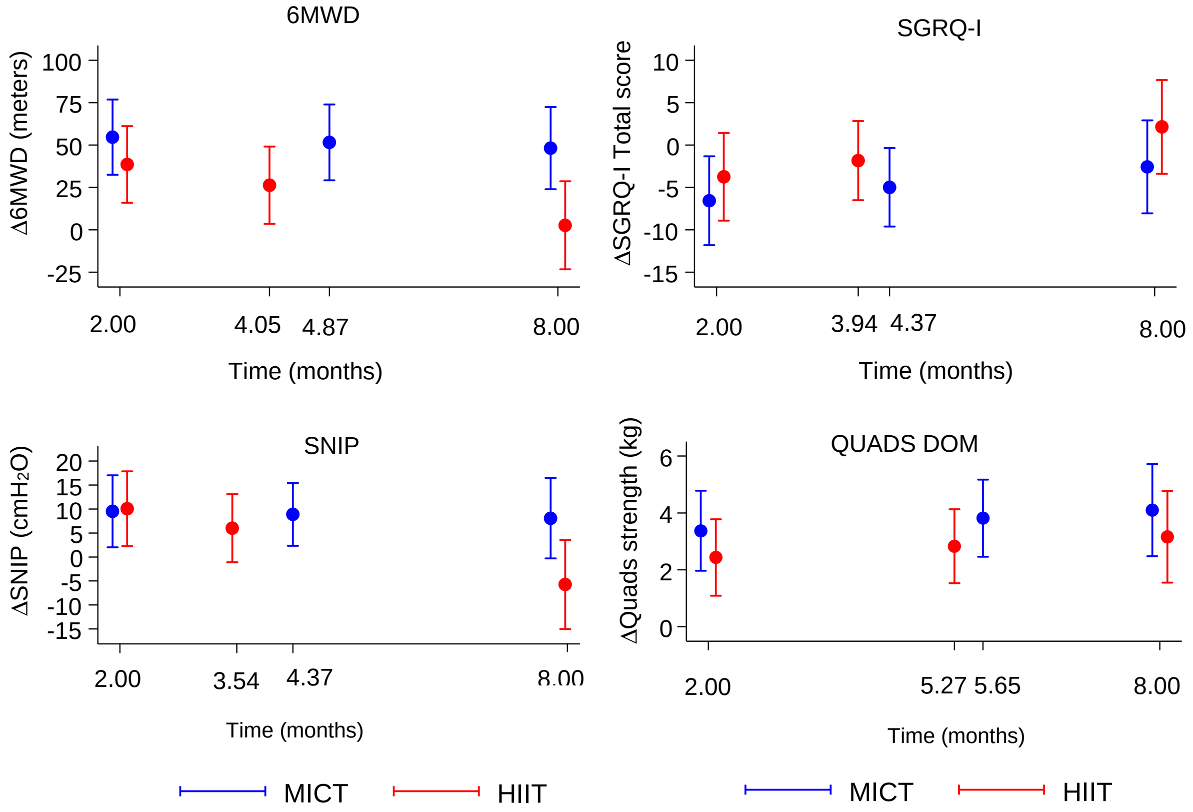

Figure 2: Change from baseline in 4 key outcomes by intervention group over time.

review only

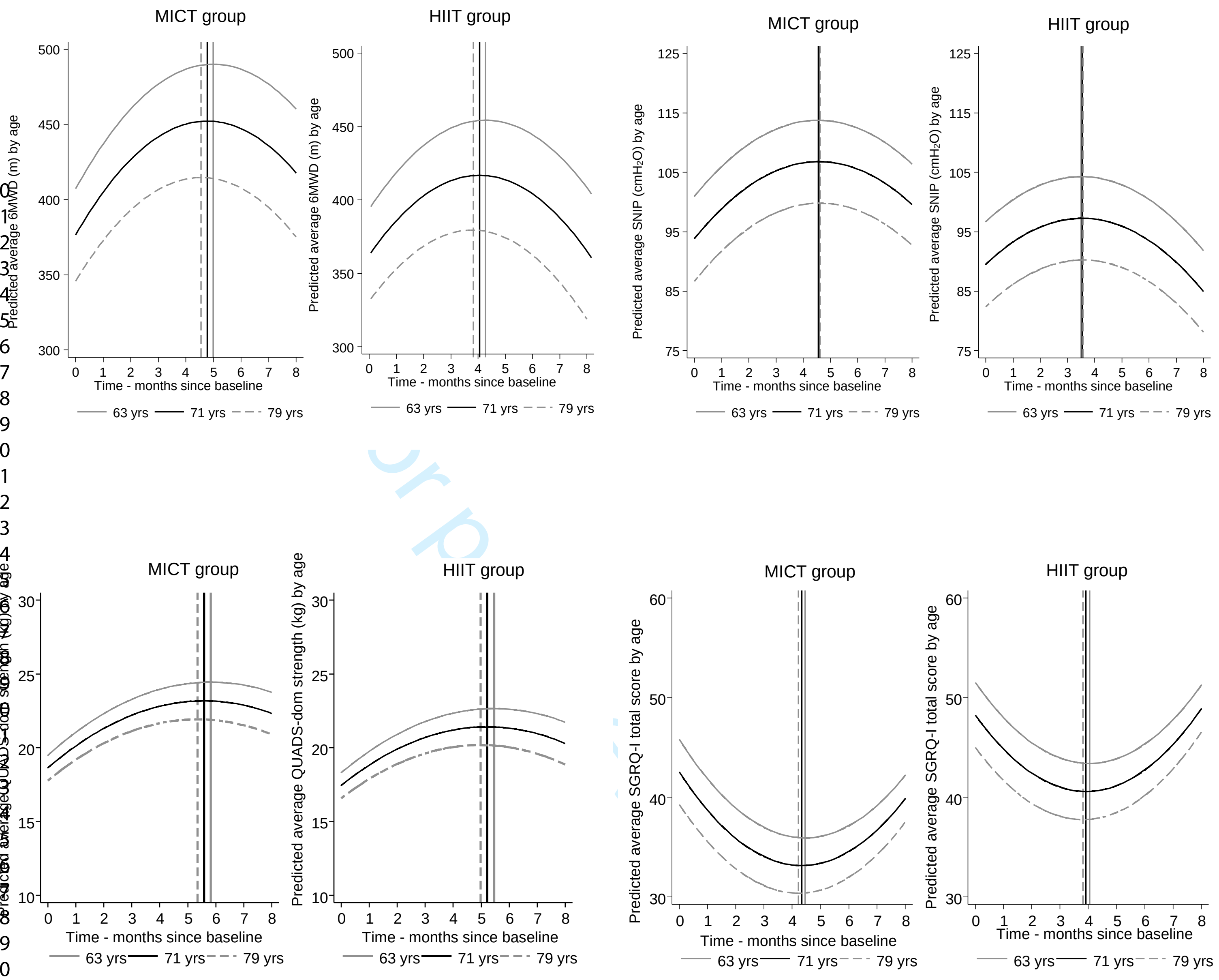

Figure 3: Age-related variations in the 4 key outcomes (6MWD, SNIP, QUADS-DOM (Quadriceps strength) and SGRQ-I total score) per training group using random effects models. The vertical line indicates the inflection point (i.e. highest or lowest values) in each age group. The ages are the mean values in each tertile

Supplementary File

Post-hoc statistical analysis on the effect of baseline DLCO on key outcomes

We have investigated DLCO in a number of different ways.

1. Baseline assessment, i.e. DLCO by intervention and clinical groups simultaneously.

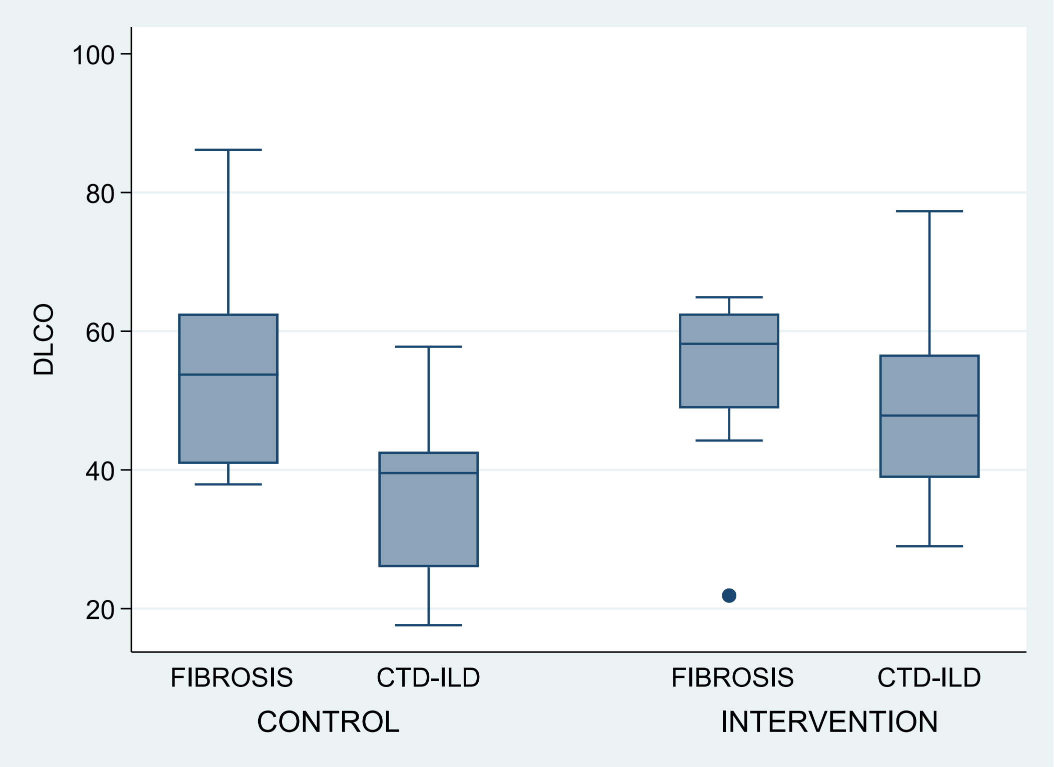

Based on these data, the evidence suggests that DLCO differs by intervention group (adjusted p value=0.003)– finding initially deems as random randomization imbalance. Nevertheless, there is not enough evidence to suggest that DCLO levels differ by clinical group, i.e. the DCLO difference between the clinical groups seems to remain similar with the intervention group (p=0.069).

2. Relationship between the 4 outcomes and DLCO at the baseline. The results are presented in Table 1 below. There was no evidence for an interaction of DLCO with either intervention or clinical group regarding these outcomes. Only coefficients corresponding to DLCO are shown for a succinct message, but they are adjusted for intervention groups and clinical groups.

Table 1: Relationship between the 4 key outcomes and baseline DLCO, adjusted for intervention and clinical groups.

	Estimate	SE	T value	p-value	95%CI low	95%CI high	No Obs	Association
MWT	.9787011	1.178773	0.83	0.410	-1.39138	3.348783	50	UNADJUSTED
SNIP	.1441871	.2431136	0.59	0.556	-.3454688	.633843	47	
QUADS DOM	-.0956757	.0499201	-1.92	**0.061**	-.1958477	.0044964	54	
SGRQI	-.0614169	.1979514	-0.31	0.758	-.4586354	.3358016	54	
MWT	8799743	1.31952	0.67	0.508	-1.774558	3.534506	50	ADJUSTED FOR
SNIP	.0873187	.2717802	0.32	0.750	-.4604183	.6350556	47	INTERVENTION
QUADS DOM	-.123836	.0548133	-2.26	**0.028**	-.2338782	-.0137938	54	GROUP

For peer review only

Supplementary File

SGRQI	.0017764	.2204537	0.01	0.994	-.4408028	.4443556	54	
MWT	1.047737	1.366581	0.77	0.447	-1.703048	3.798522	50	ADJUSTED FOR
SNIP	.1784858	.2751485	0.65	0.520	-.3764041	.7333758	47	INTERVENTION
QUADS DOM	-.1468699	.0553319	-2.65	**0.011**	-.2580074	-.0357325	54	AND CLINICAL
SGRQI	.0816139	.2234533	0.37	0.716	-.3672054	.5304331	54	GROUP
MWT	1.078732	1.302553	0.83	0.412	-1.544745	3.702209	50	ADJUSTED FOR
SNIP	.181307	.2570293	0.71	0.484	-.337399	.7000131	47	INTERVENTION,
QUADS DOM	-.1478509	.0529411	-2.79	**0.007**	-.25424	-.0414617	54	CLINICAL
SGRQI	.0764789	.2163264	0.35	0.725	-.3582453	.5112032	54	GROUP AND AGE

MWT=distance in the 6MWT, SNIP=Sniff nasal inspiratory pressure, QUADS DOM=Quadriceps strength in the dominant leg and SGRQ-I=total score in the St George's Respiratory Questionnaire for ILD

There is some evidence to suggest that, at baseline, the DLCO is negatively associated with QUADS DOM but, based on these data, none of the other important measurements seemed to have been affected.

3. However, when analysing the data longitudinally, after adjusting for time and its quadratic term, interactions between time and intervention and clinical groups and age, the effect on the QUADS DOM seem to weaken. Below, we additionally adjust the models presented in Table 1 by DLCO – after investigating its interactions with intervention/clinical groups too. Only coefficients corresponding to DLCO are shown for a succinct message, but they are adjusted for time, intervention groups and clinical groups, age and their interactions.

Table 2: Relationship between DLCO and key outcomes, adjusted for time, intervention groups and clinical groups, age and their interactions.

	Estimate	SE	T value	p-value	95%CI low	95%CI high	No Obs	Association
MWT	1.336415	.9914012	1.35	0.178	-.6066961	3.279525	113/53	ADJUSTED FOR
SNIP	.3109333	.2361878	1.32	0.188	-.1519863	.7738529	113/53	INTERVENTION,
QUADS DOM	-.0910743	.0473067	-1.93	**0.054**	-.1837937	.0016452	119/55	CLINICAL
SGRQI	.1404341	.197889	0.71	0.478	-.2474212	.5282893	120/55	GROUP AND AGE

MWT=distance in the 6MWT, SNIP=Sniff nasal inspiratory pressure, QUADS DOM=Quadriceps strength in the dominant leg and SGRQ-I=total score in the St George's Respiratory Questionnaire for ILD

This is not to say that the effect on these outcomes may not be important, we just did not capture it based on these data.

Table 1: Descriptive values at each assessment point

	Control group (MICT) Mean (SD)			Intervention group (HIIT) Mean (SD)		
	Baseline N= 25	2M (Post-PR) N=17	8M (6M post-PR) N= 17	Baseline N=33	2M (Post-PR) N=19	8M (6M post-PR) N=14
**BMI (kg/m^2)**	27.5 (3.8)	28.3 (4.1)	27.8 (3.9)	27.2 (5.1)	26.5 (4.9)	26.0 (4.7)
**Waist/Hip ratio**	0.9(0.1)	0.9 (0.1)	0.9 (0.0)	0.9 (0.1)	0.9 (0.1)	0.9 (0.1)
**%fat**	29.9 (9.3)	33.2 (9.8)	33.7 (8.7)	30.1 (8.1)	27.9 (10.7)	28.5 (9.6)
**FVC%,pred.**	85.8 (23.9)	88.0 (17.5)	88.7 (24.4)	73.8 (18.8)	68.4 (22.4)	71.3 (24.1)
**DLCO%, pred.**	53.9 (13.1)	53.6 (13.5)	56.5 (21.1)	41.7 (13.9)	44.9 (20.2)	44.8 (20.3)
**6MWD (m)**	380 (139.8)	453.9 (108.1)	437.4 (107.9)	364 (108.3)	434.3 (102.3)	421.9 (123.0)
**SNIP (cmH_2O)**	96.8 (25.9)	103.0 (21.04)	97.8 (21.4)	93.1 (24.5)	102.2 (34.1)	95.3 (29.5)
**PImax(cmH_2O)**	91.1(34.2)	104.3 (28.5)	104.9 (24.0)	91.8 (27.4)	92.8 (28.6)	95 (31.7)
**PEmax(cmH_2O)**	114.4 (40.3)	111.1 (33.3)	106.8 (34.5)	100.7 (23.5)	105.1 (24.2)	106.9 (24.7)
**Handgrip (kg)**	27.2 (11.2)	28.4 (8.4)	27.4 (9.3)	24.8 (9.4)	26.2 (11.4)	27.4 (11.5)
**Quads extension (dom) (kg)**	18.7 (6.2)	21.8 (5.0)	22.8 (6.1)	18.0 (5.0)	20.0 (5.6)	21.5 (5.4)
**Hip flexion (dom) (kg)**	15.3 (5.1)	17.9 (4.1)	18.5 (5.1)	15.9 (4.3)	16.5 (4.7)	18.1 (6.3)
**HAD-A**	6.2 (4.6)	4.8 (3.3)	5.1 (3.7)	6.4 (3.9)	6.4 (4.6)	7.2 (4.2)
**HAD-D**	5.8 (3.2)	5.0 (3.3)	5.2 (2.3)	5.2 (4.0)	5.1 (3.4)	6.6 (4.2)
**SGRQ-I Total score**	43.3 (20.3)	35.1 (15.3)	41.5 (19.3)	49.3 (21.8)	40.2 (16.7)	48.9 (21.6)

For peer review only

Table 2: Average predictions for main outcome values at the main time points during the trial, i.e. baseline, 2 months, 8 months and *changes from the baseline* at 2 months, 8 months and by the time they reach their max/min values. This set of predictions are made across intervention and ILD clinical groups, adjusting for age (hence predictions are made for patients of average age of 71 years). Estimated peak/saddle times are also displayed.

For peer review only

Outcome	Time	Control (MICT)				Intervention (HIIT)			
		Fibrosis		CTD-ILD		Fibrosis		CTD-ILD	
		Mean (95% CI) predicted value	Change from baseline	Mean (95% CI) predicted value	Change from baseline	Mean (95% CI) predicted value	Change from baseline	Mean (95% CI) predicted value	Change from baseline
6MWD	**Baseline**	415.84 (360.8 to 470.9))		393.89 (337.8 to 450.0)		367.94 (327.4 to 408.5)		358.88 (304.8 to 413.0)	
	**2 months**	464.63 (409.4 to 519.84)	45.5 (17.5 to 73.5)	442.92 (387.3 to 498.6)	55.4 (35.3 to 75.6)	401.22 (360.1 to 442.3)	28.9 (5.7 to 52.0)	410.63 (356.3 to 465.0)	38.3 (14.2 to 63.5)
	**8 months**	459.03 (398.5 to 519.6)	39.2 (11.2 to 67.2)	438.02 (381.6 to 494.5)	49.2 (28.8 to 69.6)	349.07 (304.9 to 393.3)	- 6.84 (-30.9 to 17.3)	413.93 (351.5 to 476.4)	3.1 (-23.5 to 29.7)
	**Peak value**	490.40 (432.7 to 548.1)	42.5 (15.2 to 69.8)	469.02 (412.6 to 525.5)	52.4 (33.1 to 71.8)	409.60 (366.8 to 452.4)	19.2 (-3.3 to 41.7)	440.80 (382.9 to 498.7)	20.4 (-4.2 to 45.1)
	**Peak time-months**	4.85 (4.1 to 5.6)		4.87 (4.4 to 5.4)		3.63 (3.1 to 4.2)		5.09 (4.3 to 5.9)	
SNIP	**Baseline**	99.94 (86.3 to 113.5)		90.69 (77.4 to 104.0)		96.92 (86.5 to 107.3)		77.55 (64.5 to 90.6)	
	**2 months**	106.89 (93.4 to 120.4)	14.3 (4.0 to 24.5)	100.33 (87.2 to 113.4)	10.9 (3.7 to 18.0)	102.38 (91.6 to 113.1)	8.6 (0.2 to 16.9)	85.46 (72.4 to 98.6)	5.2 (-3.9 to 14.2)
	**8 months**	97.52 (79.9 to 115.2)	5.8 (-5.3 to 16.9)	99.04 (84.9 to 113.4)	2.4 (-5.6 to 10.5)	88.54 (75.3 to 101.8)	0.1 (-9.4 to 9.7)	78.98 (62.5 to 95.5)	-3.3 (-13.2 to 6.6)
	**Peak value**	108.84 (94.3 to 123.4)	11.8 (1.8 to 21.8)	105.37 (91.6 to 119.2)	9.2 (2.4 to 16.0)	103.23 (91.7 to 114.8)	4.6 (-3.6 to 12.6)	88.35 (73.9 to 102.7)	2.2 (-6.6 to 10.9)
	**Peak time-months**	3.76 (2.1 to 5.4)		4.83 (3.8 to 5.9)		3.17 (1.9 to 4.5)		4.14 (2.8 to 5.5)	
QUADS DOM	**Baseline**	18.13 (15.4 to 20.9)		19.68 (16.9 to 22.4)		17.66 (15.6 to 19.7)		17.71 (15.1 to 20.3)	
	**2 months**	21.20 (18.4 to 24.0)	4.6 (2.6 to 6.7)	22.10 (19.4 to 24.8)	2.7 (1.2 to 4.2)	20.09 (17.9 to 22.3)	2.8 (1.2 to 4.3)	20.25 (17.6 to 22.9)	2.4 (0.4 to 4.4)
	**8 months**	23.64 (19.7 to 27.6)	5.4 (3.2 to 7.7)	22.59 (19.6 to 25.6)	3.5 (1.8 to 5.1)	20.62 (17.9 to 23.4)	3.5 (1.9 to 5.2)	21.10 (17.5 to 24.7)	3.2 (1.0 to 5.3)
	**Peak value**	23.99 (20.4 to 27.5)	5.2 (3.1 to 7.3)	23.63 (20.7 to 26.5)	3.1 (1.7 to 4.5)	21.64 (19.1 to 24.2)	3.2 (1.8 to 4.6)	21.98 (18.8 to 25.2)	2.9 (0.9 to 4.8)

For peer review only - http://bmjopen.bmj.com/site/about/guidelines.xhtml

	**Peak time-months**	6.44 (4.2 to 8.7)		5.29 (4.0 to 6.6)		5.31 (4.0 to 6.6)		5.50 (3.8 to 7.2)	
**SGRQ-I**	**Baseline**	44.01 (33.2 to 54.9)		38.42 (27.6 to 49.2)		55.00 (47.0 to 63.0)		37.75 (27.6 to 47.9)	
	**2 months**	37.36 (26.4 to 48.3)	- 8.4 (-16.1 to -0.7)	32.11 (21.5 to 42.7)	-6.6 (-12.0 to -1.2)	49.72 (41.5 to 58.0)	-3.5 (-9.6 to 2.5)	31.38 (21.2 to 41.6)	- 6.5 (-13.9 to 0.9)
	**8 months**	40.39 (27.4 to 53.4)	3.1 (- 10.9 to 4.7)	36.18 (25.2 to 47.2)	-1.3 (-6.7 to 4.2)	56.87 (47.6 to 66.1)	1.8 (-4.3 to 7.8)	35.28 (23.2 to 47.4)	-1.2 (-8.7 to 6.35)
	**Min value**	34.43 (22.6 to 46.3)	-6.2 (-13.7 to 1.3)	29.59 (18.6 to 40.6)	- 4.6 (-9.7 to 0.5)	48.25 (39.3 to 57.2)	- 1.9 (-7.8 to 3.8)	28.80 (17.8 to 39.8)	-4.4 (-11.7 to 2.8)
	**Min time-months**	4.47 (3.2 to 5.8)		4.29 (3.5 to 5.1)		3.76 (2.9 to 4.7)		4.32 (3.2 to 5.5)	

Data are mean (95% CI)

For peer review only

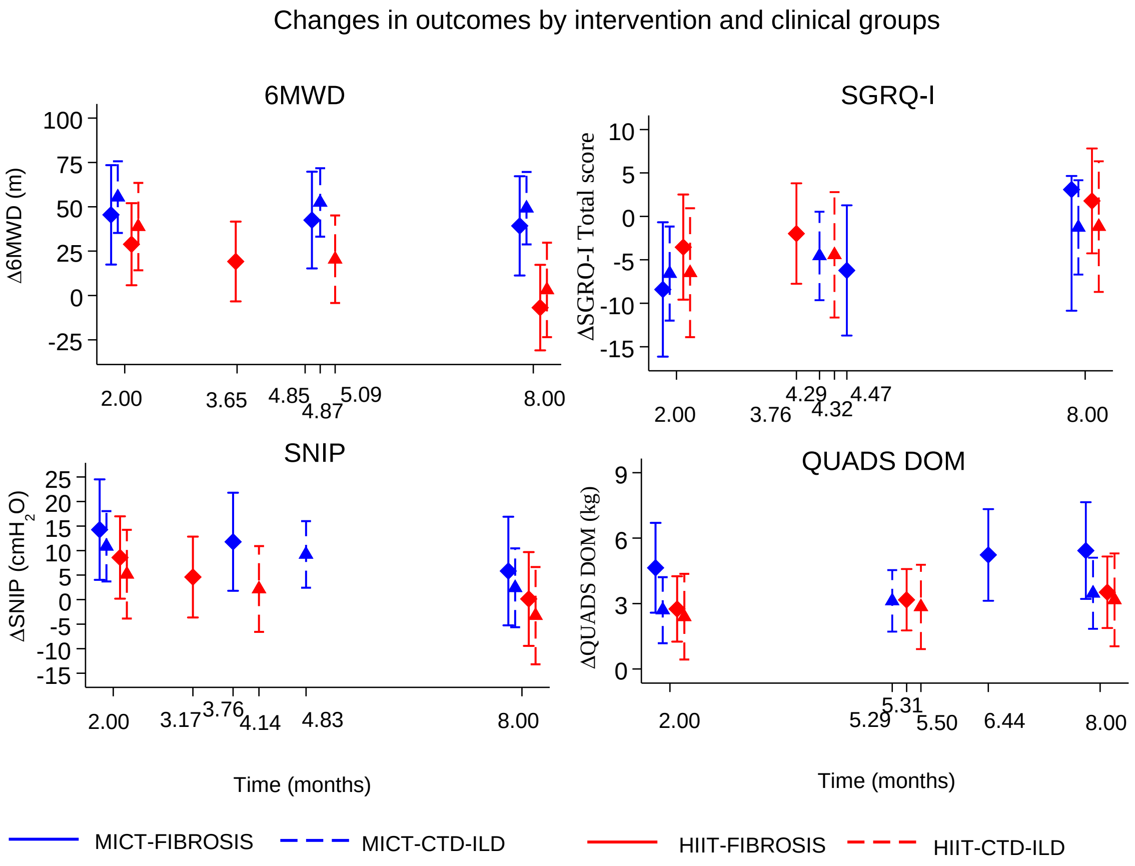

Figure 1: Changes in key outcomes by intervention and ILD clinical groups

For peer review only

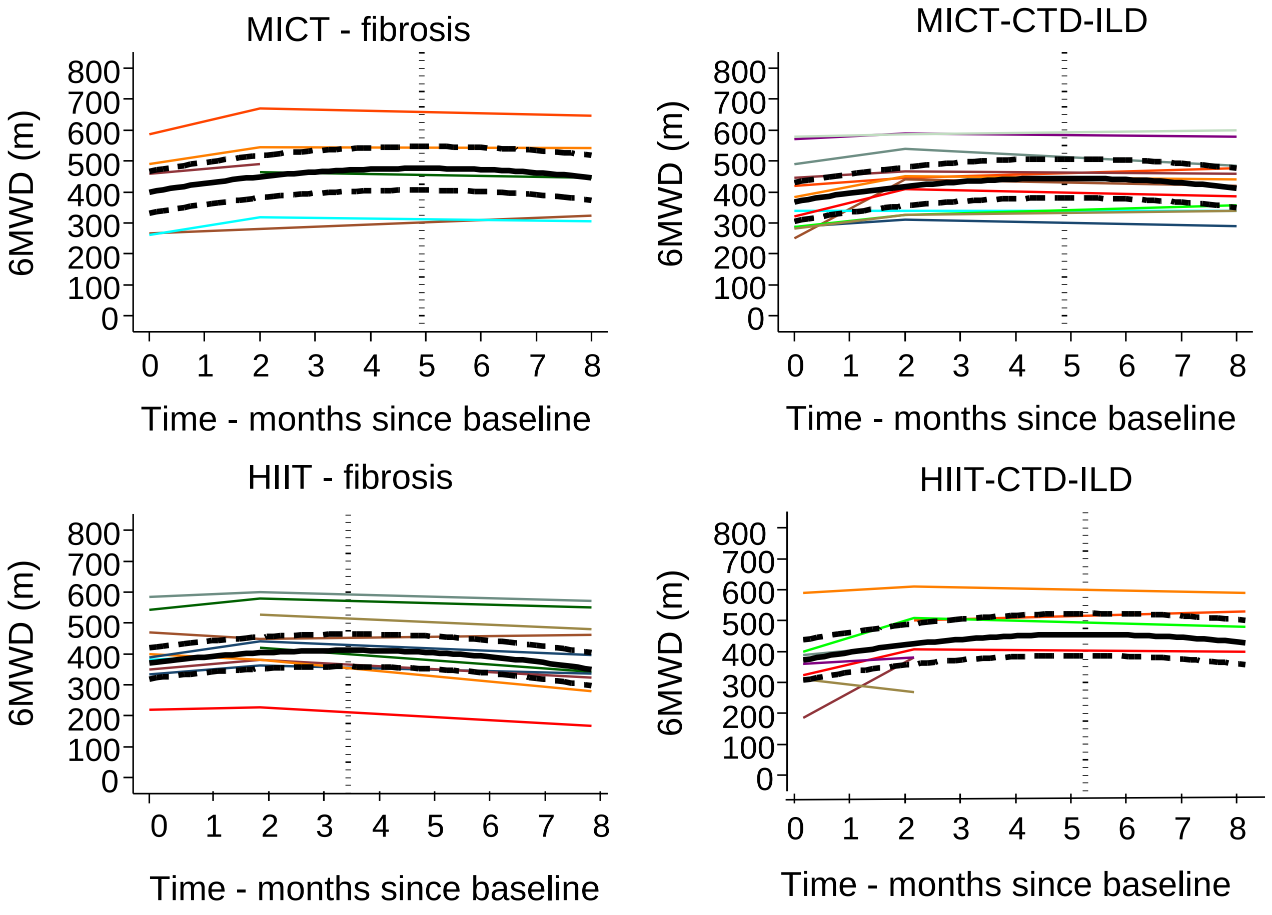

Figure 2: Random effects models by intervention (HIIT vs MICT) and ILD clinical subgroup (Fibrosis vs CTD-ILD)

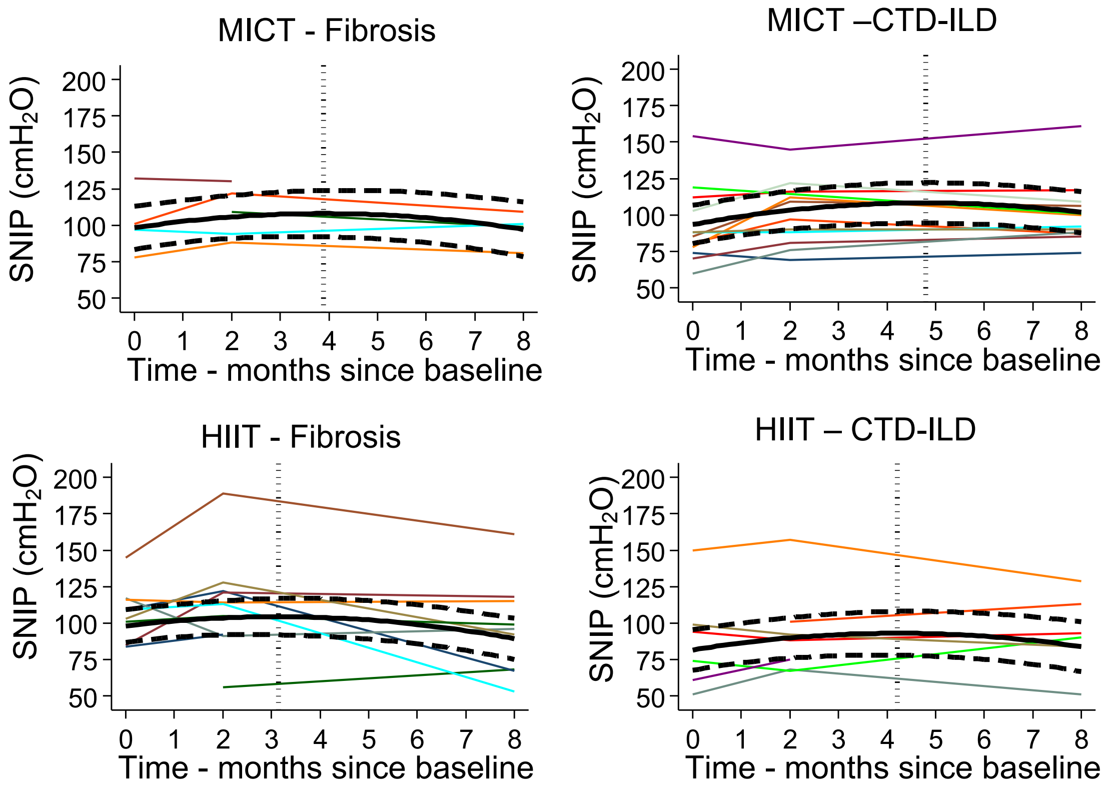

Figure 3: Random effects models by intervention (HIIT vs MICT) and ILD clinical subgroup (Fibrosis vs CTD-ILD)

For peer review only - http://bmjopen.bmj.com/site/about/guidelines.xhtml

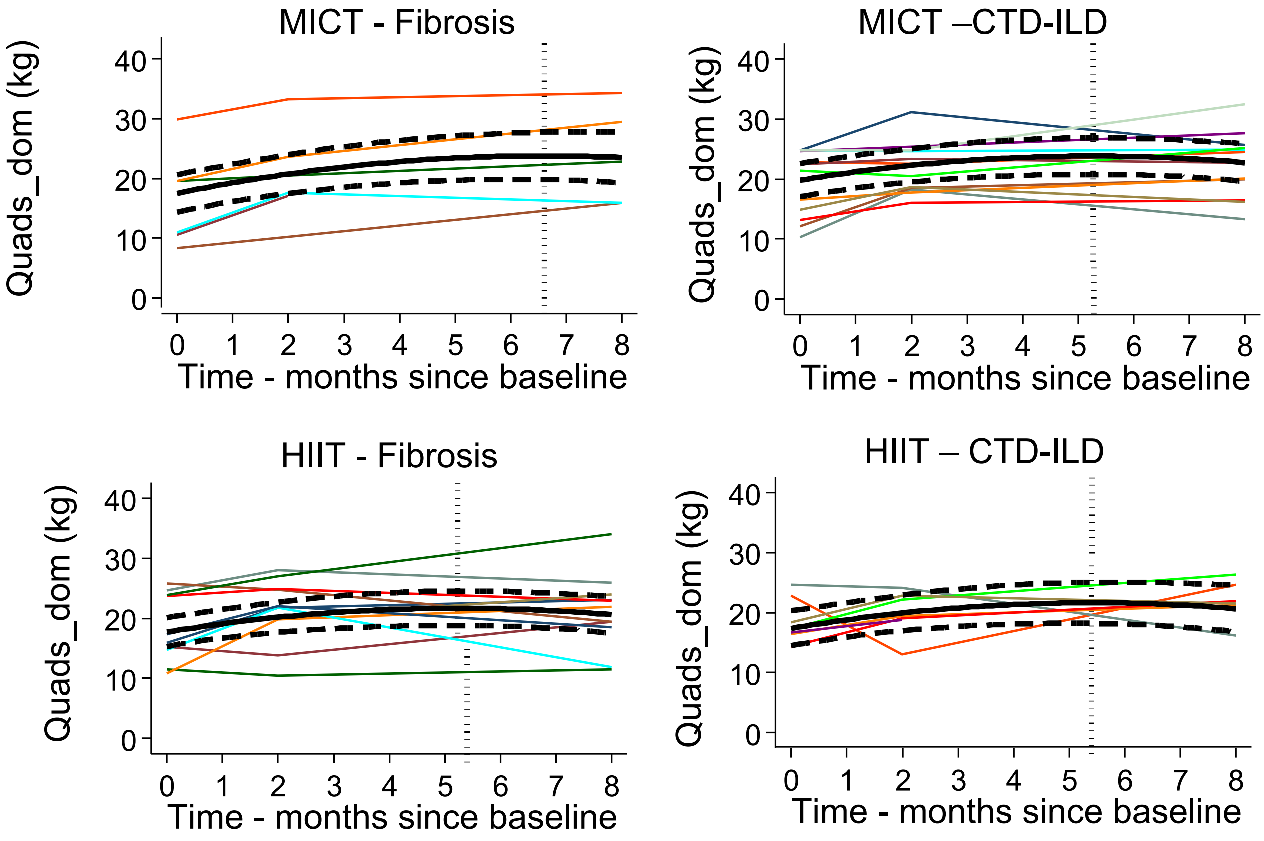

Figure 4: Random effects models of quadriceps strength (dominant side) by MICT vs HIIT and ILD clinical subgroups.

Reporting checklist for randomised trial.

Based on the CONSORT guidelines.

Instructions to authors

Complete this checklist by entering the page numbers from your manuscript where readers will find each of the items listed below.

Your article may not currently address all the items on the checklist. Please modify your text to include the missing information. If you are certain that an item does not apply, please write "n/a" and provide a short explanation.

Upload your completed checklist as an extra file when you submit to a journal.

In your methods section, say that you used the CONSORTreporting guidelines, and cite them as:

Schulz KF, Altman DG, Moher D, for the CONSORT Group. CONSORT 2010 Statement: updated guidelines for reporting parallel group randomised trials

		Reporting Item	Page Number
**Title and Abstract**			
Title	#1a	Identification as a randomized trial in the title.	1
Abstract	#1b	Structured summary of trial design, methods, results, and conclusions	2
**Introduction**			
Background and objectives	#2a	Scientific background and explanation of rationale	4

For peer review only

Background and objectives	#2b	Specific objectives or hypothesis	5
**Methods**			
Trial design	#3a	Description of trial design (such as parallel, factorial) including allocation ratio.	5 and Figure 1
Trial design	#3b	Important changes to methods after trial commencement (such as eligibility criteria), with reasons	n/a
Participants	#4a	Eligibility criteria for participants	5
Participants	#4b	Settings and locations where the data were collected	5
Interventions	#5	The experimental and control interventions for each group with sufficient details to allow replication, including how and when they were actually administered	6-7
Outcomes	#6a	Completely defined prespecified primary and secondary outcome measures, including how and when they were assessed	8-9
Sample size	#7a	How sample size was determined.	9
Sample size	#7b	When applicable, explanation of any interim analyses and stopping guidelines	n/a

For peer review only

Randomization - Sequence generation	#8a	Method used to generate the random allocation sequence.	6
Randomization - Sequence generation	#8b	Type of randomization; details of any restriction (such as blocking and block size)	6
Randomization - Allocation concealment mechanism	#9	Mechanism used to implement the random allocation sequence (such as sequentially numbered containers), describing any steps taken to conceal the sequence until interventions were assigned	6
Randomization - Implementation	#10	Who generated the allocation sequence, who enrolled participants, and who assigned participants to interventions	6
Blinding	#11a	If done, who was blinded after assignment to interventions (for example, participants, care providers, those assessing outcomes) and how.	6
Blinding	#11b	If relevant, description of the similarity of interventions	6 and table 1
Statistical methods	#12a	Statistical methods used to compare groups for primary and secondary outcomes	9
Statistical methods	#12b	Methods for additional analyses, such as subgroup analyses and adjusted analyses	9

For peer review only

Outcomes	#6b	Any changes to trial outcomes after the trial commenced, with reasons	n/a
**Results**			
Participant flow diagram (strongly recommended)	#13a	For each group, the numbers of participants who were randomly assigned, received intended treatment, and were analysed for the primary outcome	10-Figure 1
Participant flow	#13b	For each group, losses and exclusions after randomization, together with reason	Fig 1
Recruitment	#14a	Dates defining the periods of recruitment and follow-up	Fig 1
Recruitment	#14b	Why the trial ended or was stopped	n/a
Baseline data	#15	A table showing baseline demographic and clinical characteristics for each group	Table 1
Numbers analysed	#16	r	10 -Fig 1 and Table 1
Outcomes and estimation	#17a	For each primary and secondary outcome, results for each group, and the estimated effect size and its precision (such as 95% confidence interval)	10-11

For peer review only

Outcomes and estimation	#17b	For binary outcomes, presentation of both absolute and relative effect sizes is recommended	10-11
Ancillary analyses	#18	Results of any other analyses performed, including subgroup analyses and adjusted analyses, distinguishing pre-specified from exploratory	Supplementary files Tables 3-4 and Figures 4-5-6
Harms	#19	All important harms or unintended effects in each group (For specific guidance see CONSORT for harms)	Fig.1
**Discussion**			
Limitations	#20	Trial limitations, addressing sources of potential bias, imprecision, and, if relevant, multiplicity of analyses	15
Interpretation	#22	Interpretation consistent with results, balancing benefits and harms, and considering other relevant evidence	12-16
Registration	#23	Registration number and name of trial registry	1
Generalisability	#21	Generalisability (external validity, applicability) of the trial findings	2, 15-16
**Other information**			

For peer review only

Interpretation	#22	Interpretation consistent with results, balancing benefits and harms, and considering other relevant evidence	12-16
Registration	#23	Registration number and name of trial registry	1
Protocol	#24	Where the full trial protocol can be accessed, if available	5 and Protocol file
Funding	#25	Sources of funding and other support (such as supply of drugs), role of funders	21

Notes:

- 13a: 10-Figure 1
- 16: 10 -Fig 1 and Table 1
- 18: Supplement Tables 3-4, Fig 4-5-6 The CONSORT checklist is distributed under the terms of the Creative Commons Attribution License CC-BY. This checklist was completed on 29. March 2022 using https://www.goodreports.org/, a tool made by the EQUATOR Network in collaboration with Penelope.ai

For peer review only

BMJ Open

Comparison of high intensity interval training versus moderate intensity continuous training in pulmonary rehabilitation for interstitial lung disease; a randomised controlled pilot feasibility trial.

Journal:	*BMJ Open*
Manuscript ID	bmjopen-2022-066609.R2
Article Type:	Original research
Date Submitted by the Author:	14-Jul-2023
Complete List of Authors:	Nikoletou, Dimitra; St George's University of London, Centre for Allied Health, Institute of Medical and Biomedical Education; Kingston University, Faculty of Health, Science, Social Care and Education<br>Chis Ster, Irina; St George's University of London, Infection and Immunity Research Institute<br>Lech, Carmen; St George's University of London, Infection and Immunity Research Institute<br>MacNaughton, Iain; St George's University of London, Infection and Immunity Research institute<br>Chua, Felix; Royal Brompton and Harefield NHS Foundation Trust, Interstitial Lung Disease Unit; Imperial College London, National Heart and Lung Institute<br>Aul, Raminder; St George's University Hospitals NHS Foundation Trust, Respiratory Medicine<br>Jones, Paul; St George's University of London, Infection and Immunity Research institute
**Primary Subject Heading**:	Respiratory medicine
Secondary Subject Heading:	Rehabilitation medicine
Keywords:	Interstitial lung disease < THORACIC MEDICINE, REHABILITATION MEDICINE, PREVENTIVE MEDICINE

SCHOLARONE™
Manuscripts

I, the Submitting Author has the right to grant and does grant on behalf of all authors of the Work (as defined in the below author licence), an exclusive licence and/or a non-exclusive licence for contributions from authors who are: i) UK Crown employees; ii) where BMJ has agreed a CC-BY licence shall apply, and/or iii) in accordance with the terms applicable for US Federal Government officers or employees acting as part of their official duties; on a worldwide, perpetual, irrevocable, royalty-free basis to BMJ Publishing Group Ltd ("BMJ") its licensees and where the relevant Journal is co-owned by BMJ to the co-owners of the Journal, to publish the Work in this journal and any other BMJ products and to exploit all rights, as set out in our licence.

The Submitting Author accepts and understands that any supply made under these terms is made by BMJ to the Submitting Author unless you are acting as an employee on behalf of your employer or a postgraduate student of an affiliated institution which is paying any applicable article publishing charge ("APC") for Open Access articles. Where the Submitting Author wishes to make the Work available on an Open Access basis (and intends to pay the relevant APC), the terms of reuse of such Open Access shall be governed by a Creative Commons licence – details of these licences and which Creative Commons licence will apply to this Work are set out in our licence referred to above.

Other than as permitted in any relevant BMJ Author's Self Archiving Policies, I confirm this Work has not been accepted for publication elsewhere, is not being considered for publication elsewhere and does not duplicate material already published. I confirm all authors consent to publication of this Work and authorise the granting of this licence.

For peer review only

Title: Comparison of high intensity interval training versus moderate intensity continuous training in pulmonary rehabilitation for interstitial lung disease; a randomised controlled pilot feasibility trial.

Authors:

Dr Dimitra Nikoletou[1, 2,] PhD dinikole@sgul.ac.uk

Dr Irina Chis-Ster[3], PhD ichisste@sgul.ac.uk

Ms Carmen Y. Lech[3], BSc carmenyanina.lech@gmail.com

Mr Iain S. MacNaughton[3], BSc iainmacn62@gmail.com

Dr Felix Chua MD[4], PhD F.Chua@rbht.nhs.uk

Dr Raminder Aul MD[5] raminder.aul@stgeorges.nhs.uk

Professor Paul Jones[3] MD, PhD pjones@sgul.ac.uk

Authors' affiliations:

1. Centre for Allied Health, Institute of Medical and Biomedical Education, St George's University of London, Cranmer Terrace, London, SW17 ORE, UK.
2. Faculty of Health, Science, Social care and Education, Kingston University, London, UK.
3. Infection and Immunity Research Institute, St George's University of London, Cranmer Terrace, London, SW17 ORE, UK
4. Department of Respiratory Medicine, Royal Brompton & Harefield NHS Foundation Trust, London, UK.
5. Department of Respiratory Medicine, St George's Hospital NHS Foundation Trust, London SW17 ORE, UK

Corresponding author:
Dr Dimitra Nikoletou, Centre for Allied Health, St George's University of London, Cranmer Terrace, London, SW17 ORE and (honorary) Faculty of Health, Science, Social care and Education, Kingston University, London, UK.
Tel: 0208 725 2277 Email: dinikole@sgul.ac.uk

Key words: Interstitial lung disease, ILD, Pulmonary rehabilitation, Interstitial pulmonary fibrosis, exercise, high intensity interval training, HIIT.

Running head: Feasibility of HIIT versus MICT in ILD PR programmes

Trial registration: This trial was registered in ISRCTN, with the ID number ISRCTN55846300.

Word count: 3949

For peer review only

Abstract

Objectives: This study aimed to investigate the feasibility and efficacy of high intensity interval training (HIIT) compared to moderate intensity continuous training (MICT) in pulmonary rehabilitation (PR) for people with interstitial lung disease (ILD).

Design: Single-centre, randomised controlled feasibility, pilot trial.

Setting: Patients were recruited from the chest clinic of a tertiary ILD centre and attended circuit-based PR in the hospital's gym, followed by a personalised 6-month community programme.

Participants: 58 patients, stratified per ILD type, were randomised into two groups: 33 to HIIT (18M: 15F) [mean age (SD) 70.2 (11.4) years] and 25 to the MICT exercise mode (14M: 11F), [mean age (SD) 69.8 (10.8) years].

Interventions: 8-week, twice weekly, circuit-based PR programme of exercise and education, followed by a personalised 6-month community exercise programme.

Outcome measures: Feasibility outcomes included staff-to-patient ratio and dropout rates per group. Primary outcome was the 6-minute walk distance (6MWD). Secondary outcomes included the sniff nasal pressure, mouth inspiratory and expiratory pressures, handgrip and quadriceps strength and health status. Random-effects models were used to evaluate average variation in outcomes through time across the two groups.

Results: The 6MWD peaked earlier with HIIT compared to MICT (at 4 months versus 5 months) but values were lower at peak (mean (95%CI) 26.3m (3.5, 49.1) versus 51.6m (29.2, 73.9) and declined faster at 6-months post PR. Secondary outcomes showed similar faster but smaller improvements with HIIT over MICT and more consistent maintenance 6-months post-PR with MICT than HIIT.

Conclusions: HIIT is feasible in circuit-based ILD PR programmes and provides quick improvements but requires closer supervision of training and resources than MICT and benefits may be less well sustained. This would make it a less attractive option for clinical PR programmes. A definitive, multi-centre randomised controlled trial is required to address the role of HIIT in ILD.

For peer review only - http://bmjopen.bmj.com/site/about/guidelines.xhtml

Strengths and Limitations of this study

- This randomised controlled feasibility, pilot study, showed that HIIT is feasible in circuit-based PR programmes.
- Our results identify the point at which exercise reinforcement may be needed after completion of a HIIT programme.
- Although it was possible to recruit enough ILD patients from a single site, not enough IPF patients could be recruited to evaluate the effect of HIIT on this particular group.
- We did not use supplementary oxygen during PR for patients other than those already on long-term oxygen therapy.
- This study adds valuable information for the design of larger, definitive studies, on the peak and subsequent deterioration of outcomes over time depending on exercise mode.

For peer review only

Background

Interstitial lung disease (ILD) is a broad term used to describe a diverse group of pulmonary conditions that cause fibrotic changes in the lung interstitium with subsequent reduction in lung compliance and oxygen diffusion capacity [1, 2]. ILD is categorised into its different types according to clinical, radiological and pathophysiological changes and multidimensional indexes have been created to predict mortality risk [3, 4]. The clinical presentation includes breathlessness on exertion, dry and persistent cough, fatigue, progressive

For peer review only - http://bmjopen.bmj.com/site/about/guidelines.xhtml

exercise limitation and respiratory failure at the very severe stages. [5] Pharmacological treatment for patients with progressive fibrotic ILD is limited to only a few medications aiming to slow down further development of fibrosis [6] and lung transplantation, the only life-extending option, carries risks and is not available to all patients [7, 8].

Pulmonary rehabilitation (PR) is a non-pharmacological strategy that improves physical function and quality of life [5, 9] but in ILD patients the optimal training programme, especially for longer term benefits, remains unclear. [5, 10] Recent evidence suggests that patients with some types of ILD, such as asbestosis and idiopathic pulmonary fibrosis (IPF), may receive greater benefit from PR than others and effects may be more lasting in patients with milder disease [11]. The traditional exercise mode in PR is moderate intensity continuous training (MICT) which involves continuous exercise for 30-60 minutes at intensities ranging from 60-80% maximum heart rate [12]. However, other training modalities may offer greater benefits.

High intensity interval training (HIIT) is a modality characterised by intervals of high intensity exercise followed by intervals of low intensity or rest [13]. There is growing evidence that HIIT improves a broad range of cardiovascular risk factors, including insulin sensitivity and VO_2peak [14], and its safety has been evaluated in patients with cardiovascular and metabolic disease [14-17]. Respiratory patients, who cannot maintain exercise due to breathlessness, have found HIIT acceptable [18- 20] and although its overall superiority over MICT has been questioned [21], it has been used safely in ILD patients preparing for lung transplantation [22]. However, HIIT has yet to be evaluated in the context of an ILD-tailored PR programme.

The aim of this pilot study was therefore to evaluate: a) the feasibility of using HIIT in an ILD-PR programme, b) determine the short and medium-term effects of HIIT on exercise capacity, respiratory and peripheral muscle strength, breathlessness and health status and c) explore responses in different types of ILD.

Some of the results of this study have been reported previously abstract form [23].

Methods

For peer review only - http://bmjopen.bmj.com/site/about/guidelines.xhtml

Study design

Single-blind, randomised-controlled feasibility, pilot trial.

Study population

Symptomatic patients over 18 years old, of all types and severity, apart from sarcoidosis, and with a respiratory-physician diagnosis of ILD from a tertiary referral hospital, were invited to participate. This included patients with idiopathic pulmonary fibrosis (diagnostic criteria consistent with the International Consensus statement [1]), connective tissue disease related ILD, non-specific interstitial pneumonia, usual interstitial pneumonia, and chronic hypersensitivity pneumonitis. Patients were eligible to participate if they were ambulant and had symptoms of dyspnoea on exertion (grades 1 to 3 in the mMRC scale) [24]. Patients on long-term oxygen therapy (LTOT) were also included. Exclusion criteria were a history of syncope on exertion, patients having an acute exacerbation, patients with sarcoidosis and severe co-morbidities (such as musculoskeletal, neurological or cardiovascular problems) that would interfere with their ability to exercise.

Prior to being included in this study, all participants had full lung function tests, performed by specialised technicians in a lung function laboratory and CT scans were part of their clinical diagnostic process and management. Participants were monitored throughout the study by respiratory physicians. The usual medical care was not affected by participation in this study.

All participants provided written informed consent. The trial protocol was pre-registered with the ISRCTN registry (ISRCTN55846300) and approved by the South East Coast-Surrey Ethics committee (14/LO/0149).

Patient and Public involvement

For peer review only - http://bmjopen.bmj.com/site/about/guidelines.xhtml

People with ILD were involved in the original design of this research and determined acceptability of the proposed interventions and likelihood of adherence to this programme. During the study, all participants were involved in the conduct of the research by giving regular feedback in the education-discussion part of the PR programme and via interviews. On completion of this study, some participants formed a local ILD-Support Group and viewed the final results for dissemination.

Randomisation and blinding

Following baseline assessments, participants were randomised into 2 groups: exercise using moderate intensity continuous training (MICT group) or exercise using high-intensity interval training (HIIT group). Participants were stratified by ILD type to ensure a balanced distribution of types between groups. They were placed into 3 sub-groups: a) Idiopathic group (e.g. IPF); b) Autoimmune group (CTD-ILD) and c) Chronic Hypersensitivity pneumonitis group (CHP/EAA). The investigator performing all assessments and the statistician were blinded throughout the study.

Intervention

The ILD-PR programme

This was a circuit-based 8-week, twice weekly, outpatient programme, including only people with ILD and consisting of an hour of exercise and an hour of education. Exercise was set, supervised and progressed by two experienced physiotherapists, according to a standardised protocol for each group allocation. Supplementary oxygen was not used during the sessions. Participants already on LTOT continued to exercise with oxygen as recommended but were not given additional oxygen. Aerobic exercises included treadmill walking, brisk walking between 2 cones, cycle ergometer, trampette and step ups. Strength training exercises, upper and lower limb, were conducted using ‘Thera-bands’ and functional movements such as sit-to-stand and ball raises.

For peer review only - http://bmjopen.bmj.com/site/about/guidelines.xhtml

For peer review only

A minimum of 30 minutes were dedicated to aerobic training. Aerobic training intensity at the MICT group was set at 60% maximum heart rate (HRmax) and progressed each week. In the HIIT group, intensity was set at 80% HRmax and patients were instructed to exercise in dynamic intervals followed by low intensity exercise or rest. The aim in this group was to increase the amount of time on the 'high intensity' phase each week.

In the first week, the average time on HIIT (at 80% HRmax) for each aerobic exercise was 2.5 min, therefore HIIT was 12.5 minutes of the total 30-minute session, and this time increased each week. Progression of training was determined by reducing time in the low intensity phase of HIIT and/or increasing time in the target HR . Each participant's individualised programme was recorded on exercise logs during the sessions.

Heart rate and oxygen saturation were monitored using pulse oximeters and perceived exertion and breathlessness were recorded at set intervals using the modified 10-point Borg scale and the Rate of Perceived Exertion scale (RPE) respectively [25, 26].

The education session, on the 2nd hour of the programme was common for all participants and included presentations and discussion on topics important to ILD patients, such as: Topics about breathing and breathlessness (mechanics of breathing, helpful/unhelpful breathing patterns, the Active Cycle of Breathing technique, useful positions for recovering, pacing), topics about ILD (what happens with ILD, cough, associated conditions-rheumatoid Arthritis, Gastro-Oesophageal Reflux disease, Sjögren's syndrome, Bird Fancier's Lung, Idiopathic Pulmonary Fibrosis) and topics about managing the condition (Why healthier lifestyle is important, keeping good posture, barriers to activity/exercise, dietary advice, smoking, incontinence, relaxation information, mood, anxiety and breathlessness, self-management and goal setting).

For peer review only - http://bmjopen.bmj.com/site/about/guidelines.xhtml

On the first day of the programme all participants were given an ‘ILD-Pulmonary rehabilitation booklet’ designed by our team, which contained information and pictures of all exercises, an exercise diary and summary of the education topics (see Supplementary file 1). All participants were encouraged to continue exercising at home on the days they did not attend the programme.

The 6-month programme

Following completion of the PR programme, participants were given a personalised exercise programme, starting at the level achieved at the end of PR, and were asked to continue to exercise 3 times per week as well as do daily walking. The plan was recorded in their personal booklet. The 6-month programme included outdoor walking in HIIT or MICT mode, as well as stretching and strength training exercises. Some participants joined their local gym or organised exercise classes to ensure participation in group exercise at least once per week and followed a home programme the remaining time. Others opted for home-based exercise only. Regardless of the choice, during the 6-month period, participants were self-monitoring and had regular phone calls (once per month) and at least one home visit by the research physiotherapists on the 3rd month. The physiotherapists guided participants on filling in the exercise diaries and progressing training depending on mode of exercise. The ILD-PR booklet was used as a resource for the home exercises and as a reminder of the educational topics.

Outcomes

All assessments were performed by the same investigator (DN), using identical instructions and methods, at three time points; at baseline, at 8-weeks (post-PR) and at 6 months (post-community programme). The investigator was blinded throughout the study.

For peer review only - http://bmjopen.bmj.com/site/about/guidelines.xhtml

Feasibility outcomes included the proportion of eligible patients who consented to the study, attrition in each group, staff-to-patient ratio and supervision requirements, reasons for discontinuing the programme and adverse events.

The primary efficacy outcome was functional exercise capacity and was measured using the 6-minute walk test distance (6MWD) as recommended by the ATS guidelines [27] using standardised instructions.

Secondary outcomes were: a) Respiratory muscle function- Sniff nasal pressure (SNIP) and the maximal inspiratory and expiratory pressures (PImax and PEmax) were recorded using a handheld portable respiratory muscle testing device (MicroRPM, CareFusion, Basingstoke, UK). A minimum of 10 attempts were made for each test, on at least one session. PImax was recorded from functional residual capacity (FRC) and PEmax from total lung capacity (TLC).

b) Peripheral muscle function- Handgrip strength, assessed in the dominant hand, with the elbow at 90 degrees angle, using a hydraulic hand dynamometer (JAMAR Hydraulic Hand Dynamometer, Homecraft Rolyan Ltd, Nottinghamshire, UK). Quadriceps strength (QUADS-DOM) and hip flexion (HIP-DOM) were assessed in sitting position, with the knee at 90 degrees angle and back upright and supported using a hand-held dynamometer (Lafayette Manual Muscle Tester Model 01163, Lafayette Instruments, Lafayette, IN, USA).

c) Health status, using the St George's Respiratory Questionnaire for IPF (SGRQ-I) [28] and the Hospital Anxiety and Depression scale (HAD) [29].

Spirometry data and anthropometric measurements (height, weight, %fat, waist, hip and neck circumference) were also collected.

Data were collected and managed using REDCap electronic data capture tools hosted at the NHS Trust [30].

Statistical analysis

For peer review only - http://bmjopen.bmj.com/site/about/guidelines.xhtml

This was a pilot study to test feasibility of the HIIT exercise mode and inform the design and power of a larger, next phase, randomised controlled trial. Intention-to-treat analyses are presented- that is, all participants who were randomised were considered in the analysis, not only those who completed the programme- together with estimates' uncertainties as 95% CIs. This is not a hypothesis testing setting, hence the numbers followed instructions from the literature [31]. An indicative sample size calculation was based on previous research aiming to detect between-group changes of 38± 43m in the 6MWD following rehabilitation [32-34], with an 80% power and a probability of type 1 error of 0.05.

Exploratory analyses used random effects (mixed) models to estimate average values in outcomes across the follow-up time for the four continuous longitudinal outcomes of interest, i.e., 6MWD, SNIP, SGRQ-I and Quads strength which were relatively normally distributed on each occasion. Average estimates of these measurements were derived following fitting a series of a nonlinear, quadratic mixed models over time allowing for a 3-way interaction between time, intervention (HIIT vs MICT) and clinical group (ILD type) as well as between age, intervention and clinical group. For each of the 4 key exploratory outcomes of interest , a series of nested models of increasing complexity were developed and tested for their statistical improvement against the next less complex model. The complete data analysis (using missing data jargon) using mixed modelling on the 4 continuous longitudinal outcomes accounted for all participants and their complete observations (intention to treat analyses) and operated under 'missing at random' assumption for the attrition at the follow-up- assumption which is not testable from the data at hand

[35]. Although the missing data are less important in such pilot/feasibility settings [31], we did investigate its patterns- particularly to assess potential differences between groups defined by intervention and /or clinical characteristics Longitudinal binary outcomes were defined as 1 for a missing observation and 0 otherwise- at the baseline and at the follow-up and for each outcome- and have been explored using longitudinal models with random effects. The results of these exploratory analyses are presented in Supplementary file 2.

For peer review only - http://bmjopen.bmj.com/site/about/guidelines.xhtml

The average variation of outcomes through time, within and between-groups, are presented as means (95% CI) and descriptive statistics as mean (SD). All analyses were conducted on STATA software (version 11).

Results

Patient flow

60 participants consented to the study but two participants withdrew prior to commencement of the PR programme. A total of 58 started the PR programme. Figure 1 is the CONSORT diagram showing participant flow.

Figure 1 around here

Baseline characteristics are shown in Table 1 . There were no differences between groups at baseline, except for DLco which was significantly lower in the HIIT group.

Table 1: Baseline characteristics

Variable	Total Group (n= 58)	Control group (MICT) (n=25)	Intervention group (HIIT) (n=33)	Between group P value
**Age (years)**	70.0 (11.05)	69.8 (10.8)	70.5 (10.9)	0.89
**Gender (male), (number (%))**	32 (55.2)	14 (56)	18 (55)	0.91

For peer review only - http://bmjopen.bmj.com/site/about/guidelines.xhtml

**ILD Type, (no. (%))**<br>• IPF<br>• CTD-ILD<br>• CHP	18 (31.0)<br>28 (48.3)<br>12 (20.7)	8 (32)<br>15 (60)<br>2 (8)	10 (30.3)<br>13 (39.4)<br>10 (30.3)	0.10
**ILD-GAP index,**<br>(number (%))<br>• 0-1<br>• 2-3<br>• 4-5<br>• >5	24 (41.4)<br>20 (34.5)<br>10 (17.2)<br>2 (3.4)	12 (48)<br>9 (36)<br>4 (16)<br>0 (0)	12 (48)<br>11 (33.3)<br>6 (18.2)<br>2 (6.1)	0.72§
**BMI (kg/m²)**	27.3 (4.6)	27.5 (3.8)	27.2 (5.1)	0.84
**Waist/Hip ratio**	0.9 (0.1)	0.9 (0.1)	0.9 (0.1)	0.60
**Fat (%)**	29.9 (8.6)	29.9 (9.3)	30.1 (8.1)	0.95
**FVC (%,predicted)**	79.1 (21.8)	85.8 (24.0)	73.8 (18.8)	0.11
**DLCO (%, predicted)**	47.0 (14.7)	53.9 (13.1)	41.7 (13.9)	0.002
**Comorbidities (no. (%))**<br>• Cardiac<br>• Hypertension<br>• Diabetes<br>• High Cholesterol	21 (36.2)<br>23 (39.7)<br>15 (25.9)<br>12 (20.7)	8 (32)<br>9 (36)<br>7 (28)<br>7 (28)	13 (39.4)<br>14 (42.4)<br>8 (24.2)<br>5 (15.2)	0.56<br>0.62<br>0.75<br>0.23
**Prednisolone medication**<br>**(no. (%))**<br>• < 5mg<br>• 5-10 mg<br>• >10 mg	19 (32.8)<br>19 (32.8)<br>3 (5.17)	9 (36)<br>5 (20)<br>2 (8)	10 (30.3)<br>14 (42.4)<br>1 (3)	0.34§

For peer review only

**Smoking status (no. (%))**<br>• Current smoker<br>• Former smoker<br>• Never smoked<br>• Missing records	13 (22.4)<br>9 (15.5)<br>33 (56.9)<br>3	5 (20)<br>6 (24)<br>13 (52)<br>1	8 (24.2)<br>3 (9.1)<br>20 (60.6)<br>2	0.31§
**Pack year history**	8.9 (15.9)	10.7 (16.5)	7.4 (15.6)	0.50
Median/IQR interval	0(0, 10)	0(0, 20)	0(0, 4.5)	
Missing	7	3	5	
**6MWD (m)**	371.7 (122.2)	380.8 (139.8)	364.6 (108.3)	0.65
6MWD Missing	6	2	4	
**SpO2 in room air (%)**	96 (1.5)	96.5 (1.8)	96 (1.5)	0.3
Missing	6	2	4	
**No. on LTOT**	3	1	2	n/a
**SNIP (cmH_2O)**	94.8 (24.3)	96.8 (25.9)	93.1 (24.5)	0.61
Missing	9	3	6	
**PImax (cmH_2O)**	91.5 (30.4)	91.1 (34.2)	91.8 (27.4)	
Missing	8	2	6	
**PEmax (cmH_2O)**	106.6 (32.0)	114.4 (40.3)	100.7 (23.5)	0.22
Missing	23	10	13	
**Handgrip (dominant hand) (kg)**	25.8 (10.2)	27.2 (11.2)	24.8 (9.4)	0.42
Handgrip Missing	4	2	2	
**Quads extension (dominant side) (kg)**	18.3 (5.5)	18.7 (6.2)	18 (5.0)	0.65
Missing	2	0	2	
**Hip flexion (dominant side) (kg)**	15.6 (4.6)	15.3 (5.1)	15.9 (4.3)	0.41
Missing	1	0	1	
**HAD-A**	6.3 (4.2)	6.2 (4.6)	6.3 (3.9)	0.88
Missing	1	0	1	
**HAD-D**	5.5 (3.7)	5.8 (3.2)	5.2 (4.0)	0.28
Missing	1	0	1	

For peer review only - http://bmjopen.bmj.com/site/about/guidelines.xhtml

**SGRQ-I Total score**	46.6 (21.2)	43.3 (20.3)	49.3 (21.8)	0.29
Missing	1	0	1	

Data are means (SD) unless otherwise stated. The p-value tests the general null hypothesis of similarity between the randomized groups. The methods include t-tests (equal or unequal variances), Kruskal- Wallis and chi-squared (Fisher exact) as appropriate according to the nature of the variables. All tests have been carried out on the complete data only and missing data were not considered as a separate category. §= p value was calculated considering the whole (balanced) distribution.

Feasibility of HIIT compared to MICT

HIIT was well tolerated in a circuit-based clinical PR programme but required closer supervision to monitor participants' target heart rate and avoid desaturation below 85%. There were no adverse events from HIIT during or after the PR programme although attrition in the HIIT group was higher (42% compared to 28% in the MICT group) this did not appear to be due to a specific factor that could be directly attributed to the type of programme (Figure 1).

Change in exercise capacity, respiratory and peripheral muscle strength and health status

The change in outcomes from baseline at all assessment points are shown in Figure 2 and suggest a non-linear trend. This was then tested using linear and non-linear models and it was found that a quadratic model provided a significantly better fit than a linear model. The 6MWD increased over time from baseline in both groups, after which it tended to return towards starting levels ($p<0.001$ for a quadratic term of change over time), Figure 3. The p value for the group-time interaction term was $p=0.006$ suggesting that the trajectories differed between groups. The HIIT group peaked quicker, around 4 months compared to around 5 months with MICT (Figure 3). The models also suggested that the change in 6MWD values were smaller with HIIT (Table 2, Figure 2); mean change at peak was 51.6m (95% CI 29.2, 73.9) in the MICT group compared to 26.3m (3.5, 49.1) with HIIT. The picture was the same using the estimates at 2 and 8 months

For peer review only - http://bmjopen.bmj.com/site/about/guidelines.xhtml

(Table 2, Figure 2). Age was inversely associated with the average 6MWD values in both groups, but the time course was similar across ages (Fig. 3).

Figure 2 and 3 around here

Table 2: Average predictions for main outcomes values at the main time points during the trial and for changes from baseline (actual and adjusted for age)

For peer review only

For peer review only - http://bmjopen.bmj.com/site/about/guidelines.xhtml

Outcome	Time	Group data Mean (95% CI)		Change from baseline Mean (95% CI)		Change from baseline adjusted for age Mean (95% CI)	
		Control (MICT)	Intervention (HIIT)	Control (MICT)	Intervention (HIIT)	Control (MICT)	Intervention (HIIT)
6MWD (m)	**Baseline**	382 (336, 429)	372 (331, 412)				
	**2 months**	433 (386 480)	411 (370, 453)	54.7 (32., 76.9)	38.6 (15.95, 61.17)	53.2 (33.66, 72.70)	36.3 (16.42, 56.06)
	**8 months**	428 (378, 476)	374 (331, 418)	48.2 (24.0, 72.4)	2.64 (-23.4, 28.6)	46.6 (26.5, 66.7)	.06 (-21.95, 22.08)
	**Peak value**	460 (411, 508)	425 (382, 468)	51.6 (29.2, 73.9)	26.29 (3.5, 49.1)	50.02 (31.2, 68.8)	23.88 (4.5, 43.3)
	**Peak time-months**	4.87 (4.43, 5.30)	4.05 (3.62, 4.48)				
SNIP (cmH_2O)	**Baseline**	95 (85, 105)	92 (82, 101)				
	**2 months**	104 (94, 115)	98 (88, 108	9.53 (2.02, 17.04)	10.07 (2.28, 17.85)	9.21 (1.98, 16.4)	9.40 (1.88, 16.92)
	**8 months**	101 (90, 112)	86.9 (76, 98)	8.08 (-0.32, 16.48)	-5.73 (-15.03, 3.56)	7.38 -1.05774 15.81	-6.35 -15.64376 2.95
	**Peak value**	108 (97, 120)	99 (89, 110)	8.89 (2.33, 15.44)	6.01 (-1.10, 13.12)	8.39 (2.00, 14.78)	5.36 (-1.53, 12.25)
	**Peak time-months**	4.57 (3.72, 5.43)	3.54 (2.57, 4.51)				
QU	**Baseline**	18.8 (16.7, 20.9)	17.6 (15.7, 19.5)				
	**2 months**	21.6 (19.4, 23.8)	20.1 (18.1, 22.1)	3.37 (1.97, 4.78)	2.44 (1.09, 3.78)	3.33 (1.91, 4.74)	2.39 (1.03, 3.74)

For peer review only - http://bmjopen.bmj.com/site/about/guidelines.xhtml

	**8 months**	22.7 (20.2, 25.6)	20.6 (18.2, 23.0)	4.10 (2.48, 5.72)	3.16 (1.55, 4.78)	4.06 (2.46, 5.65)	3.12 (1.52, 4.71)
	**Peak value**	23.5 (21.0, 26.0)	21.7 (19.3, 24.1)	3.82 (2.46, 5.17)	2.83 (1.53, 4.13)	3.77 (2.43, 5.11)	2.78 (1.49, 4.07)
	**Peak time-months**	5.65 (4.39,6.91)	5.27 (4.19, 6.36)				
**SGRQ-I (Total score)**	**Baseline**	43.2 (35.4, 51.1)	48.8 (41.9, 55.7)				
	**2 months**	36.6 (28.5, 44.7)	43.0 (35.8, 50.2)	-6.57 (-11.81, -1.33)	-3.75 (-8.92, 1.42)	-6.39 (-11.62, -1.17)	-3.54 (-8.71, 1.63)
	**8 months**	40.3 (31.8, 48.8)	49.2 (41.3, 57.1)	-2.57 (-8.06, 2.92)	2.15 (-3.39, 7.68)	-2.40 (-7.89, 3.09)	2.37 (-3.18, 7.93)
	**Min value**	33.9 (25.1, 42.6)	41.1 (33.1, 49.1)	-4.99 (-9.62, -0.36)	-1.84 (-6.51, 2.83)	-4.82 (-9.44, -0.19)	-1.63 (-6.31, 3.05)
	**Min time-months**	4.37 (3.68, 5.07)	3.94 (3.24, 4.65)				

The quadratic term was significant for all secondary outcomes ($p=0.0014$ for SNIP, $p= 0.004$ for QUADS-DOM and $p= 0.0014$ for SGRQ-I), (Figure 3).

SNIP values peaked earlier than 6MWD values in both groups by approximately 2 weeks (Figure 3). QUADS-DOM values were maintained above baseline levels in both groups (Figure 2) and showed a similar time course of change.

There was no evidence for a difference between treatment groups for the SGRQ-I values. The time to reaching the greatest improvement in SGRQ-I score was shorter in the HIIT group, but the changes in the HIIT group were of smaller magnitude at all time-points (Supplementary Tables 1 and 2).

For peer review only - http://bmjopen.bmj.com/site/about/guidelines.xhtml

Effects of HIIT on different aetiology ILD

Our models included 3-way interactions between time, intervention (HIIT or MICT) and clinical group, to predict changes from baseline in the combined fibrosis (IPF and CHP group) and CTD-ILD groups. The patterns of change are consistent with the primary analysis – i.e. that whilst the HIIT group peaked faster than the MICT group, the level of improvement was less (Supplementary Tables 1 and 2 and Supplementary Figures 1-4).

The effect of baseline DLCO on HIIT

Despite stratified randomisation, DLCO seemed to differ between intervention groups. (Table 1). We therefore performed additional, post-hoc analyses to examine its possible effect on outcomes. Based on these data, there was no evidence to suggest that DLCO further explained differences in the outcomes after accounting for time, age at the recruitment, intervention and clinical groups and their interactions (see Supplementary file 3).

Discussion

To our knowledge, this is the first study to investigate the feasibility and potential efficacy of HIIT in the context of a circuit-based clinical PR programme for ILD and compare it with MICT. It demonstrated the feasibility of HIIT, in PR for ILD patients and provided preliminary data on the exercise mode more likely to maintain benefits 6 months after programme completion. In addition, we identified the time of peak benefit and time course of subsequent deterioration.

HIIT proved to be feasible and well tolerated but required closer supervision and careful instructions for patients to continue this mode of training than MICT. The attrition rate was higher in the HIIT group

For peer review only - http://bmjopen.bmj.com/site/about/guidelines.xhtml

compared to the MICT group and more contact was needed in the 6-month period to ensure the HIIT mode of training met the protocol requirements.

The preliminary efficacy of HIIT over MICT was tested using random effects models. Their aim was to understand the average variation of the four key outcomes through time across groups to answer five main questions: a) How do these measurements change over time?; b) Does the benefit differ between groups?; c) How sustainable is the benefit? d) Does the attrition rate differ between groups? e) What is the influence of age? The study was primarily designed to compare the two treatment groups, but an exploratory analysis was performed to look for differences between clinical groups, although this resulted in small numbers in the subgroups.

Contrary to our initial hypothesis, our data showed no evidence of greater benefit with HIIT compared to that achieved with MICT in exercise capacity, respiratory and peripheral muscle function or health status in the medium-term. The study was designed as a pilot to test the potential for a trial of HIIT in a larger, definitive study, but we are forced to conclude that a study of the same design may not be worthwhile.

At 2 months, both groups improved > 34 meters in the 6MWD, which is the reported minimal clinical important difference (MCID) in IPF patients [32, 36, 37], adding to existing evidence on the benefits of PR in ILD [11, 32-33, 38-42]. However, although the 6MWD improved earlier in the HIIT group (peaking at 4 months instead of 5 months) differences from baseline were consistently lower and not as well maintained at 8 months compared with the MICT group. A similar pattern emerged when the effect of age was added to the model. Age is an important factor in indices associated with mortality risk in ILD [3, 4] and our results showed lower 6MWD with greater age. Despite this age effect, PR had a positive effect in all age groups regardless of baseline levels, contrary to previous studies reporting greater improvement in patients with lower baseline 6MWD [11, 37, 41, 43]. However, HIIT did not offer additional or preferential benefits to any specific age group and tended to decline below baseline levels at 8 months in the older group. In contrast, the benefit seen in the older MICT group was maintained above baseline levels.

For peer review only - http://bmjopen.bmj.com/site/about/guidelines.xhtml

In addition to functional exercise capacity, patients improved quadriceps muscle strength post-PR but similarly, there was no difference between treatment groups. Age-adjusted results for quadriceps force were less variable than for 6MWD and benefits were well maintained above baseline in both groups. The modelling suggested that quadriceps force peaked a little later than other outcomes at 5.5 months in the MICT group and at 5 months at the HIIT group. Our results support the inclusion of strength training in ILD-PR programmes and suggest that it may explain why some studies showed greater improvement and maintenance in functional capacity [33] while others that focused on endurance training did not [11]. The time course of peak improvement and subsequent decline in quadriceps strength did not follow the pattern seen with 6MWD, in either group. Quadriceps strength has been shown to deteriorate in line with ageing and severity in other chronic respiratory disorders [44] and has been identified as an important predictor of mortality in COPD [45]. Skeletal muscle atrophy has also been identified in advanced ILD [46]. Our ILD group, which included people in advanced stage (as defined by the ILD-GAP score > 5, Table 1) showed improvement in mean quadriceps force post-PR in both groups and across all age groups. We found no evidence that older individuals benefited more from PR.

Respiratory muscle strength was preserved at baseline (mean SNIP, MIP and MEP > 70 cmH_2O in both groups) as previous described [47, 48] and improved post-PR in both groups even though there was no inclusion of specific inspiratory muscle training in our programme. In addition, the SNIP followed a similar pattern over time to 6MWD and peaked approximately two weeks earlier than the 6MWD in both the HIIT and MICT group. Although there was no additional benefit with HIIT, our results suggest that SNIP may be more useful in monitoring changes following PR than quadriceps force in ILD patients. In support of this conclusion, Mendoza and colleagues [47] showed that inspiratory muscle strength correlated better with 6MWD than quadriceps twitch force in patients with fibrotic idiopathic interstitial pneumonia. Although our ILD group was more mixed and included CTD-ILD patients, our results confirm that respiratory muscle strength appears preserved in ILD and that inspiratory muscle strength correlates well with the 6MWD. Our

For peer review only - http://bmjopen.bmj.com/site/about/guidelines.xhtml

study is the first to show that inspiratory muscle strength changes in tandem with the 6MWD following PR, regardless of the chosen mode of training.

Health status, as defined by the SGRQ-I and HAD scale improved post-PR and was maintained at 8 months, but again, there was no evidence of superiority of HIIT over the MICT group (Table 1- online supplement).

Effects of HIIT on different aetiology ILD

Random effect models were also created based on ILD type (clinical subgroups). We combined the IPF and CHP subgroups into the combined ‘Fibrosis’ clinical group. This was due to smaller numbers of IPF participants at the end of the study which did not allow for independent evaluation of this subgroup.

The effects of HIIT on ILD of different aetiology were consistent with our primary analysis, i.e. that HIIT peaked faster and the level of improvement was less. This result seemed to be largely attributable to the fibrosis group, which peaked fastest but showed the lowest improvement from baseline compared to the CTD-ILD group. Similar findings were observed for the other outcomes, again showing lack of preferential benefit from HIIT in any subgroup. Whilst the CTD-ILD subgroup improved in most variables, the HIIT mode was not superior to MICT.

Limitations

The study has limitations. First, although our retrospective analysis showed no evidence that baseline DLCO had an effect on outcomes following our training programme (Supplementary file 3), the effect may have been concealed by the size of this pilot study. Imbalance in randomisation can happen by chance in clinical trial settings, particularly in pilot/feasibility studies [49]. However, DLCO did not affect PR benefits in a previous study [50], but future definitive studies may need to consider controlling this factor at randomisation.

Second, we did not use supplementary oxygen during PR for participants other than for those already on long term oxygen therapy (LTOT). Although promising benefits have been reported with acute oxygen

For peer review only - http://bmjopen.bmj.com/site/about/guidelines.xhtml

supplementation during exercise in IPF patients [51], there is still insufficient evidence about medium to long-term benefits in ILD patients [34] For our study, we felt it was important to train participants to recognise the time it took before desaturation occurred, so that they were able to continue that pattern of training and monitor themselves when unsupervised in community. Participants already on LTOT continued to exercise with oxygen. However, none of the three participants on LTOT finished PR, regardless of group allocation, supporting previous reports that PR may not be appropriate for them [52]. Future studies need to explore different models of training for these cases, such as home-based programmes.

Finally, the number of IPF participants who remained in the study was small and did not allow us to investigate the effect of HIIT over time in this particular group.

Implications for future definitive studies

This pilot, enabled us to explore the effect of mode of training on an ILD-PR programme of 8-weeks duration followed by community-based training for 6 months. Our programme duration reflects current UK-based clinical programmes and showed improvements that were maintained at 8 months and were similar to a recent study of a much longer PR programme duration [33]. Although we conclude that there is no evidence to support the use of HIIT in a future definitive study of similar design to this pilot, our results suggest that HIIT may have a role when quicker improvements are required, such as in pre-operative rehabilitation for ILD and that HIIT is feasible to be investigated further in a larger, definitive study of different design. Our results also provide valuable information on the time point where 6MWD and other variables start to deteriorate post-PR and therefore, an estimate of time when additional PR support may be needed, thus optimising PR provision for ILD patients.

Conclusions

For peer review only

For peer review only - http://bmjopen.bmj.com/site/about/guidelines.xhtml

In the current study, HIIT was found to be feasible and improved exercise capacity in an ILD-PR programme, but this exercise modality showed no evidence of superiority over MICT.Indeed, our findings suggest that it may be less effective and may require closer monitoring to avoid exercise-induced desaturation so, until further evidence becomes available, MICT appears to be more appropriate in the general clinical setting.

Author Contributions: Concept and Design of Study: DN, PJ, FC, RA, IC-S; Acquisition of Data: DN, CL, IMcN ; Statistical Analytical Strategy: ICS, Analysis of Data: DN, IC-S; Drafting of Manuscript: DN, IC-S, PJ ; Revision of manuscript critically for important intellectual content: All authors; Approval of final manuscript: All authors

Competing interests: In the last 36 months:

DN, IMN and CYL declare no financial interests.

ICS- has had grants from NIHR, Astra-Zeneca and BHF and is a statistical expert for the Health Research Authority

FC- has received speaker fees and financial sponsorship for conducting meetings by Boehringer-Ingelheim and Roche

RA- has received lecture fees from Pfizer and Novartis

PJ- owns GlaxoSmithKline shares

Ethics statement: Ethical approval was secured through the South East Coast-Surrey NRES committee (REC reference: 14/LO/0149) on the 14th February 2014.

Funding statement: The study was funded by the National Institute for Health Research (NIHR) Research for Patient Benefit (RfPB) Programme (Grant Reference Number PB-PG-1112-29067). The views expressed are those of the authors and not necessarily those of the NHS, the NIHR or the Department of Health.

Study registration and timeline: The trial protocol was pre-registered with the ISRCTN registry (ISRCTN55846300). Study duration: 02/05/2014 to 30/09/2016 (last participant assessment), Analysis completed: 2017.

Data sharing statement: Data are available upon reasonable request. All relevant data are presented in this paper; and more information can be provided upon reasonable request from the corresponding author

Acknowledgements: The authors would like to thank all ILD patients and particularly the St George's Interstitial Lung Disease Support Group for their participation and input in this study.

For peer review only

List of figures:

Figure 1: CONSORT diagram of participants' flow in the study. ILD-PR= pulmonary rehabilitation programme tailored for interstitial lung disease patients, MICT= moderate intensity continuous training, HIIT= high intensity interval training, IPF= idiopathic pulmonary fibrosis, CTD-ILD= connective tissue disease related interstitial lung disease, CHP= chronic hypersensitivity pneumonitis.

Figure 2: Change from baseline in 4 key outcomes by intervention group over time.

Figure 3: Age-related variations in the 4 key outcomes (6MWD, SNIP, QUADS-DOM (Quadriceps strength) and SGRQ-I total score) per training group using random effects models. The vertical line indicates the inflection point (i.e. highest or lowest values) in each age group. The ages are the mean values in each tertile

Supplementary Figure 1: Changes in key outcomes by intervention and ILD clinical groups

Supplementary Figure 2: Random effects models for the 6MWD by intervention (HIIT vs MICT) and ILD clinical subgroup (Fibrosis vs CTD-ILD)

Supplementary Figure 3: Random effects models for SNIP by intervention (HIIT vs MICT) and ILD clinical subgroup (Fibrosis vs CTD-ILD)

Supplementary Figure 4: Random effects models for quadriceps strength-dominant side (Quad_dom) by MICT vs HIIT and ILD clinical subgroups.

For peer review only

References

1. Travis WD, Costabel U, Hansell DM, et al. ATS/ERS Committee on Idiopathic Interstitial Pneumonias. An official American Thoracic Society/European Respiratory Society statement: Update of the international multidisciplinary classification of the idiopathic interstitial pneumonias. *Am J Respir Crit Care Med* 2013 Sep 15; 188 (6): 733-48.
2. Wells AU and Hirani N. BTS Interstitial lung disease guideline: the British Thoracic Society in collaboration with the Thoracic Society of Australia and New Zealand and the Irish Thoracic Society. *Thorax* 2008; 63 (Suppl V): v1- v58.
3. Ley B, Ryerson CJ, Vittinghoff E, et al. A multidimensional index and staging system for idiopathic pulmonary fibrosis. *Ann Intern Med.* 2012; 156 (10): 684-91.
4. Ryerson CJ, Vittinghoff E, Ley B, et al. Predicting survival across chronic Interstitial Lung Disease- The ILD-GAP model. *Chest* 2014; 145 (4): 723-28.
5. Dowman L, Hill CJ, May A, Holland AE. Pulmonary rehabilitation for interstitial lung disease. *Cochrane Database Syst Rev.* 2021 J; 2 (2):CD006322.
6. Cottin V, Wollin L, Fisher A, et al. Fibrosing interstitial lung diseases: knowns and unknowns. *Eur Respir Rev* 2019; 28: 180100 [https://doi.org/10.1183/16000617.0100-2018].
7. Leong S W, Bos S, Lordan J L, et al. Lung transplantation for interstitial lung disease: evolution over three decades. *BMJ Open Resp Res* 2023; 10: e001387. doi:10.1136/bmjresp-2022-001387.
8. Kapnadak SG, Raghu G. Lung transplantation for interstitial lung disease. *Eur Respir Rev* 2021; 30: 210017 [DOI: 10.1183/16000617.0017-2021]
9. Spruit M. A., Singh S., Garvey C, et al. An Official American Thoracic Society/European Respiratory Society statement: Key concepts and advances in Pulmonary Rehabilitation. *Am J Resp Crit Care Med* 2013; Vol 188 (no 8): e13-e64.
10. Holland AE, Hill CJ, Conron M, *et al*. Short term improvement in exercise capacity and symptoms following exercise training in interstitial lung disease. *Thorax* 2008; 63: 549-554.
11. Dowman L. M, McDonald C.F., Hill C.J, *et al.* The evidence of benefits of exercise training in interstitial lung disease: a randomised controlled trial. *Thorax* 2017; 0: 1-10.
12. American College of Sports Medicine (ACSM)Guidelines for exercise testing and prescription. (2022) 11th edition. Wolters Kluwer Health
13. Helgerud J, Høydal K, Wang E, Karlsen T, Berg P, Bjerkaas M, et al. Aerobic high-intensity intervals improve VO_2max more than moderate training. *Med Sci Sports Exerc* 2007; 39 (4):665–71.
14. Levinger et al. What Doesn't Kill You Makes You Fitter: A Systematic Review of High-Intensity Interval Exercise for Patients with Cardiovascular and Metabolic Diseases. *Clinical Medicine Insights: Cardiology* 2015:9 53–63.
15. Arena R, Myers J, Forman DE, Lavie CJ, Guazzi M. Should high-intensity-aerobic interval training become the clinical standard in heart failure? *Heart Fail Rev*. 2013; 18 (1): 95–105.
16. Shiraev T and Barclav G. Evidence based exercise- Clinical benefits of high intensity interval training. *Aust Fam Physician* 2012; 41 (12): 960-962.
17. Wisloff U, Stovlen A, Loennechen JP, et al. Superior cardiovascular effect of aerobic interval training versus moderate continuous training in heart failure patients. *Circulation* 2007; 115: 3086-3094.

For peer review only - http://bmjopen.bmj.com/site/about/guidelines.xhtml

18. Vogiatzis I, Gerasimos Terzis, Serafeim Nanas, *et al*. Skeletal muscle adaptations to interval training in patients with advanced COPD. *Chest* 2005; 128: 3838-3845.
19. Vogiatzis I, Nanas S, Kastanakis E, et al. Dynamic hyperinflation and tolerance to interval exercise in patients with advanced COPD. *Eur Respir J* 2004; 24: 385–390.
20. Kortianou EA, Nasis IG, Spetsioti ST, et al. Effectiveness of interval exercise training in patients with COPD. *Cardiopulm Phys Ther J* 2010; 21: 12–19.
21. Beauchamp M. A, Nonoyama M., Goldstein R. S., et al. Interval versus continuous training in individuals with chronic obstructive pulmonary disease- a systematic review. *Thorax* 2010; 65: 157-164.
22. Gloeckl R, Halle M, Kenn K. Interval versus continuous training in lung transplant candidates: a randomized trial. *J Heart Lung Transplant* 2012; 31: 934–941.
23. Nikoletou D, Lech C. Y., MacNaughton I.S, Chis-Ster I., Chua F., Aul R., Jones P.. High intensity interval versus moderate intensity continuous training in pulmonary rehabilitation for patients with interstitial lung disease. - American Thoracic Society Conference- B109. HIGHLIGHTS AND ADVANCES IN PULMONARY REHABILITATION. May 2016, A4520-A4520 San Francisco, USA.
24. Manali, E.D., Lyberopoulos, P., Triantafillidou, C. *et al.* MRC chronic Dyspnea Scale: Relationships with cardiopulmonary exercise testing and 6-minute walk test in idiopathic pulmonary fibrosis patients: a prospective study. *BMC Pulm Med* **10**, 32 (2010). https://doi.org/10.1186/1471-2466-10-32
25. Borg G: Perceived exertion as an indicator of somatic stress. *Scand J Rehabil Med*. 1970, 2: 92-98.
26. Borg GA: Psychophysical bases of perceived exertion. *Med Sci Sports Exerc.* 1982, 14: 377-381
27. ATS statement: guidelines for the six-minute walk test. *Am J Respir Crit Care Med* 2002; 166: 111–17.
28. Yorke J, Jones P, Swigris J J. Development and validity testing of an IPF-specific version of the St George's Respiratory questionnaire. *Thorax* 2010; 65: 921-926.
29. Zigmond AS and Snaith RP: The Hospital Anxiety And Depression Scale. *Acta Psychiatr Scand* 1983, 67:361-70
30. PA Harris, R Taylor, BL Minor, V Elliott, M Fernandez, L O'Neal, L McLeod, G Delacqua, F Delacqua, J Kirby, SN Duda, REDCap Consortium, The REDCap consortium: Building an international community of software partners. *J Biomed Inform. 2019 May 9 [doi: 10.1016/j.jbi.2019.103208]*
31. Resources for pilot and feasibility studies. Available from: https://pilotandfeasibilitystudies.qmul.ac.uk/ resources/.
32. Holland AE, Hill CJ, Connon M, *et al*. Short term improvement in exercise capacity and symptoms following exercise training in interstitial lung disease. *Thorax* 2008; 63: 549-554.
33. Perez-Bogerd S., Wuyts W, Barbier V, Demeyer H, Van Muylem A, Janssens W, Troosters T. Short and long-term effects of pulmonary rehabilitation in interstitial lung diseases: a randomised controlled trial. *Respiratory Research* 2018; 19 (1): 182.
34. Bell EC, Cox NS, Goh N, et al. Oxygen therapy for interstitial lung disease: a systematic review. *Eur Respir Rev* 2017: 26: 160080 [https://doi.org/10.1183/16000617.0080-2016]
35. Sterne J A C, White I R, Carlin J B, Spratt M, Royston P, Kenward M G et al. Multiple imputation for missing data in epidemiological and clinical research: potential and pitfalls. *BMJ* 2009; 338: b2393 doi: 10.1136/bmj.b2393.
36. Holland AE, Hill CJ, Connon M, *et al*. Small changes in six-minute walk distance are important in diffuse parenchymal lung disease. *Respir Med* 2009; 103:1430-5.
37. du Bois RM, Weycker D, Albera C, et al. Six-minute walk test in idiopathic pulmonary fibrosis; test validation and minimal clinically important difference. *Am J Respir Crit Care Med* 2011; 183: 1231-1237.

For peer review only - http://bmjopen.bmj.com/site/about/guidelines.xhtml

38. Tonelli R, Cocconcelli E, Lanini B, Romagnoli I et al. Effectiveness of pulmonary rehabilitation in patients with interstitial lung disease of different etiology; a multicentre prospective study. *BMC Pulmonary Medicine* 2017; 17: 1-9.
39. Nishiyama O, Kondoh Y, Kimura T, Kato K, Kataoka K, Ogawa T, Watanabe F, Arizono S, Nishimura K, Taniguchi H. Effects of pulmonary rehabilitation in patients with idiopathic pulmonary fibrosis. *Respirology* 2008;13:394–9.
40. Vainshelboim B, Oliveira J, Yehoshua L, Weiss I, Fox BD, Fruchter O, Kramer MR. Exercise training-based pulmonary rehabilitation program is clinically beneficial for idiopathic pulmonary fibrosis. *Respiration*. 2014;88:378–88.
41. Ferreira A, Garvey C, Connors GL, Hilling L, Rigler J, Farrell S, Cayou C, Shariat C, Collard HR. Pulmonary rehabilitation in interstitial lung disease: benefits and predictors of response. *Chest* 2009;135:442–7.
42. Jackson RM, Gomez-Marin OW, Ramos CF, Sol CM, Cohen MI, Gaunaurd IA, Cahalin LP, Cardenas DD. Exercise limitation in IPF patients: a randomized trial of pulmonary rehabilitation. *Lung* 2014;192:367–76.
43. Ryerson CJ, Cayou C, Topp F, et al. Pulmonary rehabilitation improves long-term outcomes in interstitial lung disease: a prospective cohort study. *Respir Med* 2014;108:203–10.
44. Seymour JM, Spruit MA, Hopkinson NS, et al. The prevalence of quadriceps weakness in COPD and the relationship with disease severity. *Eur Respir J* 2010; 36(1): 81–88.
45. Swallow EB, Reyes D, Hopkinson NS, et al. Quadriceps strength predicts mortality in patients with moderate to severe chronic obstructive pulmonary disease. *Thorax* 2007; 62(2): 115–120.
46. Mendes P, Wicherson L, Helm D, et al. Skeletal muscle atrophy in advanced interstitial lung disease. *Respirology* 2015; 20: 953-959.
47. Mendoza L, Gogali A, Shrikrishna D, Cavada G, et al. Quadriceps strength and endurance in fibrotic idiopathic interstitial pneumonia. *Respirology* 2014; 19: 138-143.
48. de Troyer A, Yernault JC, Inspiratory muscle force in normal subjects and patients with interstitial lung disease. *Thorax* 1980; 35: 92-100.
49. Roberts C, Torgerson D J. Baseline imbalance in randomised controlled trials. *BMJ* 1999; 319: 185.
50. Tonelli R, Cocconcelli E, Lanini B, et al. Effectiveness of pulmonary rehabilitation in patients with interstitial lung disease of different etiology; a multicentre prospective study. *BMC Pulmonary Medicine* 2017; 17: 130-139.
51. Dowman LM, McDonald CF, Bozinovski S, Vlahos R, Gillies R, Pouniotis D, Hill CJ, Goh NSL, Holland AE. Greater endurance capacity and improved dyspnoea with acute oxygen supplementation in idiopathic pulmonary fibrosis patients without resting hypoxaemia. *Respirology* 2017; 22: 957-964.
52. Johnson-Warrington V, Williams J, Bankart J, Steiner M, Morgan M, Singh S. Pulmonary rehabilitation and Interstitial lung disease: Aiding the referral decision. *J Cardiopulm Rehabil Prev*2013; 33 (3): 189-195.

For peer review only - http://bmjopen.bmj.com/site/about/guidelines.xhtml

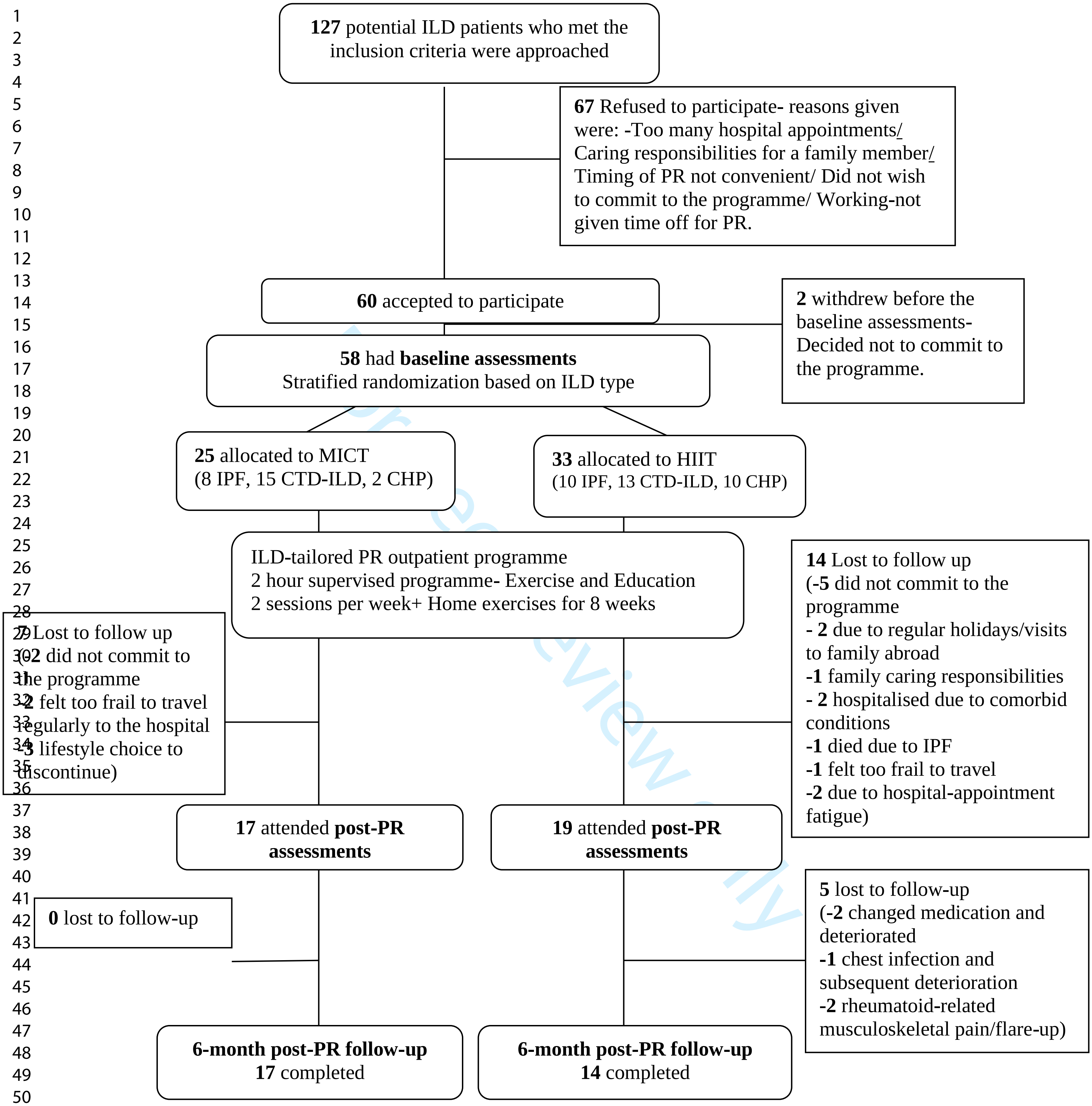

Figure 1: CONSORT diagram of participants' flow in the study. ILD-PR= pulmonary rehabilitation programme tailored for interstitial lung disease patients, MICT= moderate intensity continuous training, HIIT= high intensity interval training, IPF= idiopathic pulmonary fibrosis, CTD-ILD= connective tissue disease related interstitial lung disease, CHP= chronic hypersensitivity pneumonitis.

For peer review only - http://bmjopen.bmj.com/site/about/guidelines.xhtml

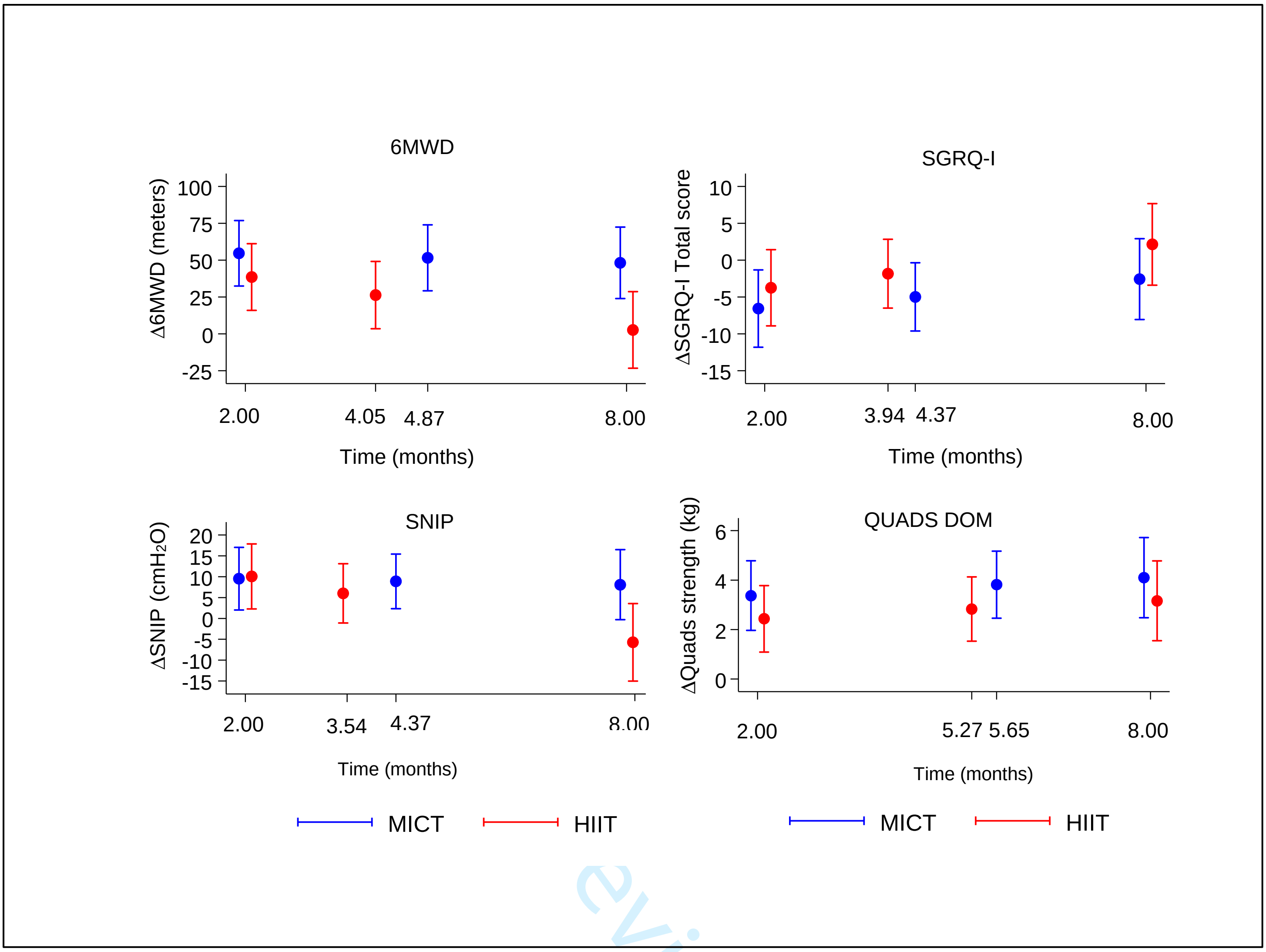

Figure 2: Change from baseline in 4 key outcomes by intervention group over time.

eview only

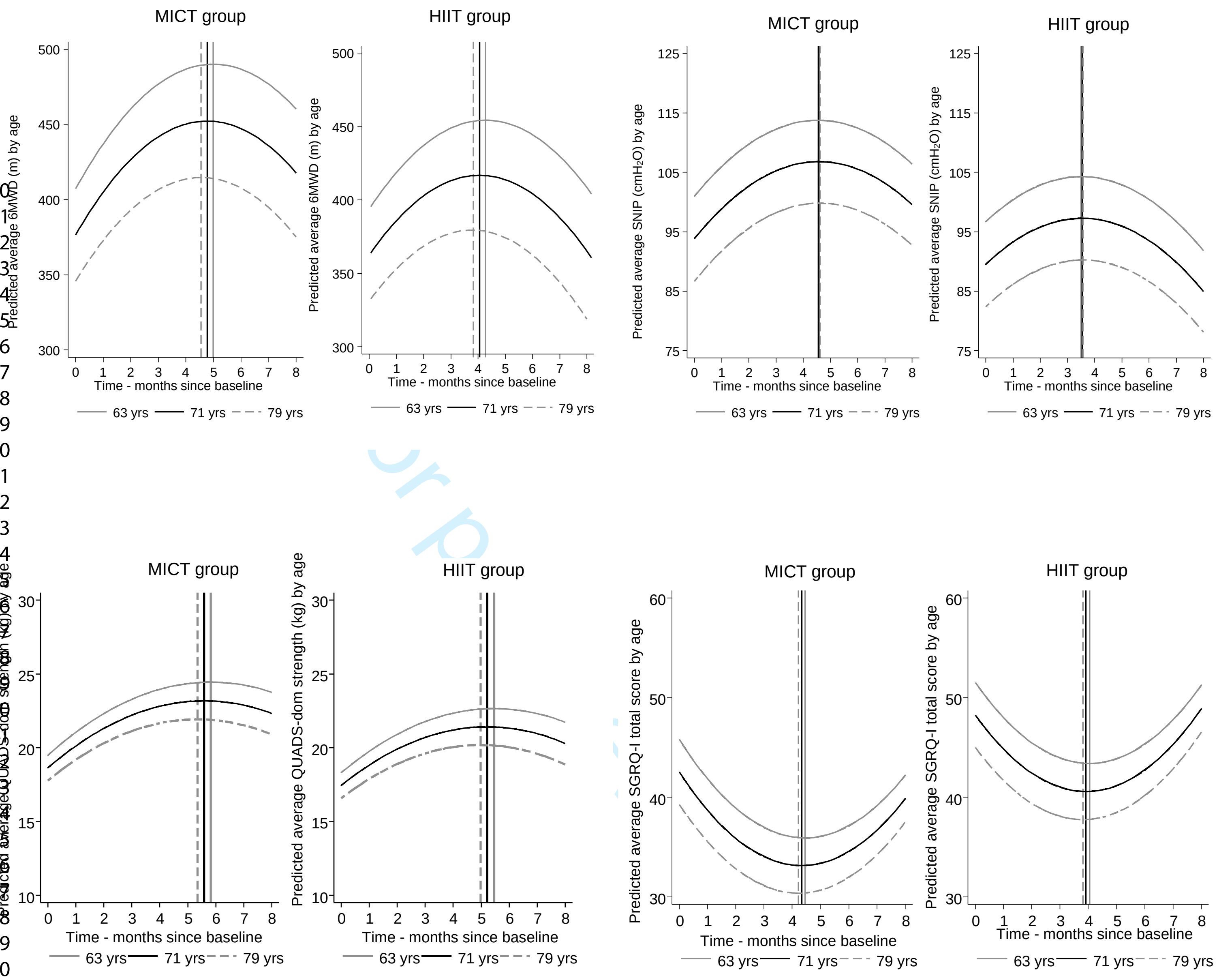

Figure 3: Age-related variations in the 4 key outcomes (6MWD, SNIP, QUADS-DOM (Quadriceps strength) and SGRQ-I total score) per training group using random effects models. The vertical line indicates the inflection point (i.e. highest or lowest values) in each age group. The ages are the mean values in each tertile

Supplementary Table 1- Descriptive values at each assessment point

Table 1: Descriptive values at each assessment point

	Control group (MICT) Mean (SD)			Intervention group (HIIT) Mean (SD)		
	**Baseline N= 25**	**2M (Post-PR) N=17**	**8M (6M post-PR) N= 17**	**Baseline N=33**	**2M (Post-PR) N=19**	**8M (6M post-PR) N=14**
**BMI (kg/m²)**	27.5 (3.8)	28.3 (4.1)	27.8 (3.9)	27.2 (5.1)	26.5 (4.9)	26.0 (4.7)
**Waist/Hip ratio**	0.9(0.1)	0.9 (0.1)	0.9 (0.0)	0.9 (0.1)	0.9 (0.1)	0.9 (0.1)
**%fat**	29.9 (9.3)	33.2 (9.8)	33.7 (8.7)	30.1 (8.1)	27.9 (10.7)	28.5 (9.6)
**FVC%,pred.**	85.8 (23.9)	88.0 (17.5)	88.7 (24.4)	73.8 (18.8)	68.4 (22.4)	71.3 (24.1)
**DLCO%, pred.**	53.9 (13.1)	53.6 (13.5)	56.5 (21.1)	41.7 (13.9)	44.9 (20.2)	44.8 (20.3)
**6MWD (m)**	380 (139.8)	453.9 (108.1)	437.4 (107.9)	364 (108.3)	434.3 (102.3)	421.9 (123.0)
**SNIP (cmH_2O)**	96.8 (25.9)	103.0 (21.04)	97.8 (21.4)	93.1 (24.5)	102.2 (34.1)	95.3 (29.5)
**PImax(cmH_2O)**	91.1(34.2)	104.3 (28.5)	104.9 (24.0)	91.8 (27.4)	92.8 (28.6)	95 (31.7)
**PEmax(cmH_2O)**	114.4 (40.3)	111.1 (33.3)	106.8 (34.5)	100.7 (23.5)	105.1 (24.2)	106.9 (24.7)
**Handgrip (kg)**	27.2 (11.2)	28.4 (8.4)	27.4 (9.3)	24.8 (9.4)	26.2 (11.4)	27.4 (11.5)
**Quads extension (dom) (kg)**	18.7 (6.2)	21.8 (5.0)	22.8 (6.1)	18.0 (5.0)	20.0 (5.6)	21.5 (5.4)
**Hip flexion (dom) (kg)**	15.3 (5.1)	17.9 (4.1)	18.5 (5.1)	15.9 (4.3)	16.5 (4.7)	18.1 (6.3)
**HAD-A**	6.2 (4.6)	4.8 (3.3)	5.1 (3.7)	6.4 (3.9)	6.4 (4.6)	7.2 (4.2)
**HAD-D**	5.8 (3.2)	5.0 (3.3)	5.2 (2.3)	5.2 (4.0)	5.1 (3.4)	6.6 (4.2)
**SGRQ-I Total score**	43.3 (20.3)	35.1 (15.3)	41.5 (19.3)	49.3 (21.8)	40.2 (16.7)	48.9 (21.6)

For peer review only

Supplementary Table 2- Average predictions for main outcomes at main time points

Table 2: Average predictions for main outcome values at the main time points during the trial, i.e. baseline, 2 months, 8 months and *changes from the baseline* at 2 months, 8 months and by the time they reach their max/min values. This set of predictions are made across intervention and ILD clinical groups, adjusting for age (hence predictions are made for patients of average age of 71 years). Estimated peak/saddle times are also displayed.

For peer review only

Supplementary Table 2- Average predictions for main outcomes at main time points

Outcome	Time	Control (MICT)				Intervention (HIIT)			
		Fibrosis		CTD-ILD		Fibrosis		CTD-ILD	
		Mean (95% CI) predicted value	Change from baseline	Mean (95% CI) predicted value	Change from baseline	Mean (95% CI) predicted value	Change from baseline	Mean (95% CI) predicted value	Change from baseline
6MWD	Baseline	415.84 (360.8 to 470.9))		393.89 (337.8 to 450.0)		367.94 (327.4 to 408.5)		358.88 (304.8 to 413.0)	
	2 months	464.63 (409.4 to 519.84)	45.5 (17.5 to 73.5)	442.92 (387.3 to 498.6)	55.4 (35.3 to 75.6)	401.22 (360.1 to 442.3)	28.9 (5.7 to 52.0)	410.63 (356.3 to 465.0)	38.3 (14.2 to 63.5)
	8 months	459.03 (398.5 to 519.6)	39.2 (11.2 to 67.2)	438.02 (381.6 to 494.5)	49.2 (28.8 to 69.6)	349.07 (304.9 to 393.3)	- 6.84 (-30.9 to 17.3)	413.93 (351.5 to 476.4)	3.1 (-23.5 to 29.7)
	Peak value	490.40 (432.7 to 548.1)	42.5 (15.2 to 69.8)	469.02 (412.6 to 525.5)	52.4 (33.1 to 71.8)	409.60 (366.8 to 452.4)	19.2 (-3.3 to 41.7)	440.80 (382.9 to 498.7)	20.4 (-4.2 to 45.1)
	Peak time-months	4.85 (4.1 to 5.6)		4.87 (4.4 to 5.4)		3.63 (3.1 to 4.2)		5.09 (4.3 to 5.9)	
SNIP	Baseline	99.94 (86.3 to 113.5)		90.69 (77.4 to 104.0)		96.92 (86.5 to 107.3)		77.55 (64.5 to 90.6)	
	2 months	106.89 (93.4 to 120.4)	14.3 (4.0 to 24.5)	100.33 (87.2 to 113.4)	10.9 (3.7 to 18.0)	102.38 (91.6 to 113.1)	8.6 (0.2 to 16.9)	85.46 (72.4 to 98.6)	5.2 (-3.9 to 14.2)
	8 months	97.52 (79.9 to 115.2)	5.8 (-5.3 to 16.9)	99.04 (84.9 to 113.4)	2.4 (-5.6 to 10.5)	88.54 (75.3 to 101.8)	0.1 (-9.4 to 9.7)	78.98 (62.5 to 95.5)	-3.3 (-13.2 to 6.6)
	Peak value	108.84 (94.3 to 123.4)	11.8 (1.8 to 21.8)	105.37 (91.6 to 119.2)	9.2 (2.4 to 16.0)	103.23 (91.7 to 114.8)	4.6 (-3.6 to 12.6)	88.35 (73.9 to 102.7)	2.2 (-6.6 to 10.9)
	Peak time-months	3.76 (2.1 to 5.4)		4.83 (3.8 to 5.9)		3.17 (1.9 to 4.5)		4.14 (2.8 to 5.5)	
QUADs DOM	Baseline	18.13 (15.4 to 20.9)		19.68 (16.9 to 22.4)		17.66 (15.6 to 19.7)		17.71 (15.1 to 20.3)	
	2 months	21.20 (18.4 to 24.0)	4.6 (2.6 to 6.7)	22.10 (19.4 to 24.8)	2.7 (1.2 to 4.2)	20.09 (17.9 to 22.3)	2.8 (1.2 to 4.3)	20.25 (17.6 to 22.9)	2.4 (0.4 to 4.4)
	8 months	23.64 (19.7 to 27.6)	5.4 (3.2 to 7.7)	22.59 (19.6 to 25.6)	3.5 (1.8 to 5.1)	20.62 (17.9 to 23.4)	3.5 (1.9 to 5.2)	21.10 (17.5 to 24.7)	3.2 (1.0 to 5.3)
	Peak value	23.99 (20.4 to 27.5)	5.2 (3.1 to 7.3)	23.63 (20.7 to 26.5)	3.1 (1.7 to 4.5)	21.64 (19.1 to 24.2)	3.2 (1.8 to 4.6)	21.98 (18.8 to 25.2)	2.9 (0.9 to 4.8)

For peer review only

Supplementary Table 2- Average predictions for main outcomes at main time points

	**Peak time-months**	6.44 (4.2 to 8.7)		5.29 (4.0 to 6.6)		5.31 (4.0 to 6.6)		5.50 (3.8 to 7.2)	
**SGRQ-I**	**Baseline**	44.01 (33.2 to 54.9)		38.42 (27.6 to 49.2)		55.00 (47.0 to 63.0)		37.75 (27.6 to 47.9)	
	**2 months**	37.36 (26.4 to 48.3)	- 8.4 (-16.1 to -0.7)	32.11 (21.5 to 42.7)	-6.6 (-12.0 to -1.2)	49.72 (41.5 to 58.0)	-3.5 (-9.6 to 2.5)	31.38 (21.2 to 41.6)	- 6.5 (-13.9 to 0.9)
	**8 months**	40.39 (27.4 to 53.4)	3.1 (- 10.9 to 4.7)	36.18 (25.2 to 47.2)	-1.3 (-6.7 to 4.2)	56.87 (47.6 to 66.1)	1.8 (-4.3 to 7.8)	35.28 (23.2 to 47.4)	-1.2 (-8.7 to 6.35)
	**Min value**	34.43 (22.6 to 46.3)	-6.2 (-13.7 to 1.3)	29.59 (18.6 to 40.6)	- 4.6 (-9.7 to 0.5)	48.25 (39.3 to 57.2)	- 1.9 (-7.8 to 3.8)	28.80 (17.8 to 39.8)	-4.4 (-11.7 to 2.8)
	**Min time-months**	4.47 (3.2 to 5.8)		4.29 (3.5 to 5.1)		3.76 (2.9 to 4.7)		4.32 (3.2 to 5.5)	

Data are mean (95% CI)

For peer review only

Supplementary Figure 1- Changes in outcomes by intervention and clinical groups

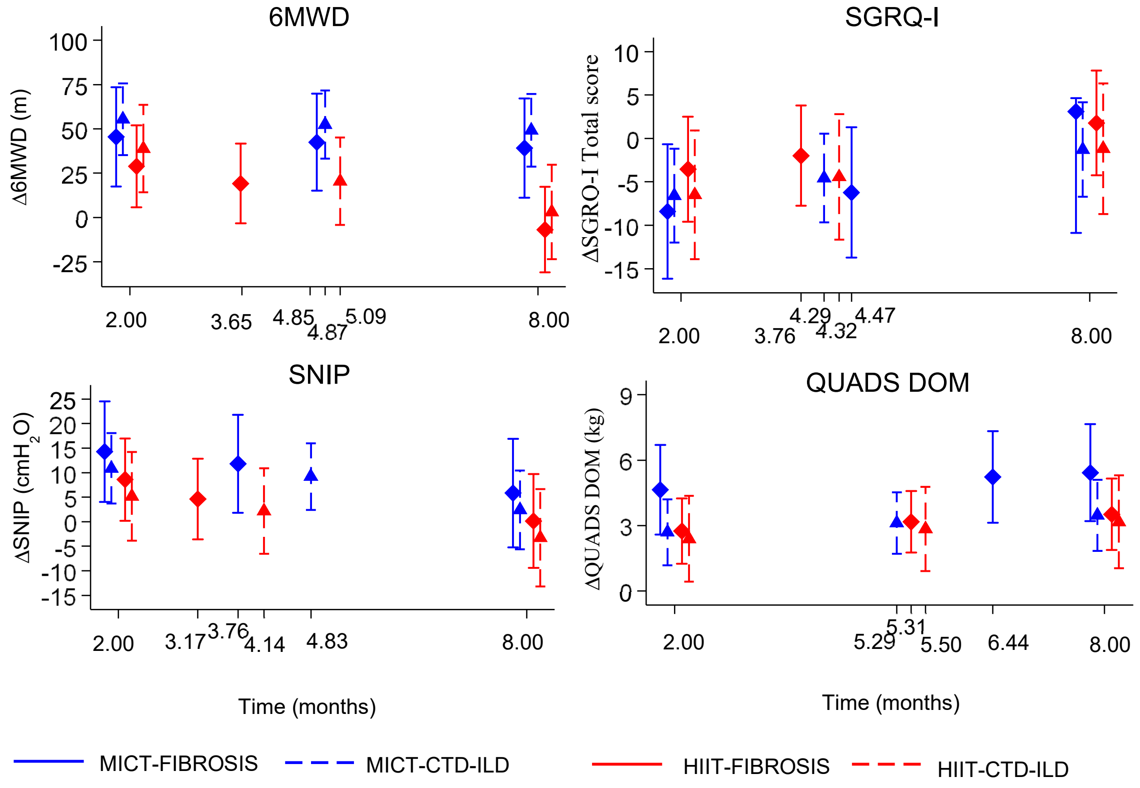

Figure 1: Changes in key outcomes by intervention and ILD clinical groups

Supplementary Figure 2- Random effects models for 6MWD

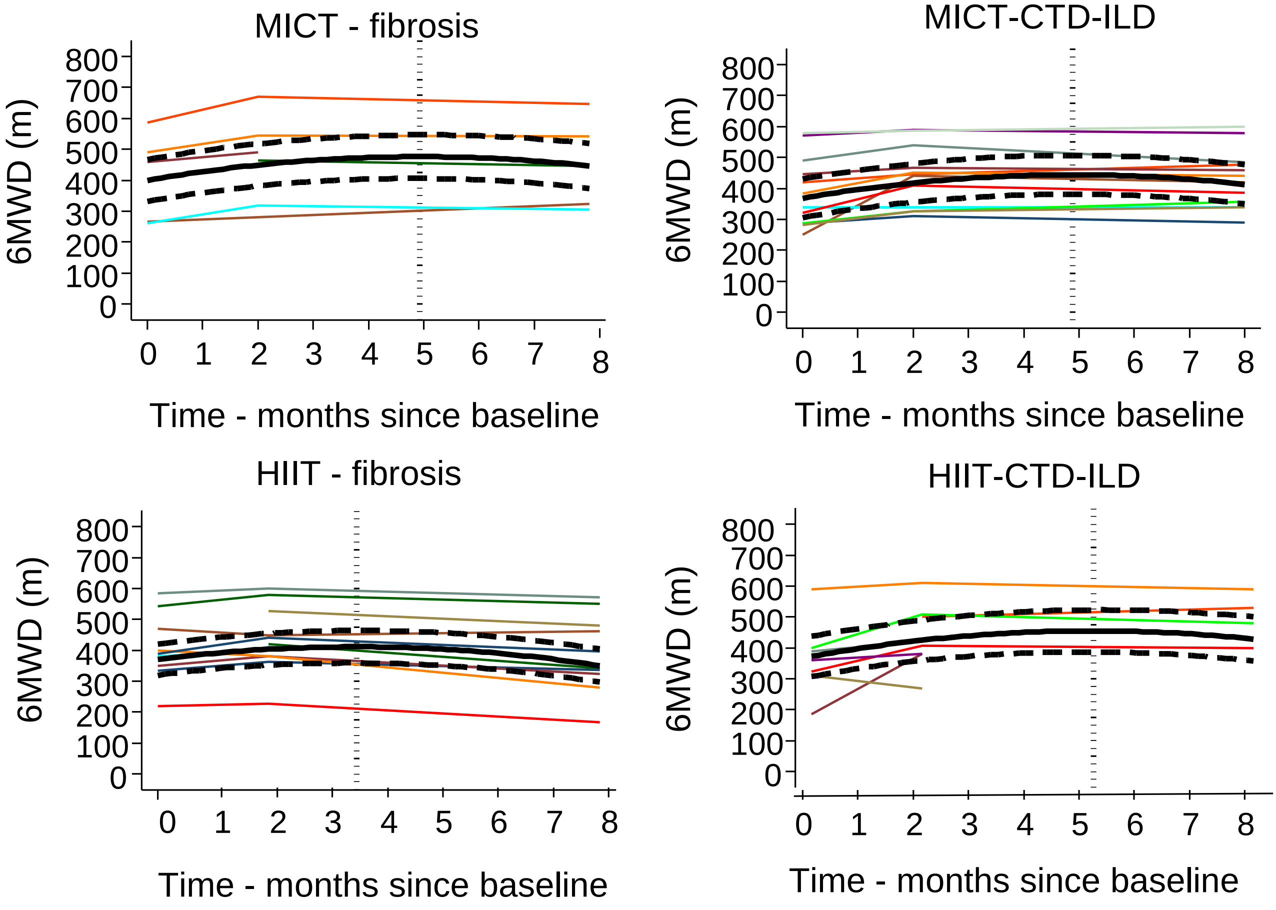

Figure 2: Random effects models for the 6MWD by intervention (HIIT vs MICT) and ILD clinical subgroup (Fibrosis vs CTD-ILD)

For peer review only - http://bmjopen.bmj.com/site/about/guidelines.xhtml

Supplementary Figure 3- Random effect models for SNIP

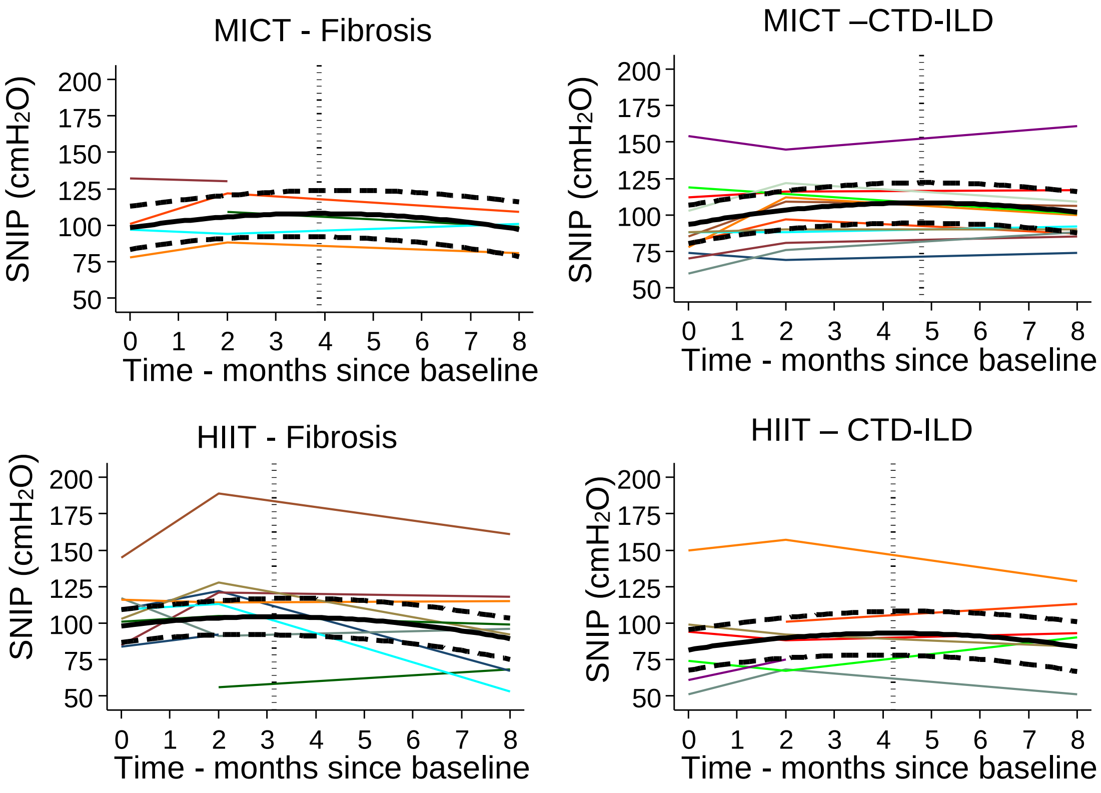

Figure 3: Random effects models for SNIP by intervention (HIIT vs MICT) and ILD clinical subgroup (Fibrosis vs CTD-ILD)

v only

Supplementary Figure 4- Random effects models for Quads-dom

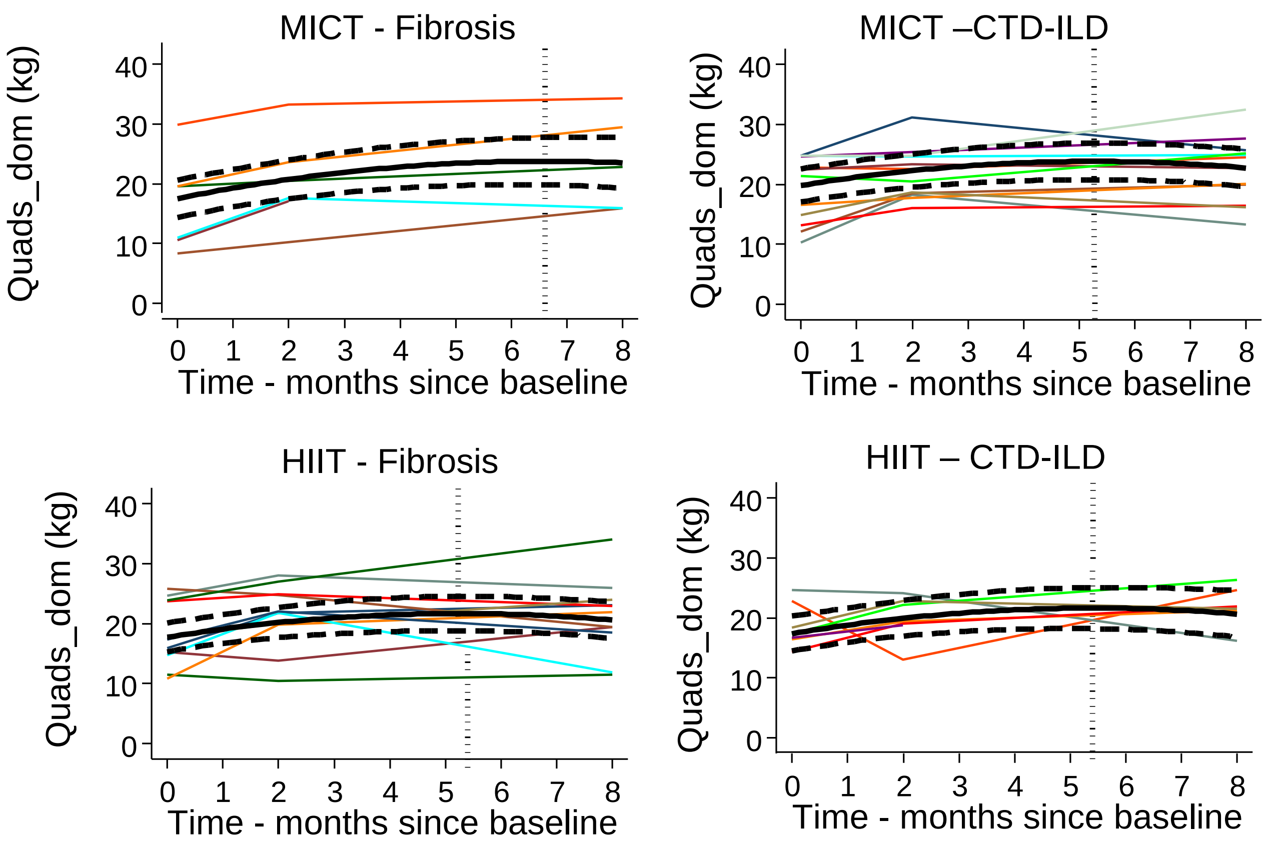

Figure 4: Random effects models for quadriceps strength-dominant side (Quad_dom) by MICT vs HIIT and ILD clinical subgroups.

view only

Supplementary file 1- Development of the ILD Booklet

All pulmonary rehabilitation programmes in the UK include an hour of education, usually at the end of the exercise session. This is a series of presentations and discussions on topics related to the disease(s) included in the programme. In our PR programme, we had a series of topics related to ILD, which included:

Topics about breathing and breathlessness:

- Mechanics of breathing,
- Helpful/unhelpful breathing patterns,
- The Active Cycle of Breathing technique,
- Useful positions for recovering,
- Pacing/ conserving energy in everyday activities

Topics about ILD:

- What happens with ILD,
- Cough- techniques that help
- Associated conditions-rheumatoid Arthritis, Gastro-Oesophageal Reflux disease, Sjögren's syndrome, Bird Fancier's Lung, Idiopathic Pulmonary Fibrosis

Topics about managing the condition:

- Why healthier lifestyle is important,
- Keeping a good posture,
- Barriers to activity/exercise,
- Dietary advice,
- Smoking and smoking cessation,
- Incontinence and advice on pelvic floor exercises
- Oxygen and traveling advice
- Relaxation information,
- Mood, anxiety and breathlessness,
- Self-management and goal setting.

Our 'ILD booklet' was a summary of the key points in the topics discussed. The booklet was put together by the two physiotherapists (included as authors in this manuscript) and the accuracy of the topics was checked by the health professionals who presented each session. For e.g. the topic on 'dietary advice for ILD' was checked by the dietitian in our hospital.

The booklet also included the exercises that were part of the PR programme, with pictures as reminders (the physiotherapists took pictures of themselves to show ILD patient the correct positioning during exercises) and how to adapt exercises in or around the home environment.

For peer review only

Supplementary file 1- Development of the ILD Booklet

Information on how to record progress during PR and in the 6-month period was presented with examples and copies of the breathlessness and exertion BORG scales:

Borg Breathlessness Scale (BRE)

0	Nothing at all
0.5	Very, very slight (just noticeable)
1	Very Slight
2	Slight
3	Moderate
4	Somewhat Severe
5	Severe
6	
7	Very Severe
8	
9	Very, very Severe (Almost Maximal)
10	Maximal

Borg Rate of Perceived Exertion (RPE)

6	No exertion at all
7	Extremely light
8	
9	Very light
10	
11	Light
12	
13	Somewhat Hard
14	
15	Hard (heavy)
16	
17	Very Hard
18	
19	Extremely Hard
20	Maximal Exertion

Example on how to record the information in your worksheet:

Date		BRE	RPE	SpO2	PR
	Start				
Treadmill	4:00 at 3kph	5	14	96	104

During PR sessions, participants used the workout sheet below to record their progress. The booklet included many empty sheets so that participants could record their workouts over the 6-month programme too.

For peer review only

Supplementary file 1- Development of the ILD Booklet

Name: NHS No: Transport Y/N Interpreter: Y/N
O2: Aid Supervision ILD Type:
THR: ID: Sheet:

Date		BRE	RPE	SpO2	PR		BRE	RPE	SpO2	PR		BRE	RPE	SpO2	PR		BRE	RPE	SpO2	PR
	Start					Start					Start					Start				
Treadmill																				
Brisk walk																				
Bike																				
Trampet																				
Punch ups																				
Step ups																				
Knee raise																				
Hip ext																				
STS/Knee ext																				
Bicep curls																				
Arms to side																				
Wall push ups																				
	Middle					Middle					Middle					Middle				
	End					End					End					End				

The booklet also included information about community gyms and leisure centres around South London, sources of support for patients and carers (e.g. advice for carers, disability benefits helpline, citizen's advice, charities) and useful links for organised walks in parks.

Finally, the booklet included the contact numbers of the physiotherapists so that participants could call to ask questions if needed.

Supplementary file 2- Analysis of missing data patterns

Frequencies of the patterns of missing data overall – most common pattern highlighted.

PATTERN	6MWD	SGRQ-I	SNIP	QUADS_DOM
+++	26	33	27	32
++.	6	2	3	2
+.+	1	1	1	2
.++	4	0	3	0
**+..**	**19**	**21**	**18**	**20**
..+	0	0	1	0
.+.	0	1	1	1
...	2	0	4	1
TOTAL MISSING	32	25	31	26

Frequencies of the patterns of missing data stratified by intervention group – most common pattern highlighted.

	CONTROL (MICT)				INTERVENTION (HIIT)			
PATTERN	6MWD	SGRQ-I	SNIP	Q_DOM	6MWD	SGRQ-I	SNIP	Q_DOM
+++	15	16	14	15	11	17	13	17
++.	1	1	1	1	5	1	2	1
+.+	1	1	1	2	0	0	0	0
.++	1	0	1	0	3	0	2	0
**+..**	**6**	**7**	**6**	**7**	**13**	**14**	**12**	**13**
..+	0	0	1	0	0	0	0	0
.+.	0	0	0	0	0	1	1	1
...	1	0	1	0	1	0	3	1
TOT MISS	10	9	11	10	22	16	20	16

Frequencies of the patterns of missing data stratified by intervention group and clinical group – most common pattern highlighted.

	CONTROL (MICT)								INTERVENTION (HIIT)							
	FIBROSIS				AUTOIMMUNE				FIBROSIS				AUTOIMMUNE			
PATTERN	6MWD	SGRQ-I	SNIP	Q_DOM	6MWD	SGRQ-I	SNIP	Q_DOM	6MWD	SGRQ-I	SNIP	Q_DOM	6MWD	SGRQ-I	SNIP	Q_DOM
+++	3	4	3	4	12	12	11	11	8	11	8	11	3	6	5	6
++.	1	1	1	1	0	0	0	0	1	0	1	0	4	1	1	1
+.+	1	1	0	1	0	0	1	1	0	0	0	0	0	0	0	0
.++	1	0	1	0	0	0	0	0	2	0	1	0	1	0	1	0
**+..**	**5**	**5**	**5**	**5**	**1**	**2**	**1**	**2**	**9**	**9**	**8**	**8**	**4**	**5**	**4**	**5**
..+	0	0	1	0	0	0	0	0	0	0	0	0	0	0	0	0
.+.	0	0	0	0	0	0	0	0	0	0	0	0	0	1	1	1
...	0	0	0	0	1	0	1	0	0	0	1	1	1	0	1	0
TOTAL MIS	8	7	8	7	2	2	3	3	12	9	11	9	10	7	8	7

For peer review only - http://bmjopen.bmj.com/site/about/guidelines.xhtml

Supplementary file 2- Analysis of missing data patterns

Analysis of the missing data for each outcome.

	**6MWD**				**SGRQ-I**				**SNIP**				**QUADS DOM**			
	OR	p-value	95%CI		OR	p-value	95%CI		OR	p-value	95%CI		OR	p-value	95%CI	
**MODELS ON TIME ONLY**																
**Time**	4.575	<.001	1.948	10.742	137.057	<.001	11.737	1600.393	4.132	0.001	1.814	9.412	76.820	<.001	8.511	693.382
**Time^2**	0.867	0.002	0.791	0.950	0.617	<.001	0.481	0.791	0.872	0.003	0.796	0.954	0.650	<.001	0.519	0.814
**MODELS ON INTERVENTION GROUP ADJUSTED FOR TIME AND TIME^2**																
**Intervention**	3.147	**0.108**	0.776	12.759	6.168	**0.212**	0.353	107.623	3.012	**0.172**	0.620	14.634	5.016	**0.259**	0.305	82.425
**MODELS ON INTERVENTION GROUP ADJUSTED FOR TIME AND TIME^2**																
**Intervention**	2.896	**0.135**	0.719	11.659	4.942	**0.276**	0.279	87.421	2.622	**0.224**	0.554	12.420	4.069	**0.324**	0.251	66.002
**Clinical**	0.459	**0.258**	0.119	1.769	0.140	**0.182**	0.008	2.506	0.304	**0.133**	0.064	1.439	0.167	**0.210**	0.010	2.745

OR= odds ratios

Missing observations were denoted by 1 if missing and 0 otherwise, which generated a longitudinal binary outcome which has been analysed using binary mixed models, i. e. accounting for the longitudinal aspect of the data.

The results are consistent with no pattern in the missing data across intervention and/or clinical groups and missingness is most likely attributable to the follow-up time.

For peer review only

Supplementary File 3-Additional analysis on the effect of DLCO on key outcomes

Post-hoc statistical analysis on the effect of baseline DLCO on key outcomes

We have investigated DLCO in a number of different ways.

1. Baseline assessment, i.e. DLCO by intervention and clinical groups simultaneously.

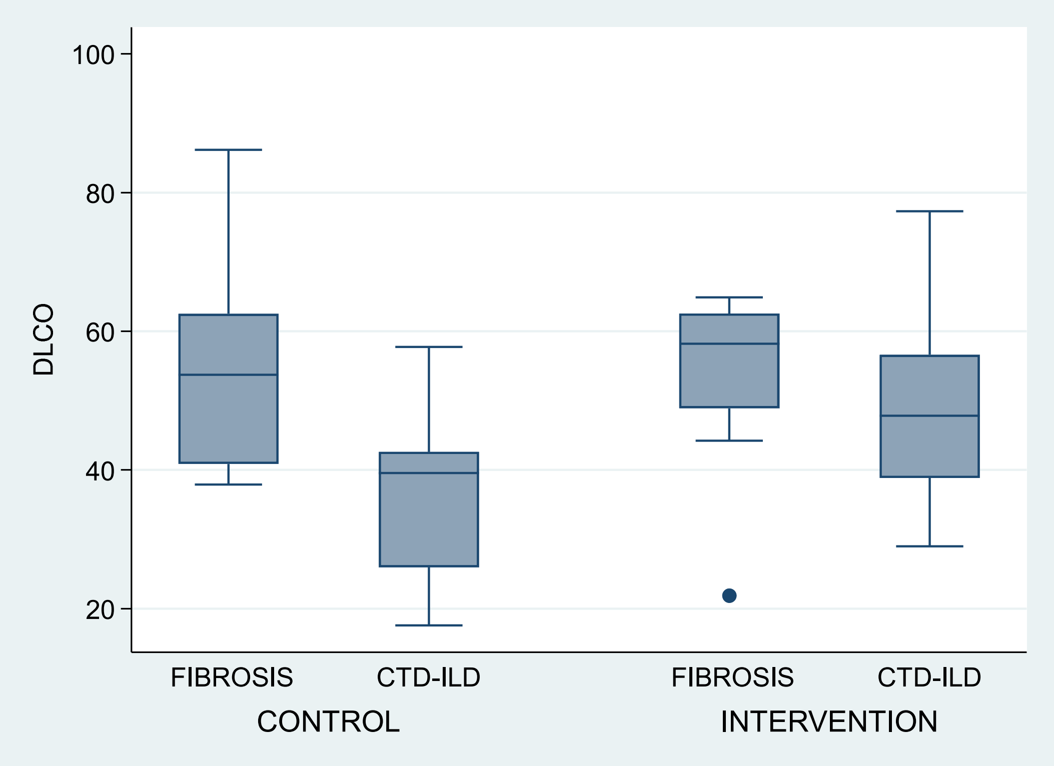

Based on these data, the evidence suggests that DLCO differs by intervention group (adjusted p value=0.003)– finding initially deems as random randomization imbalance. Nevertheless, there is not enough evidence to suggest that DCLO levels differ by clinical group, i.e. the DCLO difference between the clinical groups seems to remain similar with the intervention group (p=0.069).

2. Relationship between the 4 outcomes and DLCO at the baseline. The results are presented in Table 1 below. There was no evidence for an interaction of DLCO with either intervention or clinical group regarding these outcomes. Only coefficients corresponding to DLCO are shown for a succinct message, but they are adjusted for intervention groups and clinical groups.

Table 1: Relationship between the 4 key outcomes and baseline DLCO, adjusted for intervention and clinical groups.

	Estimate	SE	T value	p-value	95%CI low	95%CI high	No Obs	Association
MWT	.9787011	1.178773	0.83	0.410	-1.39138	3.348783	50	UNADJUSTED
SNIP	.1441871	.2431136	0.59	0.556	-.3454688	.633843	47	
QUADS DOM	-.0956757	.0499201	-1.92	**0.061**	-.1958477	.0044964	54	
SGRQI	-.0614169	.1979514	-0.31	0.758	-.4586354	.3358016	54	
MWT	8799743	1.31952	0.67	0.508	-1.774558	3.534506	50	ADJUSTED FOR
SNIP	.0873187	.2717802	0.32	0.750	-.4604183	.6350556	47	INTERVENTION
QUADS DOM	-.123836	.0548133	-2.26	**0.028**	-.2338782	-.0137938	54	GROUP

Supplementary File 3-Additional analysis on the effect of DLCO on key outcomes

SGRQI	.0017764	.2204537	0.01	0.994	-.4408028	.4443556	54	
MWT	1.047737	1.366581	0.77	0.447	-1.703048	3.798522	50	ADJUSTED FOR
SNIP	.1784858	.2751485	0.65	0.520	-.3764041	.7333758	47	INTERVENTION
QUADS DOM	-.1468699	.0553319	-2.65	**0.011**	-.2580074	-.0357325	54	AND CLINICAL
SGRQI	.0816139	.2234533	0.37	0.716	-.3672054	.5304331	54	GROUP
MWT	1.078732	1.302553	0.83	0.412	-1.544745	3.702209	50	ADJUSTED FOR
SNIP	.181307	.2570293	0.71	0.484	-.337399	.7000131	47	INTERVENTION,
QUADS DOM	-.1478509	.0529411	-2.79	**0.007**	-.25424	-.0414617	54	CLINICAL
SGRQI	.0764789	.2163264	0.35	0.725	-.3582453	.5112032	54	GROUP AND AGE

MWT=distance in the 6MWT, SNIP=Sniff nasal inspiratory pressure, QUADS DOM=Quadriceps strength in the dominant leg and SGRQ-I=total score in the St George's Respiratory Questionnaire for ILD

There is some evidence to suggest that, at baseline, the DLCO is negatively associated with QUADS DOM but, based on these data, none of the other important measurements seemed to have been affected.

3. However, when analysing the data longitudinally, after adjusting for time and its quadratic term, interactions between time and intervention and clinical groups and age, the effect on the QUADS DOM seem to weaken. Below, we additionally adjust the models presented in Table 1 by DLCO – after investigating its interactions with intervention/clinical groups too. Only coefficients corresponding to DLCO are shown for a succinct message, but they are adjusted for time, intervention groups and clinical groups, age and their interactions.

Table 2: Relationship between DLCO and key outcomes, adjusted for time, intervention groups and clinical groups, age and their interactions.

	Estimate	SE	T value	p-value	95%CI low	95%CI high	No Obs	Association
MWT	1.336415	.9914012	1.35	0.178	-.6066961	3.279525	113/53	ADJUSTED FOR
SNIP	.3109333	.2361878	1.32	0.188	-.1519863	.7738529	113/53	INTERVENTION,
QUADS DOM	-.0910743	.0473067	-1.93	**0.054**	-.1837937	.0016452	119/55	CLINICAL
SGRQI	.1404341	.197889	0.71	0.478	-.2474212	.5282893	120/55	GROUP AND AGE

MWT=distance in the 6MWT, SNIP=Sniff nasal inspiratory pressure, QUADS DOM=Quadriceps strength in the dominant leg and SGRQ-I=total score in the St George's Respiratory Questionnaire for ILD

This is not to say that the effect on these outcomes may not be important, we just did not capture it based on these data.

For peer review only

Reporting checklist for randomised trial.

Based on the CONSORT guidelines.

Instructions to authors

Complete this checklist by entering the page numbers from your manuscript where readers will find each of the items listed below.

Your article may not currently address all the items on the checklist. Please modify your text to include the missing information. If you are certain that an item does not apply, please write "n/a" and provide a short explanation.

Upload your completed checklist as an extra file when you submit to a journal.

In your methods section, say that you used the CONSORTreporting guidelines, and cite them as:

Schulz KF, Altman DG, Moher D, for the CONSORT Group. CONSORT 2010 Statement: updated guidelines for reporting parallel group randomised trials

		Reporting Item	Page Number
**Title and Abstract**			
Title	#1a	Identification as a randomized trial in the title.	1
Abstract	#1b	Structured summary of trial design, methods, results, and conclusions	2
**Introduction**			
Background and objectives	#2a	Scientific background and explanation of rationale	4

For peer review only

Background and objectives	#2b	Specific objectives or hypothesis	5
**Methods**			
Trial design	#3a	Description of trial design (such as parallel, factorial) including allocation ratio.	5 and Figure 1
Trial design	#3b	Important changes to methods after trial commencement (such as eligibility criteria), with reasons	n/a
Participants	#4a	Eligibility criteria for participants	5
Participants	#4b	Settings and locations where the data were collected	5
Interventions	#5	The experimental and control interventions for each group with sufficient details to allow replication, including how and when they were actually administered	6-7
Outcomes	#6a	Completely defined prespecified primary and secondary outcome measures, including how and when they were assessed	8-9
Sample size	#7a	How sample size was determined.	9
Sample size	#7b	When applicable, explanation of any interim analyses and stopping guidelines	n/a

For peer review only

Randomization - Sequence generation	#8a	Method used to generate the random allocation sequence.	6
Randomization - Sequence generation	#8b	Type of randomization; details of any restriction (such as blocking and block size)	6
Randomization - Allocation concealment mechanism	#9	Mechanism used to implement the random allocation sequence (such as sequentially numbered containers), describing any steps taken to conceal the sequence until interventions were assigned	6
Randomization - Implementation	#10	Who generated the allocation sequence, who enrolled participants, and who assigned participants to interventions	6
Blinding	#11a	If done, who was blinded after assignment to interventions (for example, participants, care providers, those assessing outcomes) and how.	6
Blinding	#11b	If relevant, description of the similarity of interventions	6 and table 1
Statistical methods	#12a	Statistical methods used to compare groups for primary and secondary outcomes	9
Statistical methods	#12b	Methods for additional analyses, such as subgroup analyses and adjusted analyses	9

For peer review only

Outcomes	#6b	Any changes to trial outcomes after the trial commenced, with reasons	n/a
**Results**			
Participant flow diagram (strongly recommended)	#13a	For each group, the numbers of participants who were randomly assigned, received intended treatment, and were analysed for the primary outcome	10-Figure 1
Participant flow	#13b	For each group, losses and exclusions after randomization, together with reason	Fig 1
Recruitment	#14a	Dates defining the periods of recruitment and follow-up	Fig 1
Recruitment	#14b	Why the trial ended or was stopped	n/a
Baseline data	#15	A table showing baseline demographic and clinical characteristics for each group	Table 1
Numbers analysed	#16	r	10 -Fig 1 and Table 1
Outcomes and estimation	#17a	For each primary and secondary outcome, results for each group, and the estimated effect size and its precision (such as 95% confidence interval)	10-11

For peer review only

Outcomes and estimation	#17b	For binary outcomes, presentation of both absolute and relative effect sizes is recommended	10-11
Ancillary analyses	#18	Results of any other analyses performed, including subgroup analyses and adjusted analyses, distinguishing pre-specified from exploratory	Supplementary files Tables 3-4 and Figures 4-5-6
Harms	#19	All important harms or unintended effects in each group (For specific guidance see CONSORT for harms)	Fig.1
**Discussion**			
Limitations	#20	Trial limitations, addressing sources of potential bias, imprecision, and, if relevant, multiplicity of analyses	15
Interpretation	#22	Interpretation consistent with results, balancing benefits and harms, and considering other relevant evidence	12-16
Registration	#23	Registration number and name of trial registry	1
Generalisability	#21	Generalisability (external validity, applicability) of the trial findings	2, 15-16
**Other information**			

For peer review only

Interpretation	#22	Interpretation consistent with results, balancing benefits and harms, and considering other relevant evidence	12-16
Registration	#23	Registration number and name of trial registry	1
Protocol	#24	Where the full trial protocol can be accessed, if available	5 and Protocol file
Funding	#25	Sources of funding and other support (such as supply of drugs), role of funders	21

Notes:

- 13a: 10-Figure 1
- 16: 10 -Fig 1 and Table 1
- 18: Supplement Tables 3-4, Fig 4-5-6 The CONSORT checklist is distributed under the terms of the Creative Commons Attribution License CC-BY. This checklist was completed on 29. March 2022 using https://www.goodreports.org/, a tool made by the EQUATOR Network in collaboration with Penelope.ai

For peer review only
